# Distilling functional variations for human *UGT2B4* upstream region based on selection signals and implications for phenotypes of Neanderthal and Denisovan

**DOI:** 10.1038/s41598-023-29682-x

**Published:** 2023-02-23

**Authors:** Pin-Yi Wang, Yuan Yang, Xiao-Qian Shi, Ying Chen, Shao-Dong Liu, Hong-Yan Wang, Tao Peng, Qiang Shi, Wei Zhang, Chang Sun

**Affiliations:** 1 College of Life Sciences, Shaanxi Normal University, Xi’an, 710119 Shaanxi People’s Republic of China; 2 State Key Laboratory for Conservation and Utilization of Bio-Resources in Yunnan, Yunnan University, Kunming, 650091 Yunnan People’s Republic of China; 3 Department of Preventive Medicine, Northwestern University Feinberg School of Medicine, Chicago, IL 60611 USA; 4 Institute of Precision Medicine, Jining Medical University, Jining, 272067 Shandong People’s Republic of China

**Keywords:** Evolution, Evolutionary genetics, Cancer, Cancer genetics, Evolutionary biology, Functional genomics, Gene expression

## Abstract

Our previous work identified one region upstream human *UGT2B4* (UDP glucuronosyltransferase family 2 member B4) which is associated with breast cancer and under balancing selection. However, the distribution, functional variation and molecular mechanism underlying breast cancer and balancing selection remain unclear. In current study, the two haplotypes with deep divergence are described by analyzing 1000 genomes project data and observed to be with high frequencies in all human populations. Through population genetics analysis and genome annotation, the potential functional region is identified and verified by reporter gene assay. Further mutagenesis indicates that the functional mutations are rs66862535 and rs68096061. Both SNPs can alter the interaction efficiency of transcription factor POU2F1 (POU class 2 homeobox 1). Through chromosome conformation capture, it is identified that the enhancer containing these two SNPs can interact with *UGT2B4* promoter. Expression quantitative trait loci analysis indicates that *UGT2B4* expression is dependent on the genotype of this locus. The common haplotype in human is lost in four genomes of archaic hominins, which suggests that Neanderthal and Denisovan should present relatively lower *UGT2B4* expression and further higher steroid hormone level. This study provides new insight into the contribution of ancient population structure to human phenotypes.

## Introduction

Balancing selection is a rare phenomenon in human genome^1–3^ and observed in some non-coding regions^4–8^. Usually, these regions under balancing selection are supposed to regulate gene expression. However, the function of these non-coding regions has hardly been investigated, which is partially hampered by the linkage disequilibrium (LD) pattern. Indeed, each region under balancing selection usually spans long distance and is composed of multiple SNPs in nearly complete LD. Therefore, these SNPs will present similar signals in disease/phenotype case-control and expression quantitative trait locus (eQTL) study, including genome-wide ones. As a result, identifying the functional SNP(s) and elucidating their regulatory mechanisms remain a major challenge for human genetics.

Breast cancer is the most common malignant tumor and cancer-related mortality in females worldwide^9^. The predisposing factors of breast cancer include genetic susceptibility and many environmental factors, including but not limited to alcohol intake, obesity and steroid hormone level^10^. Among them, cumulative steroid hormone (especially estrogen) exposure plays an essential role in onset of breast cancer^11^. Steroid hormone level in breast cancer tissues has been observed to be higher than that in normal ones^12^. Moreover, multiple steroid hormone analogs and aromatase inhibitors have been widely used in clinical treatment of this disease^13^.

Glucuronidation is an essential reaction for the clearance and detoxification of numerous endogenous and exogenous compounds, including steroid hormones, bilirubin, thyroid hormones and some clinical drugs^14^. In this reaction, UDP (Uridine diphosphate)-glucuronic acid is attached to the substrates, which can increase substrates hydrophilicity and make them easier to be removed from human body^14^. This reaction is catalyzed by an enzyme family, UDP-glucuronosyltransferase (UGT). This family includes 22 active members in human and can be divided into four major subfamilies, UGT1, UGT2, UGT3 and UGT8, according to chromosome location, gene structure and sequence similarity^15^. Considering the function of UGT in steroid hormones metabolism, it has been suggested that variations in the UGT genes, especially the ones that can decrease enzyme amount or activity and further increase individual sex hormone exposure, are involved in breast cancer onset^16–19^.

Among this family, *UGT2B4* (UDP glucuronosyltransferase family 2 member B4) is the most highly expressed member and occupies ~ 44.5% of all UGT transcript in liver^20^. In the upstream region of *UGT2B4*, two haplotypes with deep divergence (~ 1.3 million years ago) have been identified based on partial region (~ 4.8 kb in total) resequencing^4^. Due to the excess of common variations and thus a strong positive Tajima’s *D*, it has been suggested that this region is under balancing selection^4^. Due to the significantly higher frequency of the rare haplotype in breast cancer patients, it has been suggested that this haplotype can increase individual breast cancer risk^4^. However, these haplotypes have not been fully described, which also hinders the disclosure of their origin and the molecular mechanism underlying breast cancer and natural selection.

Neanderthal and Denisovan are archaic hominins living in Eurasia ~ 400–40 k years ago and we know little about their gene expression and phenotypes except skeletal system. One indirect way to investigate these is through their genomes, especially the segments highly similar with human^21^. Since the origin of this mutation pattern upstream *UGT2B4* predates the divergence of human and Neanderthal/Denisovan (~ 550–765 k years ago)^22^, archaic hominins should inherit at least one of the two haplotypes. Therefore, investigating the function of these two halotypes might shed more light on the gene expression and related phenotypes of archaic homonins.

In the present study, we describe the location and distribution of these haplotypes through data analysis of 1000 genomes (1000G) project. Through evolutionary analysis, genome annotation and functional genomics approaches, the functional SNPs and underlying mechanism are identified.

## Results

### LD pattern upstream human *UGT2B4*

**Figure 1:**
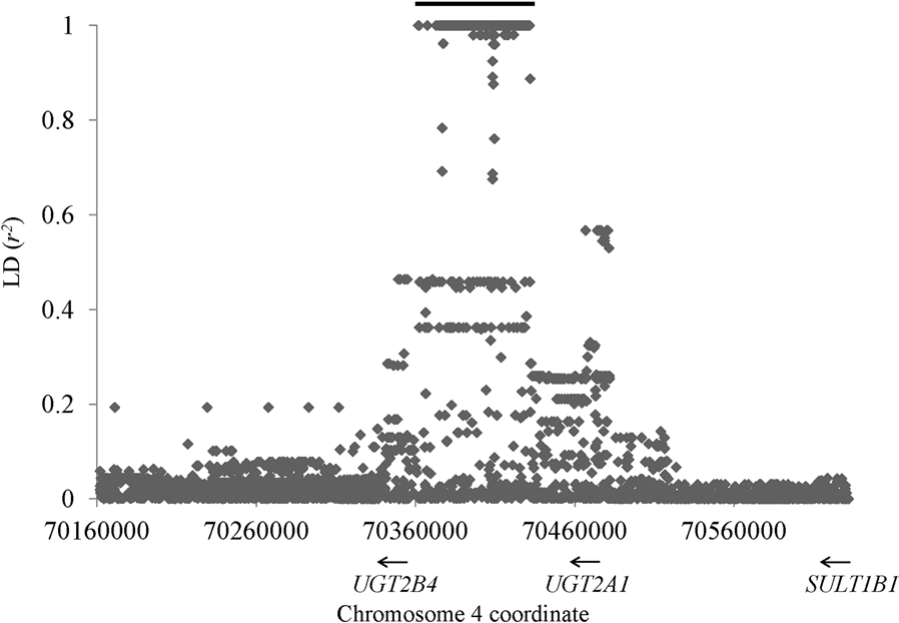
LD pattern in *UGT2B4* region. The *x* axis indicates chr4 coordinate while *y* axis denotes the *r*^*2*^ value with the tag SNP rs11723463. Each diamond represents one genetic variation. The bar in top points out the position of the long LD region. The horizontal arrows in bottom denote the schematic location and transcript orientation of the genes in this region.

In all 26 populations from 1000G, there is a long LD block (a group of genetic variations with pairwise *r*^*2*^ ≥ 0.8) upstream *UGT2B4* but with different length between African and non-African populations (see Supplementary Table 1 for detail). In most non-African populations, the LD block spans ~ 70.3 kb, ranging from 70361741 (relative to chr4; all coordinates in current study are based on human genome build 37 unless otherwise mentioned; rs941389) to 70432006 (rs12511454; see Fig. 1). In this LD block, the first two SNPs, rs941389 and rs13129471, are within *UGT2B4* promoter (-115 and -497 relative to translation start, respectively). In African populations, the LD block covers ~ 54.9 kb, spanning from 70376705 (rs202069806) to 70431614 (rs55735079), which is relatively far from *UGT2B4* (~ 15.1 kb from translation start). Within the LD block, the SNPs are highly shared among populations (see Supplementary Table 1). For simplification, we utilized CEU (Utah Residents with Northern and Western European Ancestry) as an example to perform following analysis.

In UGT cluster, there are two structure variations (SV, or copy number variation), esv3600896 (~ 184.2 kb upstream *UGT2B4*) and esv3600917 (~ 108.5 kb downstream *UGT2B4*), showing signals for balancing selection^23^. We were wondering whether these two SVs are also within our LD block. The LD analysis indicates that in all 1000G populations, esv3600896 and esv3600917 show low (all *r*^*2*^ < 0.098) and moderate (all *r*^*2*^ < 0.45) LD with our locus, respectively. Therefore, these three loci should be independent balancing selection targets.

**Figure 2:**
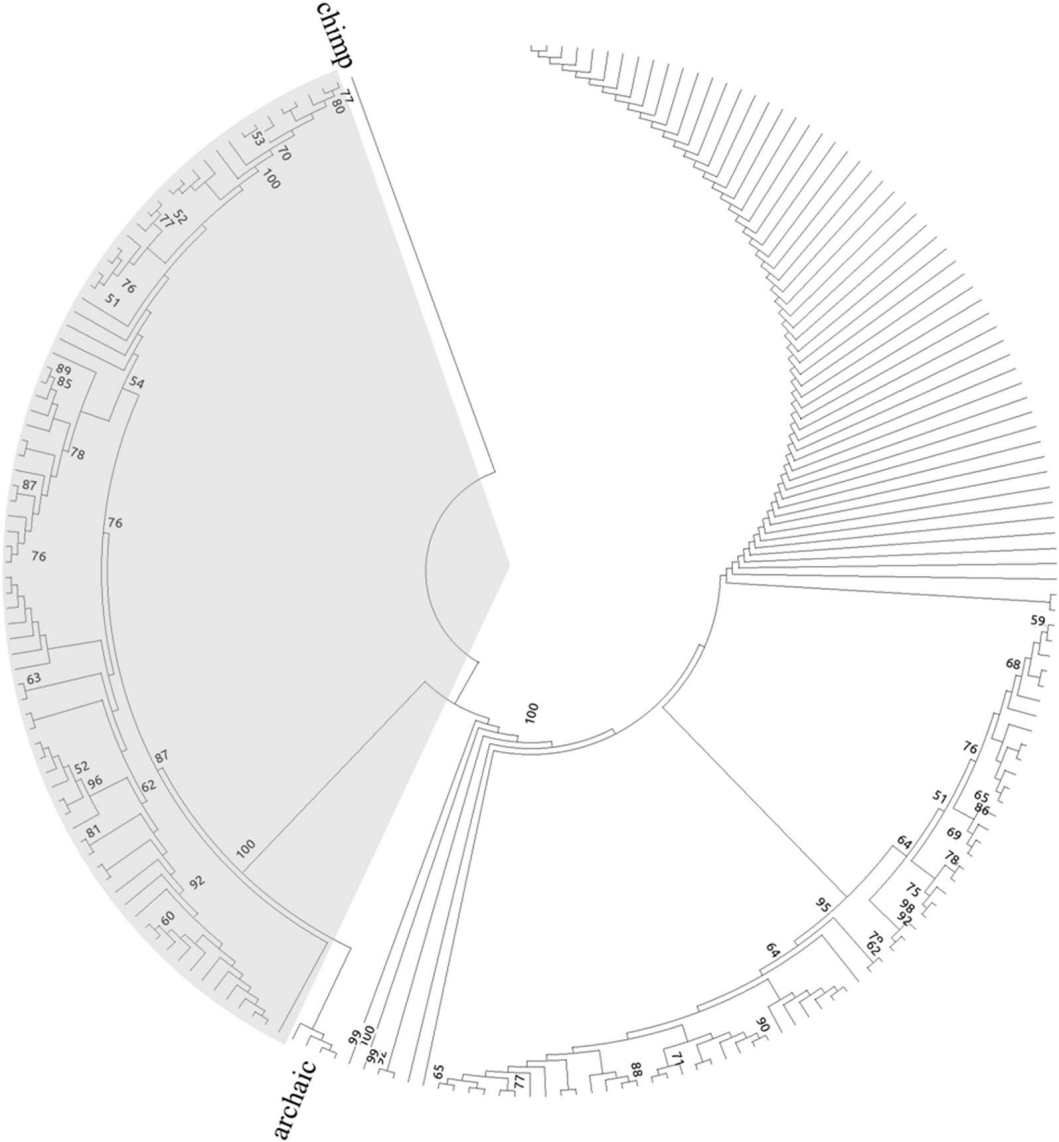
Phylogeny of CEU haplotypes. Chimpanzee sequence is used as an outgroup and labeled as chimp. Archaic clade includes four individuals, from left to right, Denisovan, Altai, Chagyrskaya and Vindija. CEU haplotypes are not labeled. The haplotype 2 lineages are marked by gray background. Arab numbers indicate bootstrap support and only the ones > 50 are displayed.

In CEU population, there are 731 genetic variations (663 SNPs and 68 indels) within this ~ 70.3 kb region and among them, 454 (412 SNPs and 42 indels) are within this LD block (see Supplementary Table 1^1^ and Fig. 1). Due the large number of the genetic variations in this LD block, two haplotypes with deep divergence can be observed (see Supplementary Table 2). In this study, the common and rare haplotype are denoted as haplotype 1 and 2, respectively. As shown in Fig. 2, after splitting from chimpanzee, the CEU lineages form two huge clades, corresponding to haplotypes 1 and 2. This topology is supported by all 100 bootstraps. If we set the common ancestor date of human and chimpanzee at 6 million years ago, the divergence of haplotype 1 and 2 is supposed to be ~ 1.54 million years, which is close to our previous estimation^4^. We further searched GEVA (genealogical estimation of variant age), an approach to calculate SNP age based on population-scale sequencing data^24^. For the 412 SNPs in CEU core haplotype, 363 of them (88.1%) are with an age beyond 1 million years (see Supplementary Fig. 1), which is close to our estimation. The haplotypes from other 1000G populations show a similar phylogeny (results not shown).

### Spatial distribution of these two haplotypes

**Figure 3:**
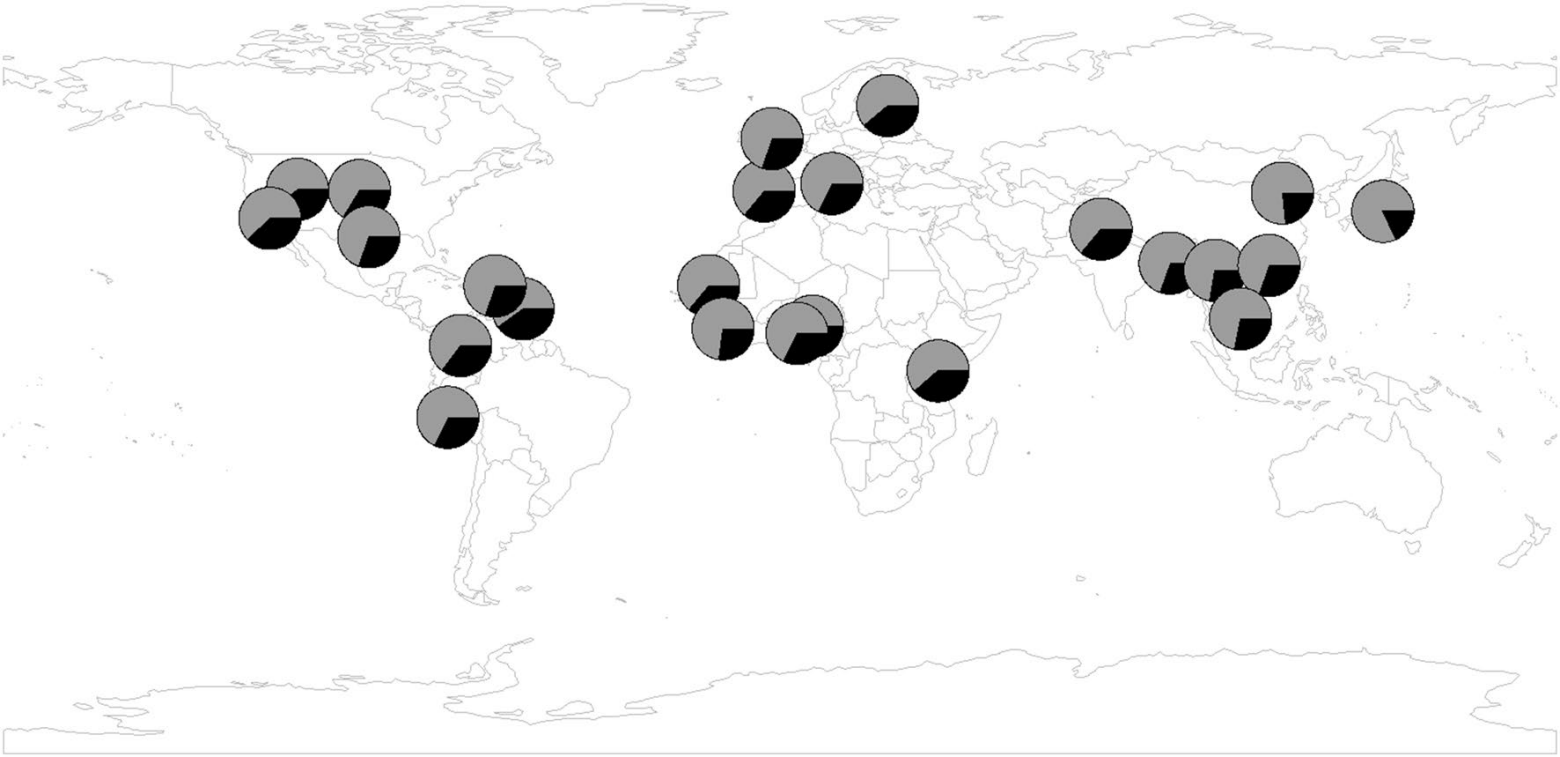
Frequency of the core haplotypes in global populations from 1000G. Each circle represents one population and pie chart displays the haplotype frequency. The gray and black color indicates haplotype 1 and 2, respectively.

To display the distribution of these two haplotypes, we calculated the frequencies in 26 populations from 1000G. As shown in Fig. 3 and Supplementary Table 1, haplotype 2 appears a relatively high frequency in all modern human populations. Its frequency varies from 18.3% in JPT (Japanese in Tokyo, Japan) to 40.6% in ACB (African Caribbean in Barbados). The average frequency is 32.5% for overall 1000G populations. If we divided all populations into five superpopulations according to their origins, similar frequencies could be obtained, i.e., 34.3%, 33.8%, 25.4%, 36.8% and 30.8% for African, Mixed American, East Asian, European and South Asian, respectively.

We also utilized HGDP (Human Genome Diversity Project; https://www.hagsc.org/hgdp/) data to analyze the haplotype distribution. In HGDP dataset, genotype is available for two SNPs in the core haplotype, rs11723463 and rs6844026. As shown in Supplementary Table 3, haplotype 2 exists in all HGDP populations. The average frequency is 31.5%, which is close to the value in 1000G data. However, the frequency spectrum in HGDP is wider than that in 1000G. The lowest frequency is 9.5% in Surui (Brazil) while the highest one is 50.0% in Biaka Pygmy (Central African Republic). However, we caution that the frequencies in HGDP might be not accurate due to the small sample size (< 30 for most populations; see Supplementary Table 3).

### Evidence for balancing selection

It is supposed that this region is under balancing selection^4^. To verify the situation in the whole LD block region, we calculated Tajima’s *D* for all populations. To make the results comparable among populations, only the region containing common SNPs for all 1000G populations, i.e., from rs202069806 to rs7657504, were included. As shown in Supplementary Table 4, all populations present a strong positive Tajima’s *D* and most of them reach statistically significant. These results are consistent with our previous result^4^. Outside the location of this LD block, no clear evidence is obtained for balancing selection.

To avoid the influence of population dynamics, we further searched β score, one method to detect balancing selection based on substitution data^25^, for the SNPs in core haplotype. For all 1000G populations, the SNPs in core haplotype reach top 1% score of the whole genome, thus verifying that this region is indeed under balancing selection.

To investigate the haplotype frequency in human history, we downloaded Allen Ancient DNA Resource v44.3 (https://reich.hms.harvard.edu/). This dataset includes genotype of ~ 1.23 million SNPs for 6642 ancient DNA samples. 15 SNPs in the core haplotype (rs28712359, rs11732996, rs2195847, rs11723463, rs10518062, rs1368120, rs1368119, rs11249448, rs6850489, rs6832277, rs6858812, rs34970019, rs68006105, rs10028160 and rs28834733) are included in this dataset. The genotypes from Neanderthal and Denisovan individuals were excluded from analysis and the haplotype frequency was expressed as the mean frequency for these 15 SNPs. As shown in Supplementary Fig. 2, haplotype 2 frequency have reached ~ 30% in the samples with > 20 k years history and been relatively even until current stage. The sample sizes for the groups > 10 k years are rather small, which might bias the result. However, the sample size increases notably for the groups after this time point. Considering all these facts, it can be speculated that the frequency of haplotype 2 is remaining relatively stable from at least 10 k years ago.

### Genotype of Neanderthal and Denisovan

We compared the 454 genetic variations in CEU core haplotype with the homologous sites in three Neanderthal individuals, Altai^22^, Chagyrskaya^26^ and Vindija^27^, and Denisovan^28^.

In Altai^22^ sequence, genotypes are available for 434 sites (409 SNPs and 25 indels; see Supplementary Table 2). Among them, 7 sites are heterozygous in Altai individual, and at each site, one allele is identical with haplotype 2 (see Supplementary Table 2). For the rest 427 homozygous sites in Altai, 412 (96.5%) are same with haplotype 2 (see Supplementary Table 2).

In Chagyrskaya^26^ and Vindija^27^ sequences, genotypes are available for only 223 sites since a stricter algorithm, snpAD (http://bioinf.eva.mpg.de/snpAD/), is utilized in their data processing (see Supplementary Table 2). Among them, 220 sites (98.7%) are same with haplotype 2 (see Supplementary Table 2).

In Denisovan^28^ sequence, genotypes are available for 437 sites. Among them, 5 sites are heterozygous and at each site, one allele is identical with haplotype 2 (see Supplementary2Table ). For the rest 432 homozygous sites in Denisovan, 420 (97.2%) are same with haplotype 2 (see Supplementary Table 2). All these facts indicate an extremely high similarity between haplotype 2 and Neanderthals or Denisovan sequence and suggest that haplotype 1 is lost in archaic hominins. However, we caution about this conclusion since the sample size for archaic hominins is rather small.

To further disclose the relationship for different sequences, Neanderthals and Denisovan were included in phylogeny reconstruction. As shown in Fig. 2, the three Neanderthal individuals and Denisovan form a monophyletic group with haplotype 2 sequences, which is also supported by all bootstraps. Based on the sequences at this locus, the divergence time between Neanderthals and human haplotype 2 or Denisovan is estimated to be ~ 729 and ~ 420 k years ago, respectively, which are close to the calculations based on whole genome data^22^.

### Genome annotation

Since the LD block starts from *UGT2B4* promoter region, we first hypothesized that these two haplotypes might have different promoter activity. To investigate this possibility, we amplified *UGT2B4* promoter region with different haplotypes from specific individuals, inserted them into pGL3-basic vector and transfected them. Since the nearby genes, *UGT2B4* and *UGT2A1* (UDP glucuronosyltransferase family 2 member A1 complex locus), are mainly expressed in liver, all functional genomics work was performed in liver cell line HepG2. As shown in Supplementary Fig. 3, these two haplotypes failed to present significantly different promoter activity (*P* = 0.41; n = 6 for each group). This result suggests that these two haplotypes might be with different enhancer activity instead.

The LD block is as long as ~ 70 kb. Therefore, it is difficult to identify the functional region which contains the causal SNP(s) for breast cancer and natural selection. Since this region is under balancing selection, we used a sliding window approach to identify the potential functional region within this long LD block. The rationale is that under balancing selection, the functional region should have a larger chance to reach the highest equilibrium frequency than nearby hitchhiking regions. Consequently, the potential functional region should have better chance to present the highest *π* and further Tajima’s *D*.

Therefore, we utilized *π* and Tajima’s *D* as the major indicators to identify the functional region. As shown in Supplementary Fig. 4, multiple peaks are appearing for these two indices in three representative populations in the world, CEU, CHB (Han Chinese in Beijing, China) and YRI (Yoruba in Ibadan, Nigeria). Among these peaks, the segment chr4:70389000–70392000 reaches the highest *π* (see Supplementary Fig. 4a) and Tajima’s *D* (see Supplementary Fig. 4b) simultaneously. For this region, *π* is ~ 0.00918, ~ 0.00627 and 0.00826, and Tajima’s *D* is ~ 4.34, ~ 2.03 and ~ 3.32 for CEU, CHB and YRI, respectively. Other 1000G populations showed a similar pattern (results not shown), thus indicating that the mutation pattern within this region is rather unusual.

We also utilized the software GWAVA (https://www.sanger.ac.uk/sanger/StatGen_Gwava) to predict the functional region for non-coding sequences. This software annotates the genome region by using multiple kinds of data, including chromatin structure, conservation and epigenetic modification. As shown in Supplementary Fig. 4c, multiple variations with high scores are observed in segment chr4:70389000–70392000. All these facts indicate that this segment should be the functional region.

### Identification of functional region

To verify the mutation pattern, we selected 25 unrelated East Asian and resequenced this segment. As shown in Supplementary Fig. 5, 46 SNPs are observed. Among them, 42 are within the core haplotype (see Supplementary Table 2), which is consistent with the pattern in 1000G data.

**Figure 4:**
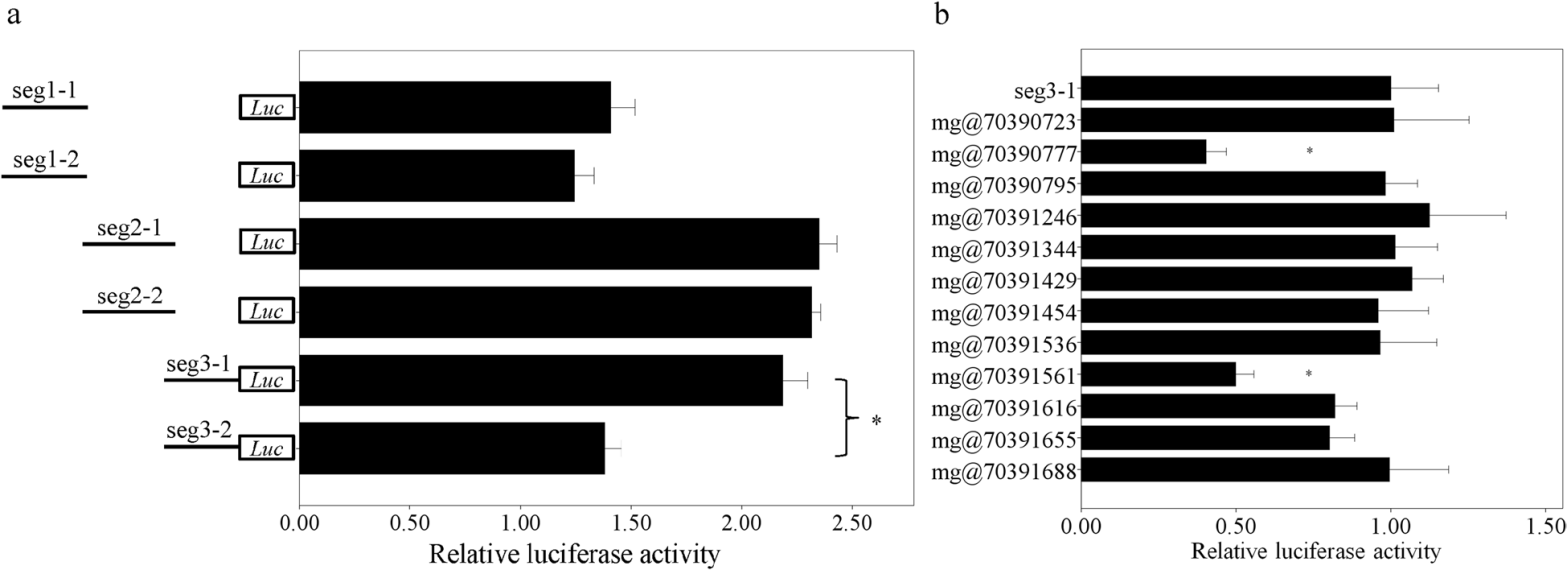
Function of the segments in chr4:70389000-70392000 (**a**) and mutations in seg3 (**b**). Each bar represents one plasmid. All data is displayed as mean ± standard deviation (SD). The *x* axis and * represent relative luciferase activity and* P* < 10^−6^, respectively. In part a, the lines in left indicate the relative location of the three segments. “Seg” is the abbreviation for “segment”. “−1” and “−2” represent core haplotype 1 and 2, respectively. The data is normalized to the read of empty vector (pGL3-promoter). For part b, the above plasmid (seg3-1) is the original construct while the below ones are from mutagenesis. Mg and the number after @ represent mutagenesis and SNP position in chr4, respectively. The data is normalized by the result of original plasmid (seg3-1).

To narrow the location of the functional element, we further separated the region into three overlapping segments (designated as seg 1-3) by primer availability, amplified both haplotypes, inserted them into pGL3-promoter vector and compared the luciferase activity. For each plasmid pair, no other mutations exist besides the ones in core haplotypes. The segments 1, 2 and 3 contain 5, 26 and 12 SNPs in core haplotypes, respectively (see Supplementary Table 5; rs6836432 appears in both segment 2 and 3). As shown in Fig. 4a, no significant enhancer activity difference is observed between two haplotypes for segments 1 and 2 (*P* = 0.49 and 0.55, respectively; n = 6 for each group). In contrast, for segment 3 (corresponding to chr4: 70390572–70391811; see Supplementary Table 5), the luciferase activity of haplotype 1 is ~ 58.5% higher than that of haplotype 2 (*P* = 2.8 × 10^–7^; n = 6 for each group), thus indicating that this region is functional in liver tissue and at least one of the 12 mutations in this segment can influence enhancer activity.

### Identification of the functional variations

There are 12 SNPs between the two core haplotypes in segment 3. To identify the functional one(s), we generated multiple plasmids and transfected them. Each plasmid is with only one nucleotide difference from plasmid seg3-1. As shown in Fig. 4b, the luciferase activity decrease ~ 59.7% and ~ 50.2% when mutating at 70390777 (rs66862535;* P* = 8.1 × 10^–9^; n = 6 for each group) and 70391561 (rs68096061; *P* = 3.8 × 10^–8^; n = 6 for each group), respectively. In contrast, other mutations fail to alter luciferase activity (all *P* > 0.05; n = 6 for each group), thus indicating that only rs66862535 and rs68096061 are functional in liver cell.

### Regulatory target of the enhancer

**Figure 5:**
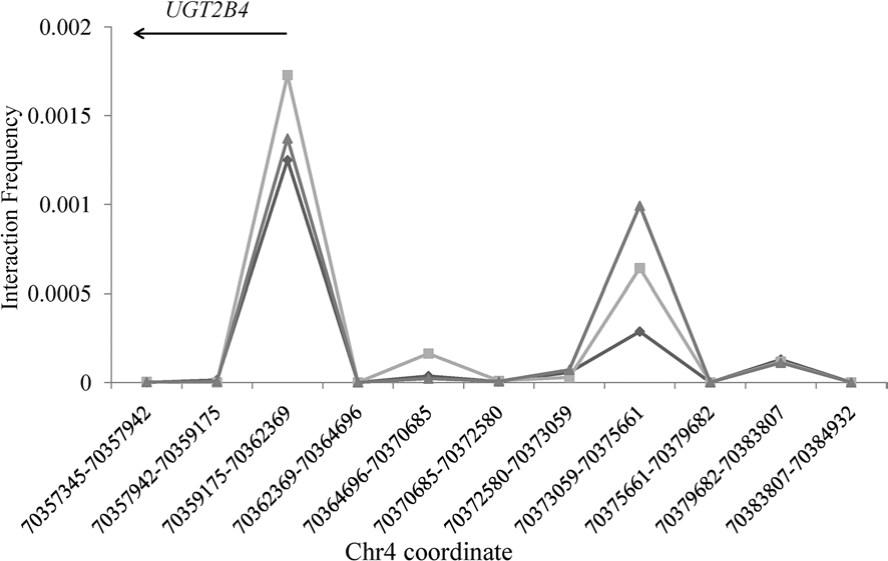
Interaction frequency between the enhancer and other segments in chr4. Each point in *x* axis designates one restrictive fragment and its start and end are shown below. The *y* axis represents 3C-PCR amount normalized to the BAC clone. Each line indicates one repeat. The above horizontal arrow indicates the illustrative location and transcript orientation of *UGT2B4*.

Rs66862535 and rs68096061 are within the non-coding region but not locating at the promoter region of any known genes. Moreover, the search in ENCODE project (https://www.encodeproject.org/) indicates that there are apparent H3K4me1 and H3K27Ac histone modifications, two common signals for active enhancer^29^, around this two SNPs in HepG2 cell line (see Supplementary Fig. 6). Therefore, we hypothesized that these two SNPs might be within enhancer region and could regulate gene expression. Since most enhancer could only regulate nearby genes, we hypothesized that the regulation target of this putative enhancer should be *UGT2B4*. To substantiate this issue, we utilized chromosome conformation capture (3C) to investigate whether there is interaction between this putative enhancer and *UGT2B4* promoter (in ~ 29.9 kb distance). Unidirectional primers were set to anchor *UGT2B4* promoter and 10 random selected regions while the constant primer was designed to bind the segment containing the putative enhancer. As shown in Fig. 5, a high ligation frequency is observed between *UGT2B4* promoter region and this enhancer. If we roughly used one-sample *t*-test to compare the ligation frequency difference between *UGT2B4* promoter and other 10 regions, a significant deviation (*P* = 0.004; n = 3 for each group) can be obtained, thus indicating that *UGT2B4* is the regulation target of this enhancer.

### Association between core haplotype and *UGT2B4* expression

If the two SNPs could indeed influence *UGT2B4* expression, these SNPs should be an eQTL for this gene. To verify this issue, RNA-seq data from YRI lymphoblastoid cell lines (LCL), a well-established model for eQTL analysis, were obtained from literature^30^ and *UGT2B4* expression was calculated. Since the genotype is not available in HapMap for the two functional SNPs, one tag SNP in HGDP, rs11723463 (*r*^*2*^ = 1 with both rs66862535 and rs68096061 in YRI), was chosen to represent the core haplotype. In this site, G is from haplotype 1 while A is from haplotype 2 (see Supplementary Table 2^2^. As shown in Supplementary Fig. 7, a significant correlation is observed between rs11723463 genotype and *UGT2B4* expression (*r*^*2*^ = 0.235, *P* = 0.004, n = 161 in total; see Supplementary Table 6 or detailed results). Moreover, G allele is associated with a higher expression (see Supplementary Fig. 7), which is consistent with our luciferase result.

We further verified this correlation in GTEx database (https://gtexportal.org/). As shown in Supplementary Fig. 8, rs11723463 genotype is associated with *UGT2B4* expression in as many as seven kind of tissues, including lung (*P* = 1.50 × 10^–101^), heart-atrial appendage (*P* = 6.20 × 10^–86^), heart-left ventricle (*P* = 1.90 × 10^–46^), adipose-visceral (omentum, *P* = 1.30 × 10^–40^), adipose-subcutaneous (*P* = 8.20 × 10^–18^), breast mammary (*P* = 4.00 × 10^–10^) and artery coronary (*P* = 3.00 × 10^–08^). The trend is also in agreement with our luciferase result. This association is further strengthened by the search in Open Target Genetics (https://genetics.opentargets.org/)*.*

We also validated this association in expression data from TCGA (The Cancer Genome Atlas) project by PancanQTL (http://gong_lab.hzau.edu.cn/PancanQTL/). As shown in Supplementary Fig. 9, rs11723463 is associated with *UGT2B4* expression in LUSC (lung squamous cell carcinoma; *P* = 7.54 × 10^–18^), LUAD (lung adenocarcinoma; *P* = 5.0 × 10^–15^) and BRCA (breast invasive carcinoma; *P* = 1.5 × 10^–7^) tissues. All these facts indicate that this enhancer effects in multiple tissues.

There are also another two genes, *UGT2A1* and *SULT1B1* (sulfotransferase family 1B member 1), near the enhancer region. Due to the relatively long distance, our 3C assay failed to include them. Therefore, no conclusion could be made for this issue. However, no significant correlations are observed between the genotype of this locus and the expression of these two genes in our LCL analysis (*P* = 0.13 and 0.77, n = 161 in total) or database search. These results suggest that these two genes should not be the regulation target of this enhancer. The associations between genotype of this locus and another two UGT2B family members under balancing selection, *UGT2B28* and *UGT2B11 *^23^, were also investigated. Similarly, no significant associations are obtained for these two genes in our LCL cohort (*P* = 0.18 and 0.47, n = 161 in total) and database inquiry, which verifies the conclusion that these three loci are independent.

### Related transcription factor (TF) binding rs66862535 and rs68096061

Since rs66862535 and rs68096061 are locating in the non-coding region, it was reasonable to hypothesize that these two SNPs might be within TF binding site and the mutation could influence TF binding. Online program Match (http://www.gene-regulation.com/cgi-bin/pub/programs/match/bin/match.cgi) was utilized to predict the potential TF. It was suggested that G of rs66862535 (in haplotype 2) and rs68096061 (in haplotype 1) could interact with TF POU2F1 (POU class 2 homeobox 1). In contrast, the corresponding allele of these two SNPs could abolish the binding site. To substantiate this hypothesis, we performed chromatin immunoprecipitation (ChIP) using related antibody and quantified the enrichment. As shown in Supplementary Fig. 10, the anti-POU2F1 immunoprecipitated chromatin samples are significantly enriched for the region surrounding rs66862535 (*P* = 0.002; n = 3 for each group) and rs68096061 (*P* = 0.003; n = 3 for each group) compared with IgG, thus confirming the interaction between POU2F1 and this region in liver tissue.

### TF binding affinity difference between rs66862535 and rs68096061 alleles

To illuminate the interaction efficiency between two alleles at rs66862535 and rs68096061, electrophoretic mobility shift assay (EMSA) was performed based on biotin-labeled probes. As shown in Supplementary Fig. 1, the two alleles of rs66862535 and rs68096061 present obvious different affinity with nuclear proteins from HepG2 cell line and G allele at both sites are with a higher combining capability, which is consistent with our bioinformatics prediction. Moreover, the interaction between nuclear protein and probes can be abolished by competitor (unlabeled probe), which indicates that the interaction is specific. It is also interesting to observe that G allele of rs66862535 can interact with POU2F1 in higher efficiency but present lower enhancer activity (see Fig. 4b). This phenomenon might be interpreted by the hypothesis that POU2F1 could interact with other *trans*-regulatory elements and play a negative regulation in surrounding region.

## Discussion

In current study, we describe the two haplotypes upstream human *UGT2B4* with deep divergence by analyzing 1000G data. Through comparing with known archaic genomes, it is suggested that one haplotype is lost in Neanderthal and Denisovan evolution. Through population genetics prediction and functional genomics approaches, the functional variations are identified and the mechanism is disclosed.

In *UGT2B4* upstream region, the LD block extends ~ 70.3 and ~ 54.9 kb in non-African and African population, respectively, which is much longer than expected. Indeed, if we set the original time of these two core haplotypes, regional recombination rate and the generation time as 1.54 million years, 0.62 cM/Mb^31^ and 25 years, it can be speculated by a published formula^32^ that the DNA in this region should be broken into segments with expected length ~ 2.62 kb. The reason for this is still unknown. There might be some *trans*-elements attaching this region and further inhibiting recombination.

In phylogeny, a much longer branch can be observed for haplotype 2 lineages (see Fig. 2), which is corresponding to a much more recent expansion. Considering this and the genotype of chimpanzee, it can be deduced that haplotype 2 is the derived allele and its frequency increases after human—Neanderthal/Denisovan divergence. It is also interesting to observe that Neanderthal and Denisovan inherit the minor haplotype but lose the common one due to some unknown reasons. One possibility is that this genotype can provide some selection advantages for the common ancestor of Neanderthal and Denisovan.

Our results substantiate a lower *UGT2B4* expression for haplotype 2 carriers. Although UGT2B4 is not the enzyme with highest activity on steroid hormone^33^, its extremely high protein amount in human liver makes it an important factor for steroid hormone level. Although the association between *UGT2B4* expression and steroid hormone level has not been reported, it has been proposed that *UGT2B17* and *UGT2B15*, another two members in this family, present a negative correlation between gene expression and steroid hormone level or response^34–37^. Therefore, the haplotype 2 carriers have a high possibility to be with higher steroid hormone level.

Our previous study suggested an increased breast cancer risk for haplotype 2 carriers^4^, which is verified by search in Open Target Genetics database (*P* = 0.023; tagged by rs11723463). Considering the function of this haplotype, the association should result from a lower *UGT2B4* expression and further a higher steroid hormone level. To verify this issue, we used UALCAN (http://ualcan.path.uab.edu/index.html) to examine *UGT2B4* expression between breast cancer patients and controls from TCGA project. As shown in Supplementary Fig. 12, *UGT2B4* expression in normal breast tissue is significant higher than that in BRCA (*P* = 3.8 × 10^–3^). These results verify the previous proposal that *UGT2B4* is a tumor suppressor gene for breast cancer^16,17,38^.

All Neanderthal and Denisovan individuals are homozygous of haplotype 2, and thus should be with a relatively lower *UGT2B4* expression and further higher steroid hormone level than modern human. However, we caution about this conclusion since steroid hormone level is influenced not only by glucuronidation speed but also by producing. Since most Neanderthals die before 40 years old^39^, an age with very low breast cancer morbidity, the incidence of this disease in Neanderthal should be rather low and not influenced by the genotype of this locus. Moreover, Neanderthal presents more robust bones than modern human^40^, which might, at least partially, be caused by the higher steroid hormone level. Indeed, it has been widely accepted that a higher level of steroid hormone, including both estrogen and androgen, can induce a relatively higher bone mineral density (BMD)^41–43^. In this issue, it is interesting to observe that this haplotype is significantly associated with BMD in a genome-wide association study (*P* = 0.04; tagged by rs11723463)^44^, which supports our hypothesis from another side.

The widespread and high frequency hint the importance of haplotype 2 for human. Although haplotype 2 is associated with increased breast cancer risk, this disease should not be the phenotype accounting for balancing selection since breast cancer is harmful for individual and usually occurs > 50 years old. We hypothesize that the selection advantage of heterozygous individual might be maintaining a medium level of steroid hormone, especially sex hormone in reproductive system. Indeed, sex hormones, including estrogen, androgen and progestin, play an extremely important role in reproductive system function maintaining, menstrual cycle and pregnancy, which are essential for our species surviving. Any deviation from proper sex hormone level will cause a range of symptoms. For example, low progestin level would induce bleeding in early pregnancy, abortion or even infertility. In contrast, abnormally high estrogen level in female could induce menstrual chaos while high androgen level in male could lead to testicles shrink. All deviations from proper level of steroid hormone are harmful for human reproduction. Therefore, the heterozygous individuals at this locus might have a better chance to keep a proper sex hormone level and further present selection advantage in reproduction, which deserves further investigation. In this regard, it is interesting to observe that balancing selection is also observed in other members in this gene family, including *UGT2B17*^45^, *UGT2B28* and *UGT2A1*^23^. Moreover, a search in Open Targets Genetics database indicates that this haplotype (tagged by rs11723463) is significantly associated with multiple diseases in reproductive system, including primary ovarian failure (*P* = 0.034), postpartum depression (*P* = 0.017), haemorrhage in early pregnancy (*P* = 0.024) and spontaneous (*P* = 0.013) and other (*P* = 0.034) abortion (most results from FinnGen [https://www.finngen.fi]), which partially supports our hypothesis. We confess that the *P* values for these phenotypes fail to reach genome-wide significance, which might be due to the genetic models. Indeed, in most studies, additive, dominant and recessive models are used to detect the association. However, in balancing selection scenario, heterozygous individuals are expected to present more advantageous phenotype than homozygous ones. This phenomenon is quite different with these three regular models and might attenuate or even mask the association between genotype and phenotype. In addition to this, it is also possible that the balancing selection might be attributed to different phenotypes between genders. Indeed, male and female show essential dissimilarity in reproductive system.

Our results substantiate the regulation of this locus under balancing selection on *UGT2B4* expression. SV esv3600896 contains entire *UGT2B28* gene and is supposed to be under balancing selection^23^. Therefore, the association between esv3600896 genotype and *UGT2B28* or *UGT2B11* expression was also investigated in abovementioned LCL cohort. As shown in Supplementary Fig. 13, the deletion can induce a significant decrease in *UGT2B28* expression (*r*^*2*^ = 0.45, *P* < 10^–6^, n = 161 in total). In contrast, it seems that the deletion can cause a higher *UGT2B11* expression but fail to reach statistical significance (*r*^*2*^ = 0.11, *P* = 0.17, n = 161 in total), which might be due to some outliers with extremely low expression. When these outliers are removed from analysis, a significant association can be obtained (*r*^*2*^ = 0.20, *P* = 0.022, n = 150 in total), which is consistent with previous report^23^.

## Methods

### 1000G and archaic hominin sequence analysis

The genotype (PED format) for *UGT2B4* region was downloaded from 1000G website (http://www.internationalgenome.org/). The LD pattern was determined by PLINK (https://zzz.bwh.harvard.edu/plink/) with threshold *r*^*2*^ ≥ 0.8.

The genome sequence for the corresponding region from three Neanderthal individuals, Altai^22^, Chagyrskaya^26^ and Vindija^27^, and Denisovan^28^ were downloaded from Max Planck Institute for Evolutionary Anthropology (http://cdna.eva.mpg.de/). For heterozygous positions, standard degenerate base was used in sequence. After alignment by ClustalW (http://www.clustal.org/), the phylogeny was reconstructed by maximum likelihood method through utilizing MGEA X (https://megasoftware.net/). To estimate the confidence of each branch, 100 bootstraps were included in phylogeny reconstruction.

### Population genetics analysis and genome annotation

DnaSP 6 (http://www.ub.edu/dnasp/) was utilized to calculate population genetics indices, such as segregating sites (*S*), nucleotide diversity (*π*), Watterson’s estimator of the population mutation rate parameter (*θ*_*w*_) and Tajima’s *D*. The expected Tajima’s *D* distribution was simulated by ms (https://home.uchicago.edu/~rhudson1/source/mksamples.html). The sliding window for these population genetics indices was calculated by Slider (http://genapps.uchicago.edu/labweb/index.html) with window size 1.5 kb and increment 100 bp.

### Resequencing

The genome region around chr4:70,389,000–70,392,000 was amplified by the primers in Supplementary Table 7. After exonuclease I and Shrimp Alkaline Phosphatase (Takara, Dalian, China) treatment, resequencing was performed by using PCR and internal primers in Supplementary Table 7. Polymorphisms were evaluated by PolyPhred (https://droog.gs.washington.edu/polyphred/) and verified by visual examination. Visual genotype was generated by Genome Variation Server (http://gvs.gs.washington.edu/GVS/).

### Plasmid construction, cell culture and transfection

*UGT2B4* promoter region was amplified by primer pair CAGTCGGTACCTCAGCTCCTTGTGGGGTCCT and CAGTCGCTAGCCCTGATGCAATGCAATGCTT from individuals with specific haplotypes. After *Kpn*I and *Nhe*I (Thermo Fisher Scientific, Grand Island, NY) digestion, PCR product was fused with pGL3-basic vector (Promega, Madison, WI).

The region chr4:70389000–70392000 was separated into three segments (seg1-3) and amplified by primer in Supplementary Table 5 from individuals with specific haplotypes. After *Xho*I and *Kpn*I (Thermo Fisher Scientific) digestion, the PCR product was inserted into pGL3-promoter vector (Promega).

Phusion High-Fidelity DNA Polymerase (Thermo Fisher Scientific) was used in PCR to avoid artificial mutations. Before transfection, all plasmids were sequenced to eliminate the possibility of any PCR errors and validate the haplotype orientation.

Human liver cancer cell line HepG2 was purchased from conservation genetics CAS Kunming cell bank (http://www.kmcellbank.com/) and maintained in Dulbecco's modified Eagle's medium (high glucose, HyClone, Logan, UT) with 10% fetal bovine serum (Thermo Fisher Scientific) in 5% CO_2_ at 37 °C. 10^5^ cells were seeded and cultured for 24 h. 475 ng plasmid was transfected through using Lipofectamine 2000 (Thermo Fisher Scientific) according to the manufacturer’s protocol. After additional 36 h culture, cells were collected and lysed. The luciferase activity was determined by using Dual-Luciferase Reporter Assay System (Promega). 25 ng pRL-TK (Promega) was transfected simultaneously as an internal control. The relative luciferase expression was determined by the ratio between firefly and *Renilla* amount. Six replicates were included for each plasmid.

### Mutagenesis and transfection

For the 12 SNPs in seg3, the corresponding allele in another haplotype was generated by mutagenesis based on plasmid seg3-1. The mutagenesis was performed by Q5 Site-Directed Mutagenesis Kit (NEB) and primers in Supplementary Table 8. The following transfection and luciferase reading was performed as abovementioned.

### 3C

Interactions between enhancer and nearby gene promoter were determined by 3C and quantified by quantitative real-time PCR (qPCR). In summary, ~ 10^8^ HepG2 cells were cross-linked by formaldehyde and lysed, and the chromatin was digested by *Hind*III enzyme (NEB). After ligation with T4 DNA ligase (NEB), DNA was purified.

Along with the chromatin, the BAC RP11-121J8 was ordered from BACPAC Resources Center (http://bacpac.chori.org/), cultured, isolated by Large-Construct Kit (Qiagen, Valencia, CA), digested with the same enzyme and ligated as control.

The relative amount of 3C product was quantified by qPCR with iQ SYBR green (Bio-Rad, Hercules, CA) and unidirectional primers in Supplementary Table 9. The relative enrichment for HepG2 chromatin was determined by 2^-ΔΔCt^, in which ΔΔCt is the threshold cycle difference between BAC and chromatin. Triplicates were performed for each 3C-qPCR. All 3C products were sequenced for validation.

### RNA-seq data analysis

The RNA-seq data (sra format) for LCL^30^ was obtained from SRA database (https://www.ncbi.nlm.nih.gov/sra/) and converted to fastq format by SRA toolkit (https://github.com/ncbi/sra-tools). After alignment with *UGT2B4* mRNA sequence by bowtie2 (http://bowtie-bio.sourceforge.net/bowtie2/index.shtml), the expression was calculated by eXpress (https://pachterlab.github.io/eXpress/) with default parameter and reported in FPKM (Fragments Per Kilobase of transcript per Million fragments mapped) unit. The genotypes for LCLs were obtained from HapMap project (https://www.genome.gov/10001688/international-hapmap-project). Linear regression was performed between LCL genotype and *UGT2B4* expression in SPSS 20.0 (IBM, Armonk, NY).

### ChIP

ChIP was carried out by EZ ChIP Kit (Millipore, Burlington, MA). Briefly, crosslink was performed in ~ 10^7^ cells with formaldehyde and terminated by glycine. After washing, cells were collected, lysed and broken into small fragments (~ 400–800 bp) by sonication. The chromatin/TF complex was diluted, precleared, captured by mouse anti-POU2F1 antibody or normal mouse IgG (Santa Cruz Biotechnology, Santa Cruz, CA), respectively, and precipitated by protein A beads. After washing, the chromatin/TF complex was resuspended and the crosslink were reversed. After protein digestion, DNA was purified and quantified by qPCR with primers in Supplementary Table 10.

### EMSA

The probes for both alleles of rs68096061 and rs66862535 were displayed in Supplementary Table 11 and labeled by EMSA probe biotin labeling kit (Beyotime, Shanghai, China). Nuclear extracts were prepared and quantified using Nuclear and cytoplasmic protein extraction kit (Beyotime). The nuclear protein (5 µg) was incubated with biotin-labeled probes (10fmol). The probe-protein complexes were separated on non-denaturing polyacrylamide gel (6%) and transferred to positively charged nylon membranes (Beyotime). After blocking and incubating with streptavidin-HRP (horseradish peroxidase) conjugate, the membrane was visualized by chemiluminescent EMSA kit (Beyotime) in ECL chemiluminescence (Millipore) according to the manufacturer’s protocol.

## Supplementary Information

Supplementary Information 1.

Supplementary Information 2.

Supplementary Information 3.

## Data Availability

All data generated or analyzed during this study are included in this published article (and its Supplementary Information files).
