# Supplementary figures and images for: Distilling functional variations for human UGT2B4 upstream region based on selection signals and implications for phenotypes of Neanderthal and Denisovan
Source: Sci Rep. 2023 Feb 23;13:3134. doi: 10.1038/s41598-023-29682-x (PMC9950360; doi:10.1038/s41598-023-29682-x)

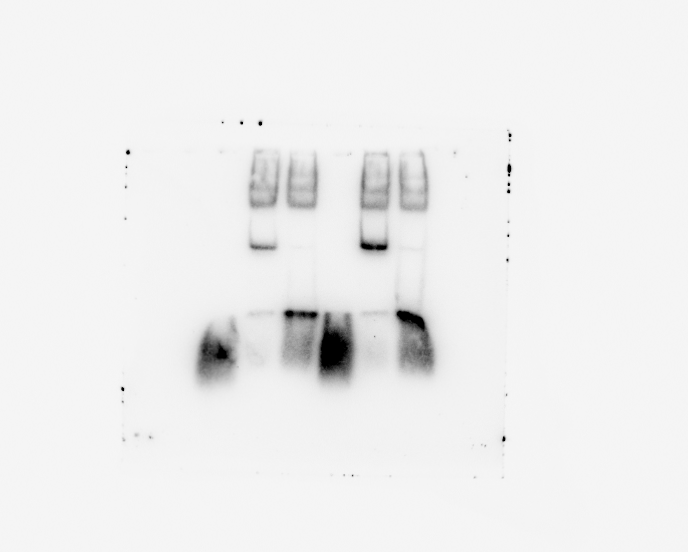

Supplement: Supplementary file 1 — Supplementary Information 1. [file 41598_2023_29682_MOESM1_ESM.tif]

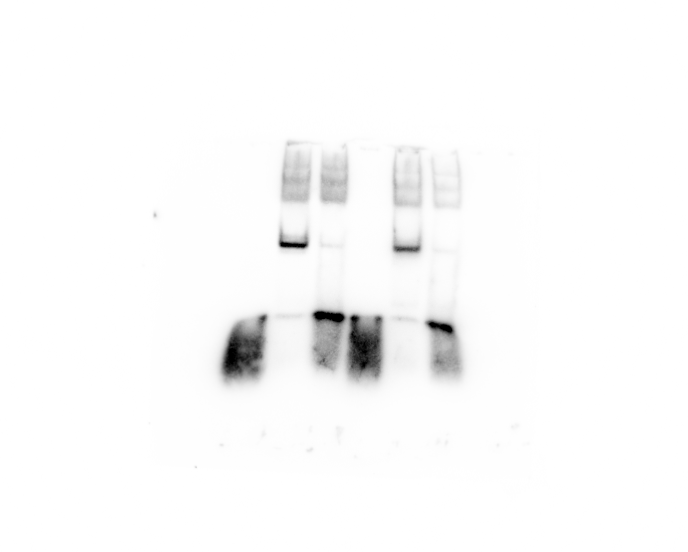

Supplement: Supplementary file 2 — Supplementary Information 2. [file 41598_2023_29682_MOESM2_ESM.tif]
