# Supplementary material for: Distilling functional variations for human UGT2B4 upstream region based on selection signals and implications for phenotypes of Neanderthal and Denisovan
Source: Sci Rep. 2023 Feb 23;13:3134. doi: 10.1038/s41598-023-29682-x (PMC9950360; doi:10.1038/s41598-023-29682-x)

Supplementary Table 1. Genetic variations in the linkage disequilibrium block for different populations.

| Pop | frequency | SNP# | SNP list (position in chr4) |
| --- | --- | --- | --- |
| ACB (African Caribbeans in Barbados) | 40% | 399 | 70376705 70376708 70376963 70377360 70377361 70377394 70377396 70377402 70377524 70377642 70378555 70379374 70381259 70382444 70382578 70382579 70383965 70383991 70385252 70385552 70385588 70385589 70386623 70386791 70387066 70387375 70388363 70388497 70388537 70388610 70388617 70388623 70388633 70388704 70388958 70389194 70389266 70389349 70389486 70389823 70389868 70389916 70389917 70389934 70389935 70390000 70390024 70390051 70390069 70390098 70390135 70390151 70390175 70390221 70390272 70390273 70390281 70390302 70390358 70390449 70390494 70390528 70390554 70390723 70390777 70390795 70391246 70391344 70391429 70391454 70391536 70391561 70391616 70391655 70391688 70391986 70392053 70392062 70392874 70392967 70393660 70393665 70393680 70393780 70394240 70394493 70394500 70394564 70394991 70395188 70395338 70395596 70395767 70395803 70395821 70396060 70396093 70396137 70396352 70396415 70396562 70396681 70396710 70397007 70397202 70397236 70397237 70397266 70397274 70397323 70397408 70397424 70397507 70397849 70397899 70398467 70398606 70398769 70398857 70399017 70399573 70399615 70399774 70399877 70399972 70400027 70400339 70400391 70400450 70400462 70400631 70400843 70400884 70400905 70401025 70401093 70401103 70401279 70401513 70401731 70401751 70401818 70401824 70401832 70401931 70402005 70402042 70402181 70402246 70402312 70402805 70402954 70402958 70403070 70403427 70403460 70403503 70403702 70403764 70403815 70404001 70404130 70404144 70404150 70404196 70404759 70404889 70404918 70404922 70405360 70405404 70405545 70405551 70405916 70405973 70406203 70406423 70406450 70406601 70406851 70406908 70406918 70406939 70406964 70406966 70407039 70407054 70407123 70407232 70407359 70407643 70407762 70407772 70407784 70407823 70407993 70408190 70408309 70408436 70408894 70408922 70408931 70409574 70409752 70409818 70409826 70410184 70410273 70411070 70411081 70411132 70411395 70412096 70412356 70412437 70412606 70412912 70413031 70413032 70413192 70413508 70413777 70413804 70413905 70414029 70414599 70414640 70414689 70414796 70414930 70414931 70415364 70415522 70415549 70415655 70415744 70415787 70415812 70415877 70415904 70415954 70416013 70416155 70416321 70416464 70416480 70416486 70416526 70416542 70416600 70416785 70417188 70417215 70417286 70417394 70417495 70417646 70417733 70418085 70418179 70418277 70418279 70418312 70418313 70418395 70418471 70418493 70418650 70418723 70418849 70419361 70419420 70419458 70419473 70419567 70419588 70419884 70419913 70420426 70420487 70420547 70420639 70420812 70420888 70421022 70421080 70421227 70421307 70421369 70421601 70421618 70421675 70421689 70421771 70421938 70421986 70422100 70422141 70422199 70422296 70422344 70422704 70422727 70422745 70422851 70422923 70423008 70423014 70423051 70423236 70423325 70423580 70423633 70424215 70424226 70424811 70425058 70425151 70425165 70425203 70425220 70425296 70425337 70425430 70425434 70425826 70426147 70426383 70426388 70426795 70426866 70427362 70427571 70427607 70427623 70427653 70427888 70427947 70428039 70428058 70428064 70428558 70428840 70428874 70429211 70429421 70429565 70429748 70429812 70429896 70430041 70430155 70430536 70430879 70430909 70430979 70431063 70431070 70431130 70431261 70431410 70431505 70431589 70379727(i)^a^ 70389268(i) 70389833(i) 70390102(i) 70397714(i) 70397823(i) 70400235(i) 70400524(i) 70401303(i) 70401741(i) 70403954(i) 70403982(i) 70403989(i) 70404913(i) 70406212(i) 70406234(i) 70406255(i) 70407786(i) 70411912(i) 70413952(i) 70413961(i) 70414468(i) 70417543(i) 70419034(i) 70419720(i) 70421898(i) 70425358(i) 70427192(i) 70427272(i) 70428224(i) 70428533(i) 70430184(i) 70430233(i) 70430545(i) 70430557(i) 70431614(i) |
| ASW (Americans of African Ancestry in SW USA) | 33% | 400 | 70376705 70376708 70376963 70377360 70377361 70377394 70377396 70377402 70377524 70377642 70378555 70379374 70381259 70382444 70382578 70382579 70383965 70383991 70385252 70385552 70385588 70385589 70386623 70386791 70387066 70387375 70388363 70388497 70388537 70388610 70388617 70388623 70388633 70388704 70388958 70389194 70389266 70389349 70389486 70389823 70389868 70389916 70389917 70389934 70389935 70390000 70390024 70390051 70390069 70390098 70390135 70390151 70390175 70390221 70390272 70390273 70390281 70390302 70390358 70390449 70390494 70390528 70390554 70390723 70390777 70390795 70391246 70391344 70391429 70391454 70391536 70391561 70391616 70391655 70391688 70391986 70392053 70392062 70392874 70392967 70393660 70393665 70393680 70393780 70394240 70394493 70394500 70394564 70394991 70395188 70395338 70395596 70395767 70395803 70395821 70396060 70396093 70396137 70396352 70396415 70396562 70396681 70396710 70397007 70397202 70397236 70397237 70397266 70397274 70397323 70397408 70397424 70397507 70397849 70397899 70398467 70398606 70398769 70398857 70399017 70399573 70399615 70399706 70399774 70399877 70399972 70400027 70400339 70400391 70400450 70400462 70400631 70400843 70400884 70400905 70401025 70401093 70401103 70401279 70401513 70401731 70401751 70401818 70401824 70401832 70401931 70402005 70402042 70402181 70402246 70402312 70402805 70402954 70402958 70403070 70403427 70403460 70403503 70403702 70403764 70403815 70404001 70404130 70404144 70404150 70404196 70404759 70404889 70404918 70404922 70405360 70405404 70405545 70405551 70405916 70405973 70406203 70406423 70406450 70406601 70406851 70406908 70406918 70406939 70406964 70406966 70407039 70407054 70407123 70407232 70407359 70407643 70407762 70407772 70407784 70407823 70407993 70408190 70408309 70408436 70408894 70408922 70408931 70409574 70409752 70409818 70409826 70410184 70410273 70411070 70411081 70411132 70411395 70412096 70412356 70412437 70412606 70412912 70413031 70413032 70413192 70413508 70413777 70413804 70413905 70414029 70414599 70414640 70414689 70414796 70414930 70414931 70415364 70415522 70415549 70415655 70415744 70415787 70415812 70415877 70415904 70415954 70416013 70416155 70416321 70416464 70416480 70416486 70416526 70416542 70416600 70416785 70417188 70417215 70417286 70417394 70417495 70417646 70417733 70418085 70418179 70418277 70418279 70418312 70418313 70418395 70418471 70418493 70418650 70418723 70418849 70419361 70419420 70419458 70419473 70419567 70419588 70419884 70419913 70420426 70420487 70420547 70420639 70420812 70420888 70421022 70421080 70421227 70421307 70421369 70421601 70421618 70421675 70421689 70421771 70421938 70421986 70422100 70422141 70422199 70422296 70422344 70422704 70422727 70422745 70422851 70422923 70423008 70423014 70423051 70423236 70423325 70423580 70423633 70424215 70424226 70424811 70425058 70425151 70425165 70425203 70425220 70425296 70425337 70425430 70425434 70425826 70426147 70426383 70426388 70426795 70426866 70427362 70427571 70427607 70427623 70427653 70427888 70427947 70428039 70428058 70428064 70428558 70428840 70428874 70429211 70429421 70429565 70429748 70429812 70429896 70430041 70430155 70430536 70430879 70430909 70430979 70431063 70431070 70431130 70431261 70431410 70431505 70431589 70379727(i) 70389268(i) 70389833(i) 70390102(i) 70397714(i) 70397823(i) 70400235(i) 70400524(i) 70401303(i) 70401741(i) 70403954(i) 70403982(i) 70403989(i) 70404913(i) 70406212(i) 70406234(i) 70406255(i) 70407786(i) 70411912(i) 70413952(i) 70413961(i) 70414468(i) 70417543(i) 70419034(i) 70419720(i) 70421898(i) 70425358(i) 70427192(i) 70427272(i) 70428224(i) 70428533(i) 70430184(i) 70430233(i) 70430545(i) 70430557(i) 70431614(i) |
| BEB (Bengali from Bangladesh) | 30% | 455 | 70362123 70367399 70372905 70372955 70374584 70374588 70375691 70376112 70376963 70377360 70377361 70377394 70377396 70377402 70377524 70377642 70378396 70378440 70378555 70379374 70379682 70380660 70380675 70381259 70381351 70382444 70382578 70382579 70383322 70383913 70383965 70383991 70384123 70384324 70385252 70385552 70385588 70385589 70386429 70386623 70386791 70387066 70387375 70388363 70388497 70388537 70388610 70388617 70388623 70388633 70388646 70388704 70388958 70389194 70389266 70389349 70389486 70389716 70389823 70389868 70389916 70389917 70389934 70389935 70390000 70390024 70390051 70390069 70390098 70390135 70390151 70390175 70390221 70390272 70390273 70390281 70390302 70390358 70390449 70390494 70390525 70390528 70390554 70390723 70390777 70390795 70391246 70391344 70391429 70391454 70391536 70391561 70391616 70391655 70391688 70391888 70391986 70392053 70392062 70392874 70392967 70393590 70393660 70393665 70393680 70393780 70394240 70394493 70394500 70394564 70394991 70395188 70395338 70395353 70395596 70395767 70395803 70395821 70395945 70396060 70396093 70396137 70396352 70396415 70396562 70396681 70396710 70397007 70397202 70397236 70397237 70397266 70397274 70397323 70397408 70397424 70397507 70397849 70397899 70397977 70398467 70398606 70398769 70398783 70398857 70399017 70399573 70399615 70399643 70399706 70399774 70399802 70399877 70399972 70400027 70400339 70400391 70400439 70400450 70400462 70400631 70400843 70400884 70400905 70401025 70401093 70401103 70401279 70401513 70401731 70401751 70401818 70401824 70401832 70401931 70402005 70402042 70402181 70402246 70402312 70402805 70402954 70402958 70403070 70403427 70403460 70403503 70403702 70403764 70403815 70404001 70404130 70404144 70404150 70404196 70404759 70404889 70404918 70404922 70405360 70405404 70405545 70405551 70405916 70405973 70406203 70406423 70406450 70406575 70406601 70406851 70406908 70406918 70406939 70406957 70406964 70406966 70407039 70407054 70407123 70407232 70407314 70407359 70407643 70407762 70407772 70407784 70407823 70407993 70408190 70408244 70408309 70408416 70408436 70408894 70408922 70408931 70409525 70409534 70409574 70409688 70409752 70409818 70409826 70410184 70410273 70411070 70411081 70411132 70411395 70412096 70412356 70412437 70412606 70412912 70413031 70413032 70413192 70413508 70413777 70413804 70413905 70414029 70414599 70414640 70414689 70414796 70414930 70414931 70415364 70415522 70415549 70415655 70415744 70415787 70415812 70415877 70415904 70415954 70416013 70416121 70416155 70416321 70416464 70416480 70416486 70416526 70416542 70416600 70416785 70417188 70417215 70417286 70417394 70417495 70417646 70417733 70418085 70418179 70418277 70418279 70418312 70418313 70418395 70418471 70418493 70418650 70418723 70418849 70419361 70419420 70419458 70419473 70419567 70419588 70419884 70419913 70420001 70420426 70420487 70420547 70420639 70420812 70420888 70420900 70421022 70421080 70421227 70421307 70421369 70421486 70421528 70421601 70421618 70421675 70421689 70421771 70421938 70421986 70422100 70422141 70422199 70422296 70422344 70422704 70422727 70422745 70422851 70422890 70422923 70423008 70423014 70423051 70423236 70423325 70423580 70423633 70424215 70424226 70424811 70425058 70425151 70425165 70425203 70425220 70425296 70425337 70425430 70425434 70425826 70426071 70426147 70426383 70426388 70426795 70426866 70426973 70427362 70427554 70427571 70427607 70427623 70427653 70427888 70427947 70428039 70428058 70428064 70428558 70428599 70428840 70428874 70429211 70429409 70429421 70429565 70429748 70429812 70429896 70430041 70430155 70430536 70430879 70430909 70430979 70431063 70431070 70431130 70431261 70431410 70431476 70431505 70431589 70363728(i) 70370397(i) 70379727(i) 70389268(i) 70389833(i) 70390102(i) 70391979(i) 70397714(i) 70397823(i) 70400235(i) 70400524(i) 70401303(i) 70401741(i) 70402217(i) 70403954(i) 70403982(i) 70403989(i) 70404913(i) 70406212(i) 70406234(i) 70406255(i) 70407786(i) 70411912(i) 70413952(i) 70413961(i) 70414468(i) 70417253(i) 70417543(i) 70418956(i) 70419034(i) 70419720(i) 70421898(i) 70425358(i) 70427192(i) 70427272(i) 70428224(i) 70428533(i) 70430184(i) 70430233(i) 70430545(i) 70430557(i) 70431614(i) |
| CDX (Chinese Dai in Xishuangbanna, China) | 27% | 467 | 70361741 70362123 70366324 70366328 70366492 70367399 70372905 70372955 70374584 70374588 70375691 70376112 70376705 70376708 70376963 70377360 70377361 70377394 70377396 70377402 70377524 70377642 70378396 70378440 70378555 70379374 70379682 70380660 70380675 70381259 70381351 70382444 70382578 70382579 70383322 70383913 70383965 70383991 70384123 70384324 70385252 70385551 70385552 70385588 70385589 70386429 70386623 70386791 70387066 70387375 70388194 70388363 70388497 70388537 70388610 70388617 70388623 70388633 70388646 70388704 70388958 70389194 70389266 70389349 70389486 70389716 70389823 70389868 70389916 70389917 70389934 70389935 70390000 70390024 70390051 70390069 70390098 70390135 70390151 70390175 70390221 70390272 70390273 70390281 70390302 70390358 70390449 70390494 70390525 70390528 70390554 70390723 70390777 70390795 70391246 70391344 70391429 70391454 70391536 70391561 70391616 70391655 70391688 70391888 70391986 70392053 70392062 70392874 70392967 70393590 70393660 70393665 70393680 70393780 70394123 70394240 70394493 70394500 70394564 70394991 70395188 70395338 70395353 70395596 70395767 70395803 70395821 70395945 70396060 70396093 70396137 70396352 70396415 70396562 70396681 70396710 70397007 70397202 70397236 70397237 70397266 70397274 70397323 70397408 70397424 70397507 70397849 70397899 70397977 70398467 70398606 70398769 70398783 70398857 70399017 70399573 70399615 70399643 70399706 70399774 70399802 70399877 70399972 70400027 70400339 70400391 70400439 70400450 70400462 70400631 70400843 70400884 70400905 70401025 70401093 70401103 70401279 70401513 70401731 70401751 70401818 70401824 70401832 70401931 70402005 70402042 70402181 70402246 70402312 70402805 70402954 70402958 70403070 70403427 70403460 70403503 70403702 70403764 70403815 70404001 70404130 70404144 70404150 70404196 70404759 70404889 70404918 70404922 70405360 70405404 70405545 70405551 70405916 70405973 70406203 70406423 70406450 70406575 70406601 70406851 70406908 70406918 70406939 70406957 70406964 70406966 70407039 70407054 70407123 70407232 70407314 70407359 70407569 70407643 70407762 70407772 70407784 70407823 70407993 70408190 70408244 70408309 70408436 70408894 70408922 70408931 70409525 70409534 70409574 70409688 70409752 70409818 70409826 70410184 70410273 70411070 70411081 70411132 70411395 70412096 70412356 70412437 70412606 70412912 70413031 70413032 70413192 70413508 70413777 70413804 70413905 70414029 70414239 70414599 70414640 70414689 70414796 70414930 70414931 70415364 70415522 70415549 70415655 70415744 70415787 70415812 70415877 70415904 70415954 70416013 70416121 70416155 70416321 70416464 70416480 70416486 70416526 70416542 70416600 70416785 70417188 70417215 70417286 70417394 70417495 70417646 70417733 70418085 70418179 70418277 70418279 70418312 70418313 70418395 70418471 70418493 70418650 70418723 70418849 70419361 70419420 70419458 70419473 70419567 70419588 70419884 70419913 70420001 70420426 70420487 70420547 70420639 70420812 70420888 70420900 70421022 70421080 70421227 70421307 70421369 70421486 70421528 70421601 70421618 70421675 70421689 70421771 70421938 70421986 70422100 70422141 70422199 70422296 70422344 70422704 70422727 70422745 70422800 70422851 70422890 70422923 70423008 70423014 70423051 70423236 70423325 70423580 70423633 70424215 70424226 70424811 70425058 70425151 70425165 70425203 70425220 70425296 70425337 70425430 70425434 70425826 70426071 70426147 70426383 70426388 70426795 70426866 70426973 70427362 70427554 70427571 70427607 70427623 70427653 70427888 70427947 70428039 70428058 70428064 70428558 70428599 70428840 70428874 70429211 70429409 70429421 70429565 70429748 70429812 70429896 70430041 70430155 70430536 70430879 70430909 70430979 70431063 70431070 70431130 70431261 70431410 70431476 70431505 70431589 70432006 70363728(i) 70370397(i) 70379727(i) 70389268(i) 70389833(i) 70390102(i) 70391979(i) 70397714(i) 70397823(i) 70400235(i) 70400524(i) 70401303(i) 70401741(i) 70402217(i) 70403954(i) 70403982(i) 70403989(i) 70404913(i) 70406212(i) 70406234(i) 70406255(i) 70407786(i) 70411912(i) 70413952(i) 70413961(i) 70414468(i) 70417253(i) 70417543(i) 70418956(i) 70419034(i) 70419720(i) 70421898(i) 70425358(i) 70427192(i) 70427272(i) 70428224(i) 70428533(i) 70430184(i) 70430233(i) 70430545(i) 70430557(i) 70431614(i) |
| CEU (Utah Residents (CEPH) with Northern and Western European Ancestry) | 39% | 454 | 70361741 70362123 70367399 70372905 70372955 70374584 70374588 70375691 70376112 70376963 70377360 70377361 70377394 70377396 70377402 70377524 70377642 70378396 70378440 70378555 70379374 70379682 70380660 70380675 70381259 70381351 70382444 70382578 70382579 70383322 70383913 70383965 70383991 70384123 70384324 70385252 70385552 70385588 70385589 70386429 70386623 70386791 70387066 70387375 70388363 70388497 70388537 70388610 70388617 70388623 70388633 70388646 70388704 70388958 70389194 70389266 70389349 70389486 70389716 70389823 70389868 70389916 70389917 70389934 70389935 70390000 70390024 70390051 70390069 70390098 70390135 70390151 70390175 70390221 70390272 70390273 70390281 70390302 70390358 70390449 70390494 70390525 70390528 70390554 70390723 70390777 70390795 70391246 70391344 70391429 70391454 70391536 70391561 70391616 70391655 70391688 70391888 70391986 70392053 70392062 70392874 70392967 70393590 70393660 70393665 70393680 70393780 70394240 70394493 70394500 70394564 70394991 70395188 70395338 70395353 70395596 70395767 70395803 70395821 70395945 70396060 70396093 70396137 70396352 70396415 70396562 70396681 70396710 70397007 70397202 70397236 70397237 70397266 70397274 70397323 70397408 70397424 70397507 70397849 70397899 70397977 70398467 70398606 70398769 70398783 70398857 70399017 70399573 70399615 70399643 70399706 70399774 70399802 70399877 70399972 70400027 70400339 70400391 70400439 70400450 70400462 70400631 70400843 70400884 70400905 70401025 70401093 70401103 70401279 70401513 70401731 70401751 70401818 70401824 70401832 70401931 70402005 70402042 70402181 70402246 70402312 70402805 70402954 70402958 70403070 70403427 70403460 70403503 70403702 70403764 70403815 70404001 70404130 70404144 70404150 70404196 70404759 70404889 70404918 70404922 70405360 70405404 70405545 70405551 70405916 70405973 70406203 70406423 70406450 70406575 70406601 70406851 70406908 70406918 70406939 70406957 70406964 70406966 70407039 70407054 70407123 70407232 70407314 70407359 70407643 70407762 70407772 70407784 70407823 70407993 70408190 70408244 70408309 70408436 70408894 70408922 70408931 70409574 70409688 70409752 70409818 70409826 70410184 70410273 70411070 70411081 70411132 70411395 70412096 70412356 70412437 70412606 70412912 70413031 70413032 70413192 70413508 70413777 70413804 70413905 70414029 70414599 70414640 70414689 70414796 70414930 70414931 70415364 70415522 70415549 70415655 70415744 70415787 70415812 70415877 70415904 70415954 70416013 70416121 70416155 70416321 70416464 70416480 70416486 70416526 70416542 70416600 70416785 70417188 70417215 70417286 70417394 70417495 70417646 70417733 70418085 70418179 70418277 70418279 70418312 70418313 70418395 70418471 70418493 70418650 70418723 70418849 70419361 70419420 70419458 70419473 70419567 70419588 70419884 70419913 70420001 70420426 70420487 70420547 70420639 70420812 70420888 70420900 70421022 70421080 70421227 70421307 70421369 70421486 70421528 70421601 70421618 70421675 70421689 70421771 70421938 70421986 70422100 70422141 70422199 70422296 70422344 70422704 70422727 70422745 70422851 70422890 70422923 70423008 70423014 70423051 70423236 70423325 70423580 70423633 70424215 70424226 70424811 70425058 70425151 70425165 70425203 70425220 70425296 70425337 70425430 70425434 70425826 70426071 70426147 70426383 70426388 70426795 70426866 70426973 70427362 70427554 70427571 70427607 70427623 70427653 70427888 70427947 70428039 70428058 70428064 70428558 70428599 70428840 70428874 70429211 70429409 70429421 70429565 70429748 70429812 70429896 70430041 70430155 70430536 70430879 70430909 70430979 70431063 70431070 70431130 70431261 70431410 70431476 70431505 70431589 70432006 70363728(i) 70370397(i) 70379727(i) 70389268(i) 70389833(i) 70390102(i) 70391979(i) 70397714(i) 70397823(i) 70400235(i) 70400524(i) 70401303(i) 70401741(i) 70402217(i) 70403954(i) 70403982(i) 70403989(i) 70404913(i) 70406212(i) 70406234(i) 70406255(i) 70407786(i) 70411912(i) 70413952(i) 70413961(i) 70414468(i) 70417253(i) 70417543(i) 70418956(i) 70419034(i) 70419720(i) 70421898(i) 70425358(i) 70427192(i) 70427272(i) 70428224(i) 70428533(i) 70430184(i) 70430233(i) 70430545(i) 70430557(i) 70431614(i) |
| CHB (Han Chinese in Beijing, China) | 23% | 500 | 70361741 70362123 70362561 70362834 70363362 70366492 70367102 70367399 70368202 70368400 70368565 70370643 70370804 70371555 70371656 70371657 70372046 70372641 70372905 70372955 70374584 70374588 70375035 70375691 70376112 70376691 70376705 70376708 70376963 70377360 70377361 70377394 70377396 70377402 70377524 70377642 70378396 70378440 70378555 70379374 70379682 70380660 70380675 70381259 70381351 70382077 70382444 70382578 70382579 70382957 70383322 70383625 70383913 70383965 70383991 70384123 70384324 70385252 70385551 70385552 70385588 70385589 70386429 70386623 70386791 70387066 70387375 70387402 70387713 70388194 70388363 70388497 70388537 70388610 70388617 70388623 70388633 70388646 70388704 70388958 70389194 70389266 70389349 70389486 70389716 70389823 70389868 70389916 70389917 70389934 70389935 70390000 70390024 70390051 70390069 70390098 70390135 70390151 70390175 70390221 70390272 70390273 70390281 70390302 70390358 70390449 70390494 70390525 70390528 70390554 70390723 70390777 70390795 70391246 70391344 70391429 70391454 70391536 70391561 70391616 70391655 70391688 70391888 70391986 70392053 70392062 70392874 70392967 70393390 70393590 70393660 70393665 70393680 70393780 70394123 70394240 70394493 70394500 70394564 70394991 70395188 70395338 70395353 70395596 70395767 70395803 70395821 70395945 70396032 70396060 70396093 70396137 70396352 70396415 70396562 70396681 70396710 70397007 70397202 70397236 70397237 70397266 70397274 70397323 70397408 70397424 70397507 70397849 70397899 70397977 70398467 70398606 70398649 70398769 70398783 70398857 70399017 70399573 70399615 70399643 70399706 70399774 70399802 70399877 70399972 70400027 70400339 70400391 70400439 70400450 70400462 70400608 70400631 70400843 70400884 70400905 70401025 70401093 70401103 70401279 70401513 70401731 70401751 70401818 70401824 70401832 70401931 70402005 70402042 70402181 70402246 70402312 70402805 70402881 70402954 70402958 70403070 70403427 70403460 70403503 70403702 70403764 70403815 70404001 70404130 70404144 70404150 70404196 70404759 70404889 70404918 70404922 70405360 70405404 70405453 70405545 70405551 70405837 70405916 70405973 70406203 70406423 70406450 70406575 70406601 70406851 70406908 70406918 70406939 70406957 70406964 70406966 70407039 70407054 70407122 70407123 70407232 70407314 70407359 70407569 70407643 70407762 70407772 70407784 70407823 70407993 70408190 70408244 70408309 70408436 70408894 70408922 70408931 70409525 70409534 70409574 70409688 70409752 70409818 70409826 70409924 70410184 70410273 70411070 70411081 70411132 70411395 70411537 70412096 70412356 70412437 70412606 70412912 70413031 70413032 70413192 70413508 70413777 70413804 70413905 70414029 70414239 70414599 70414640 70414689 70414796 70414930 70414931 70415364 70415522 70415549 70415655 70415744 70415787 70415812 70415877 70415904 70415954 70416013 70416121 70416155 70416321 70416464 70416480 70416486 70416526 70416542 70416600 70416785 70417188 70417215 70417286 70417394 70417495 70417646 70417733 70418085 70418179 70418277 70418279 70418312 70418313 70418395 70418471 70418493 70418650 70418723 70418849 70419361 70419420 70419458 70419473 70419567 70419588 70419884 70419913 70420001 70420008 70420426 70420487 70420547 70420639 70420812 70420888 70420900 70421022 70421080 70421227 70421307 70421369 70421486 70421528 70421601 70421618 70421675 70421689 70421771 70421938 70421986 70422100 70422141 70422199 70422296 70422344 70422704 70422727 70422745 70422800 70422851 70422890 70422923 70423008 70423014 70423051 70423236 70423325 70423580 70423633 70424215 70424226 70424811 70425058 70425151 70425165 70425203 70425220 70425296 70425337 70425430 70425434 70425826 70426071 70426147 70426383 70426388 70426795 70426866 70426973 70427362 70427554 70427571 70427607 70427623 70427653 70427888 70427947 70428039 70428058 70428064 70428558 70428599 70428652 70428840 70428874 70429211 70429409 70429421 70429565 70429748 70429812 70429896 70430041 70430155 70430536 70430879 70430909 70430979 70431063 70431070 70431130 70431261 70431410 70431476 70431505 70431589 70431781 70432006 70363728(i) 70367775(i) 70370397(i) 70379727(i) 70389268(i) 70389833(i) 70390102(i) 70391979(i) 70397714(i) 70397823(i) 70400235(i) 70400524(i) 70401303(i) 70401741(i) 70402217(i) 70403954(i) 70403982(i) 70403989(i) 70404913(i) 70406212(i) 70406234(i) 70406255(i) 70407786(i) 70411912(i) 70413952(i) 70413961(i) 70414468(i) 70417253(i) 70417543(i) 70418956(i) 70419034(i) 70419720(i) 70421898(i) 70425358(i) 70427192(i) 70427272(i) 70428224(i) 70428533(i) 70430184(i) 70430233(i) 70430545(i) 70430557(i) 70431614(i) |
| CHS (Southern Han Chinese) | 30% | 501 | 70361741 70362123 70362561 70362834 70363362 70366324 70366328 70366492 70367102 70367399 70368202 70368400 70368565 70370643 70370804 70371555 70371656 70371657 70372046 70372641 70372905 70372955 70374584 70374588 70375035 70375691 70376112 70376691 70376705 70376708 70376963 70377360 70377361 70377394 70377396 70377402 70377524 70377642 70378396 70378440 70378555 70379374 70379682 70380660 70380675 70381259 70381351 70382077 70382444 70382578 70382579 70382957 70383322 70383625 70383913 70383965 70383991 70384123 70384324 70385252 70385551 70385552 70385588 70385589 70386429 70386623 70386791 70387066 70387375 70387402 70387713 70388194 70388363 70388497 70388537 70388610 70388617 70388623 70388633 70388646 70388704 70388958 70389194 70389266 70389349 70389486 70389716 70389823 70389868 70389916 70389917 70389934 70389935 70390000 70390024 70390051 70390069 70390098 70390135 70390151 70390175 70390221 70390272 70390273 70390281 70390302 70390358 70390449 70390494 70390525 70390528 70390554 70390723 70390777 70390795 70391246 70391344 70391429 70391454 70391536 70391561 70391616 70391655 70391688 70391888 70391986 70392053 70392062 70392874 70392967 70393390 70393590 70393660 70393665 70393680 70393780 70394123 70394240 70394493 70394500 70394564 70394991 70395188 70395338 70395353 70395596 70395767 70395803 70395821 70395945 70396032 70396060 70396093 70396137 70396352 70396415 70396562 70396681 70396710 70397007 70397202 70397236 70397237 70397266 70397274 70397323 70397408 70397424 70397507 70397849 70397899 70397977 70398467 70398606 70398649 70398769 70398783 70398857 70399017 70399573 70399615 70399643 70399706 70399774 70399802 70399877 70399972 70400027 70400339 70400391 70400439 70400450 70400462 70400608 70400631 70400843 70400884 70400905 70401025 70401093 70401103 70401279 70401513 70401731 70401751 70401818 70401824 70401832 70401931 70402005 70402042 70402181 70402246 70402312 70402805 70402881 70402954 70402958 70403070 70403427 70403460 70403503 70403702 70403764 70403815 70404001 70404130 70404144 70404150 70404196 70404759 70404889 70404918 70404922 70405360 70405404 70405453 70405545 70405551 70405837 70405916 70405973 70406203 70406423 70406450 70406575 70406601 70406851 70406908 70406918 70406939 70406957 70406964 70406966 70407039 70407054 70407123 70407232 70407314 70407359 70407569 70407643 70407762 70407772 70407784 70407823 70407993 70408190 70408244 70408309 70408436 70408894 70408922 70408931 70409525 70409534 70409574 70409688 70409752 70409818 70409826 70409924 70410184 70410273 70411070 70411081 70411132 70411395 70411537 70412096 70412356 70412437 70412606 70412912 70413031 70413032 70413192 70413508 70413777 70413804 70413905 70414029 70414239 70414599 70414640 70414689 70414796 70414930 70414931 70415364 70415522 70415549 70415655 70415744 70415787 70415812 70415877 70415904 70415954 70416013 70416121 70416155 70416321 70416464 70416480 70416486 70416526 70416542 70416600 70416785 70417188 70417215 70417286 70417394 70417495 70417646 70417733 70418085 70418179 70418277 70418279 70418312 70418313 70418395 70418471 70418493 70418650 70418723 70418849 70419361 70419420 70419458 70419473 70419567 70419588 70419884 70419913 70420001 70420008 70420426 70420487 70420547 70420639 70420812 70420888 70420900 70421022 70421080 70421227 70421307 70421369 70421486 70421528 70421601 70421618 70421675 70421689 70421771 70421938 70421986 70422100 70422141 70422199 70422296 70422344 70422704 70422727 70422745 70422800 70422851 70422890 70422923 70423008 70423014 70423051 70423236 70423325 70423580 70423633 70424215 70424226 70424811 70425058 70425151 70425165 70425203 70425220 70425296 70425337 70425430 70425434 70425826 70426071 70426147 70426383 70426388 70426795 70426866 70426973 70427362 70427554 70427571 70427607 70427623 70427653 70427888 70427947 70428039 70428058 70428064 70428558 70428599 70428652 70428840 70428874 70429211 70429409 70429421 70429565 70429748 70429812 70429896 70430041 70430155 70430536 70430879 70430909 70430979 70431063 70431070 70431130 70431261 70431410 70431476 70431505 70431589 70431781 70432006 70363728(i) 70367775(i) 70370397(i) 70379727(i) 70389268(i) 70389833(i) 70390102(i) 70391979(i) 70397714(i) 70397823(i) 70400235(i) 70400524(i) 70401303(i) 70401741(i) 70402217(i) 70403954(i) 70403982(i) 70403989(i) 70404913(i) 70406212(i) 70406234(i) 70406255(i) 70407786(i) 70411912(i) 70413952(i) 70413961(i) 70414468(i) 70417253(i) 70417543(i) 70418956(i) 70419034(i) 70419720(i) 70421898(i) 70425358(i) 70427192(i) 70427272(i) 70428224(i) 70428533(i) 70430184(i) 70430233(i) 70430545(i) 70430557(i) 70431614(i) |
| CLM (Colombians from Medellin, Colombia) | 35% | 456 | 70361741 70362123 70367399 70372905 70372955 70374584 70374588 70375691 70376112 70376705 70376708 70376963 70377360 70377361 70377394 70377396 70377402 70377524 70377642 70378396 70378440 70378555 70379374 70379682 70380660 70380675 70381259 70381351 70382444 70382578 70382579 70383322 70383913 70383965 70383991 70384123 70384324 70385252 70385552 70385588 70385589 70386429 70386623 70386791 70387066 70387375 70388363 70388497 70388537 70388610 70388617 70388623 70388633 70388646 70388704 70388958 70389194 70389266 70389349 70389486 70389716 70389823 70389868 70389916 70389917 70389934 70389935 70390000 70390024 70390051 70390069 70390098 70390135 70390151 70390175 70390221 70390272 70390273 70390281 70390302 70390358 70390449 70390494 70390525 70390528 70390554 70390723 70390777 70390795 70391246 70391344 70391429 70391454 70391536 70391561 70391616 70391655 70391688 70391888 70391986 70392053 70392062 70392874 70392967 70393590 70393660 70393665 70393680 70393780 70394240 70394493 70394500 70394564 70394991 70395188 70395338 70395353 70395596 70395767 70395803 70395821 70395945 70396060 70396093 70396137 70396352 70396415 70396562 70396681 70396710 70397007 70397202 70397236 70397237 70397266 70397274 70397323 70397408 70397424 70397507 70397849 70397899 70397977 70398467 70398606 70398769 70398783 70398857 70399017 70399573 70399615 70399643 70399706 70399774 70399802 70399877 70399972 70400027 70400339 70400391 70400439 70400450 70400462 70400631 70400843 70400884 70400905 70401025 70401093 70401103 70401279 70401513 70401731 70401751 70401818 70401824 70401832 70401931 70402005 70402042 70402181 70402246 70402312 70402805 70402954 70402958 70403070 70403427 70403460 70403503 70403702 70403764 70403815 70404001 70404130 70404144 70404150 70404196 70404759 70404889 70404918 70404922 70405360 70405404 70405545 70405551 70405916 70405973 70406203 70406423 70406450 70406575 70406601 70406851 70406908 70406918 70406939 70406957 70406964 70406966 70407039 70407054 70407123 70407232 70407314 70407359 70407643 70407762 70407772 70407784 70407823 70407993 70408190 70408244 70408309 70408436 70408894 70408922 70408931 70409574 70409688 70409752 70409818 70409826 70410184 70410273 70411070 70411081 70411132 70411395 70412096 70412356 70412437 70412606 70412912 70413031 70413032 70413192 70413508 70413777 70413804 70413905 70414029 70414599 70414640 70414689 70414796 70414930 70414931 70415364 70415522 70415549 70415655 70415744 70415787 70415812 70415877 70415904 70415954 70416013 70416121 70416155 70416321 70416464 70416480 70416486 70416526 70416542 70416600 70416785 70417188 70417215 70417286 70417394 70417495 70417646 70417733 70418085 70418179 70418277 70418279 70418312 70418313 70418395 70418471 70418493 70418650 70418723 70418849 70419361 70419420 70419458 70419473 70419567 70419588 70419884 70419913 70420001 70420426 70420487 70420547 70420639 70420812 70420888 70420900 70421022 70421080 70421227 70421307 70421369 70421486 70421528 70421601 70421618 70421675 70421689 70421771 70421938 70421986 70422100 70422141 70422199 70422296 70422344 70422704 70422727 70422745 70422851 70422890 70422923 70423008 70423014 70423051 70423236 70423325 70423580 70423633 70424215 70424226 70424811 70425058 70425151 70425165 70425203 70425220 70425296 70425337 70425430 70425434 70425826 70426071 70426147 70426383 70426388 70426795 70426866 70426973 70427362 70427554 70427571 70427607 70427623 70427653 70427888 70427947 70428039 70428058 70428064 70428558 70428599 70428840 70428874 70429211 70429409 70429421 70429565 70429748 70429812 70429896 70430041 70430155 70430536 70430879 70430909 70430979 70431063 70431070 70431130 70431261 70431410 70431476 70431505 70431589 70432006 70363728(i) 70370397(i) 70379727(i) 70389268(i) 70389833(i) 70390102(i) 70391979(i) 70397714(i) 70397823(i) 70400235(i) 70400524(i) 70401303(i) 70401741(i) 70402217(i) 70403954(i) 70403982(i) 70403989(i) 70404913(i) 70406212(i) 70406234(i) 70406255(i) 70407786(i) 70411912(i) 70413952(i) 70413961(i) 70414468(i) 70417253(i) 70417543(i) 70418956(i) 70419034(i) 70419720(i) 70421898(i) 70425358(i) 70427192(i) 70427272(i) 70428224(i) 70428533(i) 70430184(i) 70430233(i) 70430545(i) 70430557(i) 70431614(i) |
| ESN (Esan in Nigeria) | 32% | 400 | 70376708 70376963 70377360 70377361 70377394 70377396 70377402 70377524 70377642 70378555 70379374 70381259 70382444 70382578 70382579 70383965 70383991 70385252 70385552 70385588 70385589 70386623 70386791 70387066 70387375 70388363 70388497 70388537 70388610 70388617 70388623 70388633 70388704 70388958 70389194 70389266 70389349 70389486 70389823 70389868 70389916 70389917 70389934 70389935 70390000 70390024 70390051 70390069 70390098 70390135 70390151 70390175 70390221 70390272 70390273 70390281 70390302 70390358 70390449 70390494 70390528 70390554 70390723 70390777 70390795 70391246 70391344 70391429 70391454 70391536 70391561 70391616 70391655 70391688 70391986 70392053 70392062 70392874 70392967 70393660 70393665 70393680 70393780 70394240 70394493 70394500 70394564 70394991 70395188 70395338 70395596 70395767 70395803 70395821 70396060 70396093 70396137 70396352 70396415 70396562 70396681 70396710 70397007 70397202 70397236 70397237 70397266 70397274 70397323 70397408 70397424 70397507 70397849 70397899 70398467 70398606 70398769 70398857 70399017 70399573 70399615 70399774 70399877 70399972 70400027 70400339 70400391 70400450 70400462 70400631 70400843 70400884 70400905 70401025 70401093 70401103 70401279 70401513 70401731 70401751 70401818 70401824 70401832 70401931 70402005 70402042 70402181 70402246 70402312 70402805 70402954 70402958 70403070 70403427 70403460 70403503 70403702 70403764 70403815 70404001 70404130 70404144 70404150 70404196 70404759 70404889 70404918 70404922 70405360 70405404 70405545 70405551 70405916 70405973 70406203 70406423 70406450 70406601 70406851 70406908 70406918 70406939 70406964 70406966 70407039 70407054 70407123 70407232 70407359 70407643 70407762 70407772 70407784 70407823 70407993 70408190 70408309 70408436 70408894 70408922 70408931 70409525 70409534 70409574 70409752 70409818 70409826 70410184 70410273 70411070 70411081 70411132 70411395 70412096 70412356 70412437 70412606 70412912 70413031 70413032 70413192 70413508 70413777 70413804 70413905 70414029 70414599 70414640 70414689 70414796 70414930 70414931 70415364 70415522 70415549 70415655 70415744 70415787 70415812 70415877 70415904 70415954 70416013 70416155 70416321 70416464 70416480 70416486 70416526 70416542 70416600 70416785 70417188 70417215 70417286 70417394 70417495 70417646 70417733 70418085 70418179 70418277 70418279 70418312 70418313 70418395 70418471 70418493 70418650 70418723 70418849 70419361 70419420 70419458 70419473 70419567 70419588 70419884 70419913 70420426 70420487 70420547 70420639 70420812 70420888 70421022 70421080 70421227 70421307 70421369 70421601 70421618 70421675 70421689 70421771 70421938 70421986 70422100 70422141 70422199 70422296 70422344 70422704 70422727 70422745 70422851 70422923 70423008 70423014 70423051 70423236 70423325 70423580 70423633 70424215 70424226 70424811 70425058 70425151 70425165 70425203 70425220 70425296 70425337 70425430 70425434 70425826 70426147 70426383 70426388 70426795 70426866 70427362 70427571 70427607 70427623 70427653 70427888 70427947 70428039 70428058 70428064 70428558 70428840 70428874 70429211 70429421 70429565 70429748 70429812 70429896 70430041 70430155 70430536 70430879 70430909 70430979 70431063 70431070 70431130 70431261 70431410 70431505 70431589 70379727(i) 70389268(i) 70389833(i) 70390102(i) 70397714(i) 70397823(i) 70400235(i) 70400524(i) 70401303(i) 70401741(i) 70403954(i) 70403982(i) 70403989(i) 70404913(i) 70406212(i) 70406234(i) 70406255(i) 70407786(i) 70411912(i) 70413952(i) 70413961(i) 70414468(i) 70417543(i) 70419034(i) 70419720(i) 70421898(i) 70425358(i) 70427192(i) 70427272(i) 70428224(i) 70428533(i) 70430184(i) 70430233(i) 70430545(i) 70430557(i) 70431614(i) |
| FIN (Finnish in Finland) | 39% | 458 | 70361741 70362123 70367399 70372905 70372955 70374584 70374588 70375691 70376112 70376705 70376708 70376963 70377360 70377361 70377394 70377396 70377402 70377524 70377642 70378396 70378440 70378555 70379374 70379682 70380660 70380675 70381259 70381351 70382444 70382578 70382579 70383322 70383913 70383965 70383991 70384123 70384324 70385252 70385552 70385588 70385589 70386429 70386623 70386791 70387066 70387375 70388363 70388497 70388537 70388610 70388617 70388623 70388633 70388646 70388704 70388958 70389194 70389266 70389349 70389486 70389716 70389823 70389868 70389916 70389917 70389934 70389935 70390000 70390024 70390051 70390069 70390098 70390135 70390151 70390175 70390221 70390272 70390273 70390281 70390302 70390358 70390449 70390494 70390525 70390528 70390554 70390723 70390777 70390795 70391246 70391344 70391429 70391454 70391536 70391561 70391616 70391655 70391688 70391888 70391986 70392053 70392062 70392874 70392967 70393590 70393660 70393665 70393680 70393780 70394240 70394493 70394500 70394564 70394991 70395188 70395338 70395353 70395596 70395767 70395803 70395821 70395945 70396060 70396093 70396137 70396352 70396415 70396562 70396681 70396710 70397007 70397202 70397236 70397237 70397266 70397274 70397323 70397408 70397424 70397507 70397849 70397899 70397977 70398467 70398606 70398769 70398783 70398857 70399017 70399573 70399615 70399643 70399706 70399774 70399802 70399877 70399972 70400027 70400339 70400391 70400439 70400450 70400462 70400631 70400843 70400884 70400905 70401025 70401093 70401103 70401279 70401513 70401731 70401751 70401818 70401824 70401832 70401931 70402005 70402042 70402181 70402246 70402312 70402805 70402954 70402958 70403070 70403427 70403460 70403503 70403702 70403764 70403815 70404001 70404130 70404144 70404150 70404196 70404759 70404889 70404918 70404922 70405360 70405404 70405545 70405551 70405916 70405973 70406203 70406423 70406450 70406575 70406601 70406851 70406908 70406918 70406939 70406957 70406964 70406966 70407039 70407054 70407123 70407232 70407314 70407359 70407643 70407762 70407772 70407784 70407823 70407993 70408190 70408244 70408309 70408436 70408894 70408922 70408931 70409525 70409534 70409574 70409688 70409752 70409818 70409826 70410184 70410273 70411070 70411081 70411132 70411395 70412096 70412356 70412437 70412606 70412912 70413031 70413032 70413192 70413508 70413777 70413804 70413905 70414029 70414599 70414640 70414689 70414796 70414930 70414931 70415364 70415522 70415549 70415655 70415744 70415787 70415812 70415877 70415904 70415954 70416013 70416121 70416155 70416321 70416464 70416480 70416486 70416526 70416542 70416600 70416785 70417188 70417215 70417286 70417394 70417495 70417646 70417733 70418085 70418179 70418277 70418279 70418312 70418313 70418395 70418471 70418493 70418650 70418723 70418849 70419361 70419420 70419458 70419473 70419567 70419588 70419884 70419913 70420001 70420426 70420487 70420547 70420639 70420812 70420888 70420900 70421022 70421080 70421227 70421307 70421369 70421486 70421528 70421601 70421618 70421675 70421689 70421771 70421938 70421986 70422100 70422141 70422199 70422296 70422344 70422704 70422727 70422745 70422851 70422890 70422923 70423008 70423014 70423051 70423236 70423325 70423580 70423633 70424215 70424226 70424811 70425058 70425151 70425165 70425203 70425220 70425296 70425337 70425430 70425434 70425826 70426071 70426147 70426383 70426388 70426795 70426866 70426973 70427362 70427554 70427571 70427607 70427623 70427653 70427888 70427947 70428039 70428058 70428064 70428558 70428599 70428840 70428874 70429211 70429409 70429421 70429565 70429748 70429812 70429896 70430041 70430155 70430536 70430879 70430909 70430979 70431063 70431070 70431130 70431261 70431410 70431476 70431505 70431589 70432006 70363728(i) 70370397(i) 70379727(i) 70389268(i) 70389833(i) 70390102(i) 70391979(i) 70397714(i) 70397823(i) 70400235(i) 70400524(i) 70401303(i) 70401741(i) 70402217(i) 70403954(i) 70403982(i) 70403989(i) 70404913(i) 70406212(i) 70406234(i) 70406255(i) 70407786(i) 70411912(i) 70413952(i) 70413961(i) 70414468(i) 70417253(i) 70417543(i) 70418956(i) 70419034(i) 70419720(i) 70421898(i) 70425358(i) 70427192(i) 70427272(i) 70428224(i) 70428533(i) 70430184(i) 70430233(i) 70430545(i) 70430557(i) 70431614(i) |
| GBR (British in England and Scotland) | 38% | 458 | 70361741 70362123 70367399 70372905 70372955 70374584 70374588 70375691 70376112 70376705 70376708 70376963 70377360 70377361 70377394 70377396 70377402 70377524 70377642 70378396 70378440 70378555 70379374 70379682 70380660 70380675 70381259 70381351 70382444 70382578 70382579 70383322 70383913 70383965 70383991 70384123 70384324 70385252 70385552 70385588 70385589 70386429 70386623 70386791 70387066 70387375 70388363 70388497 70388537 70388610 70388617 70388623 70388633 70388646 70388704 70388958 70389194 70389266 70389349 70389486 70389716 70389823 70389868 70389916 70389917 70389934 70389935 70390000 70390024 70390051 70390069 70390098 70390135 70390151 70390175 70390221 70390272 70390273 70390281 70390302 70390358 70390449 70390494 70390525 70390528 70390554 70390723 70390777 70390795 70391246 70391344 70391429 70391454 70391536 70391561 70391616 70391655 70391688 70391888 70391986 70392053 70392062 70392874 70392967 70393590 70393660 70393665 70393680 70393780 70394240 70394493 70394500 70394564 70394991 70395188 70395338 70395353 70395596 70395767 70395803 70395821 70395945 70396060 70396093 70396137 70396352 70396415 70396562 70396681 70396710 70397007 70397202 70397236 70397237 70397266 70397274 70397323 70397408 70397424 70397507 70397849 70397899 70397977 70398467 70398606 70398769 70398783 70398857 70399017 70399573 70399615 70399643 70399706 70399774 70399802 70399877 70399972 70400027 70400339 70400391 70400439 70400450 70400462 70400631 70400843 70400884 70400905 70401025 70401093 70401103 70401279 70401513 70401731 70401751 70401818 70401824 70401832 70401931 70402005 70402042 70402181 70402246 70402312 70402805 70402954 70402958 70403070 70403427 70403460 70403503 70403702 70403764 70403815 70404001 70404130 70404144 70404150 70404196 70404759 70404889 70404918 70404922 70405360 70405404 70405545 70405551 70405916 70405973 70406203 70406423 70406450 70406575 70406601 70406851 70406908 70406918 70406939 70406957 70406964 70406966 70407039 70407054 70407123 70407232 70407314 70407359 70407643 70407762 70407772 70407784 70407823 70407993 70408190 70408244 70408309 70408436 70408894 70408922 70408931 70409525 70409534 70409574 70409688 70409752 70409818 70409826 70410184 70410273 70411070 70411081 70411132 70411395 70412096 70412356 70412437 70412606 70412912 70413031 70413032 70413192 70413508 70413777 70413804 70413905 70414029 70414599 70414640 70414689 70414796 70414930 70414931 70415364 70415522 70415549 70415655 70415744 70415787 70415812 70415877 70415904 70415954 70416013 70416121 70416155 70416321 70416464 70416480 70416486 70416526 70416542 70416600 70416785 70417188 70417215 70417286 70417394 70417495 70417646 70417733 70418085 70418179 70418277 70418279 70418312 70418313 70418395 70418471 70418493 70418650 70418723 70418849 70419361 70419420 70419458 70419473 70419567 70419588 70419884 70419913 70420001 70420426 70420487 70420547 70420639 70420812 70420888 70420900 70421022 70421080 70421227 70421307 70421369 70421486 70421528 70421601 70421618 70421675 70421689 70421771 70421938 70421986 70422100 70422141 70422199 70422296 70422344 70422704 70422727 70422745 70422851 70422890 70422923 70423008 70423014 70423051 70423236 70423325 70423580 70423633 70424215 70424226 70424811 70425058 70425151 70425165 70425203 70425220 70425296 70425337 70425430 70425434 70425826 70426071 70426147 70426383 70426388 70426795 70426866 70426973 70427362 70427554 70427571 70427607 70427623 70427653 70427888 70427947 70428039 70428058 70428064 70428558 70428599 70428840 70428874 70429211 70429409 70429421 70429565 70429748 70429812 70429896 70430041 70430155 70430536 70430879 70430909 70430979 70431063 70431070 70431130 70431261 70431410 70431476 70431505 70431589 70432006 70363728(i) 70370397(i) 70379727(i) 70389268(i) 70389833(i) 70390102(i) 70391979(i) 70397714(i) 70397823(i) 70400235(i) 70400524(i) 70401303(i) 70401741(i) 70402217(i) 70403954(i) 70403982(i) 70403989(i) 70404913(i) 70406212(i) 70406234(i) 70406255(i) 70407786(i) 70411912(i) 70413952(i) 70413961(i) 70414468(i) 70417253(i) 70417543(i) 70418956(i) 70419034(i) 70419720(i) 70421898(i) 70425358(i) 70427192(i) 70427272(i) 70428224(i) 70428533(i) 70430184(i) 70430233(i) 70430545(i) 70430557(i) 70431614(i) |
| GIH (Gujarati Indian from Houston, Texas) | 30% | 456 | 70362123 70367399 70372905 70372955 70374584 70374588 70375691 70376112 70376705 70376708 70376963 70377360 70377361 70377394 70377396 70377402 70377524 70377642 70378396 70378440 70378555 70379374 70379682 70380660 70380675 70381259 70381351 70382444 70382578 70382579 70383322 70383913 70383965 70383991 70384123 70384324 70385252 70385552 70385588 70385589 70386429 70386623 70386791 70387066 70387375 70388363 70388497 70388537 70388610 70388617 70388623 70388633 70388646 70388704 70388958 70389194 70389266 70389349 70389486 70389716 70389823 70389868 70389916 70389917 70389934 70389935 70390000 70390024 70390051 70390069 70390098 70390135 70390151 70390175 70390221 70390272 70390273 70390281 70390302 70390358 70390449 70390494 70390525 70390528 70390554 70390723 70390777 70390795 70391246 70391344 70391429 70391454 70391536 70391561 70391616 70391655 70391688 70391888 70391986 70392053 70392062 70392967 70393590 70393660 70393665 70393680 70393780 70394240 70394493 70394500 70394564 70394991 70395188 70395338 70395353 70395596 70395767 70395803 70395821 70395945 70396060 70396093 70396137 70396352 70396415 70396562 70396681 70396710 70397007 70397202 70397236 70397237 70397266 70397274 70397323 70397408 70397424 70397507 70397849 70397899 70397977 70398467 70398606 70398769 70398783 70398857 70399017 70399573 70399615 70399643 70399706 70399774 70399802 70399877 70399972 70400027 70400339 70400391 70400439 70400450 70400462 70400631 70400843 70400884 70400905 70401025 70401093 70401103 70401279 70401513 70401731 70401751 70401818 70401824 70401832 70401931 70402005 70402042 70402181 70402246 70402312 70402805 70402954 70402958 70403070 70403427 70403460 70403503 70403702 70403764 70403815 70404001 70404130 70404144 70404150 70404196 70404759 70404889 70404918 70404922 70405360 70405404 70405545 70405551 70405916 70405973 70406203 70406423 70406450 70406575 70406601 70406851 70406908 70406918 70406939 70406957 70406964 70406966 70407039 70407054 70407123 70407232 70407314 70407359 70407643 70407762 70407772 70407784 70407823 70407993 70408190 70408244 70408309 70408436 70408894 70408922 70408931 70409525 70409534 70409574 70409688 70409752 70409818 70409826 70410184 70410273 70411070 70411081 70411132 70411395 70412096 70412356 70412437 70412606 70412912 70413031 70413032 70413192 70413508 70413777 70413804 70413905 70414029 70414599 70414640 70414689 70414796 70414930 70414931 70415364 70415522 70415549 70415655 70415744 70415787 70415812 70415877 70415904 70415954 70416013 70416121 70416155 70416321 70416464 70416480 70416486 70416526 70416542 70416600 70416785 70417188 70417215 70417286 70417394 70417495 70417646 70417733 70418085 70418179 70418277 70418279 70418312 70418313 70418395 70418471 70418493 70418650 70418723 70418849 70419361 70419420 70419458 70419473 70419567 70419588 70419884 70419913 70420001 70420426 70420487 70420547 70420639 70420812 70420888 70420900 70421022 70421080 70421227 70421307 70421369 70421486 70421528 70421601 70421618 70421675 70421689 70421771 70421938 70421986 70422100 70422141 70422199 70422296 70422344 70422704 70422727 70422745 70422851 70422890 70422923 70423008 70423014 70423051 70423236 70423325 70423580 70423633 70424215 70424226 70424811 70425058 70425151 70425165 70425203 70425220 70425296 70425337 70425430 70425434 70425826 70426071 70426147 70426383 70426388 70426795 70426866 70426973 70427362 70427554 70427571 70427607 70427623 70427653 70427888 70427947 70428039 70428058 70428064 70428558 70428599 70428840 70428874 70429211 70429409 70429421 70429565 70429748 70429812 70429896 70430041 70430155 70430536 70430879 70430909 70430979 70431063 70431070 70431130 70431261 70431410 70431476 70431505 70431589 70432006 70363728(i) 70370397(i) 70379727(i) 70389268(i) 70389833(i) 70390102(i) 70391979(i) 70397714(i) 70397823(i) 70400235(i) 70400524(i) 70401303(i) 70401741(i) 70402217(i) 70403954(i) 70403982(i) 70403989(i) 70404913(i) 70406212(i) 70406234(i) 70406255(i) 70407786(i) 70411912(i) 70413952(i) 70413961(i) 70414468(i) 70417253(i) 70417543(i) 70418956(i) 70419034(i) 70419720(i) 70421898(i) 70425358(i) 70427192(i) 70427272(i) 70428224(i) 70428533(i) 70430184(i) 70430233(i) 70430545(i) 70430557(i) 70431614(i) |
| GWD (Gambian in Western Divisions in the Gambia) | 36% | 411 | 70361741 70362561 70362834 70363362 70367102 70368202 70368400 70368565 70370643 70371555 70371656 70371657 70372641 70375035 70376691 70376963 70377360 70377361 70377394 70377396 70377402 70377524 70377642 70378555 70379374 70381259 70382077 70382444 70382578 70382579 70383625 70383965 70383991 70385252 70385552 70385588 70385589 70386623 70386791 70387066 70387402 70388363 70388497 70388537 70388610 70388617 70388623 70388633 70388704 70388958 70389194 70389266 70389349 70389486 70389823 70389868 70389916 70389917 70389934 70389935 70390000 70390024 70390051 70390069 70390098 70390135 70390151 70390175 70390221 70390272 70390273 70390281 70390302 70390358 70390449 70390494 70390528 70390554 70390723 70390777 70390795 70391246 70391344 70391429 70391454 70391536 70391561 70391616 70391655 70391688 70391986 70392053 70392062 70392874 70392967 70393390 70393660 70393665 70393680 70393780 70394240 70394493 70394500 70394564 70394991 70395188 70395338 70395596 70395767 70395803 70395821 70396032 70396060 70396093 70396137 70396352 70396415 70396562 70396681 70396710 70397007 70397202 70397236 70397237 70397266 70397274 70397323 70397408 70397424 70397507 70397849 70397899 70398467 70398606 70398649 70398769 70398857 70399017 70399573 70399615 70399774 70399877 70399972 70400027 70400339 70400391 70400450 70400462 70400608 70400631 70400843 70400884 70400905 70401025 70401093 70401103 70401279 70401513 70401731 70401751 70401818 70401824 70401832 70401931 70402005 70402042 70402181 70402312 70402805 70402881 70402954 70402958 70403070 70403427 70403460 70403503 70403702 70403764 70403815 70404001 70404130 70404144 70404150 70404196 70404759 70404889 70404922 70405360 70405404 70405453 70405545 70405551 70405837 70405916 70405973 70406203 70406423 70406450 70406601 70406851 70406908 70406918 70406939 70406964 70406966 70407039 70407054 70407122 70407123 70407232 70407359 70407643 70407762 70407772 70407784 70407823 70407993 70408190 70408436 70409574 70409752 70409818 70409826 70410184 70410273 70411070 70411081 70411132 70411395 70412096 70412356 70412437 70412606 70412912 70413031 70413032 70413192 70413508 70413804 70413905 70414029 70414599 70414640 70414689 70414796 70414930 70414931 70415364 70415522 70415549 70415655 70415744 70415787 70415812 70415877 70415904 70415954 70416013 70416155 70416321 70416464 70416480 70416486 70416526 70416542 70416600 70416785 70417188 70417215 70417286 70417394 70417495 70417646 70417733 70418085 70418179 70418277 70418279 70418312 70418313 70418395 70418471 70418493 70418650 70418723 70418849 70419361 70419420 70419458 70419473 70419567 70419588 70419884 70419913 70420426 70420487 70420547 70420639 70420812 70420888 70421022 70421080 70421227 70421307 70421369 70421601 70421618 70421675 70421689 70421771 70421938 70421986 70422100 70422141 70422199 70422344 70422745 70422851 70422923 70423008 70423014 70423051 70423236 70423325 70423580 70423633 70424215 70424226 70424811 70425058 70425151 70425165 70425203 70425220 70425296 70425337 70425430 70425434 70425826 70426147 70426383 70426388 70426795 70426866 70427362 70427571 70427607 70427623 70427653 70427888 70427947 70428039 70428058 70428064 70428558 70428840 70428874 70429211 70429421 70429565 70429748 70429812 70429896 70430041 70430155 70430536 70430879 70430909 70430979 70431063 70431070 70431130 70431261 70431410 70431505 70431589 70389268(i) 70389833(i) 70390102(i) 70397714(i) 70397823(i) 70400235(i) 70400524(i) 70401303(i) 70401741(i) 70403954(i) 70403982(i) 70403989(i) 70404913(i) 70406212(i) 70406234(i) 70406255(i) 70407786(i) 70411912(i) 70413952(i) 70413961(i) 70414468(i) 70417543(i) 70419034(i) 70419720(i) 70421898(i) 70425358(i) 70427192(i) 70427272(i) 70428224(i) 70428533(i) 70430184(i) 70430233(i) 70430545(i) 70430557(i) 70431614(i) |
| IBS (Iberian Population in Spain) | 37% | 456 | 70361741 70362123 70367399 70372905 70372955 70374584 70374588 70375691 70376112 70376705 70376708 70376963 70377360 70377361 70377394 70377396 70377402 70377524 70377642 70378396 70378440 70378555 70379374 70379682 70380660 70380675 70381259 70381351 70382444 70382578 70382579 70383322 70383913 70383965 70383991 70384123 70384324 70385252 70385552 70385588 70385589 70386429 70386623 70386791 70387066 70387375 70388363 70388497 70388537 70388610 70388617 70388623 70388633 70388646 70388704 70388958 70389194 70389266 70389349 70389486 70389716 70389823 70389868 70389916 70389917 70389934 70389935 70390000 70390024 70390051 70390069 70390098 70390135 70390151 70390175 70390221 70390272 70390273 70390281 70390302 70390358 70390449 70390494 70390525 70390528 70390554 70390723 70390777 70390795 70391246 70391344 70391429 70391454 70391536 70391561 70391616 70391655 70391688 70391888 70391986 70392053 70392062 70392874 70392967 70393590 70393660 70393665 70393680 70393780 70394240 70394493 70394500 70394564 70394991 70395188 70395338 70395353 70395596 70395767 70395803 70395821 70395945 70396060 70396093 70396137 70396352 70396415 70396562 70396681 70396710 70397007 70397202 70397236 70397237 70397266 70397274 70397323 70397408 70397424 70397507 70397849 70397899 70397977 70398467 70398606 70398769 70398783 70398857 70399017 70399573 70399615 70399643 70399706 70399774 70399802 70399877 70399972 70400027 70400339 70400391 70400439 70400450 70400462 70400631 70400843 70400884 70400905 70401025 70401093 70401103 70401279 70401513 70401731 70401751 70401818 70401824 70401832 70401931 70402005 70402042 70402181 70402246 70402312 70402805 70402954 70402958 70403070 70403427 70403460 70403503 70403702 70403764 70403815 70404001 70404130 70404144 70404150 70404196 70404759 70404889 70404918 70404922 70405360 70405404 70405545 70405551 70405916 70405973 70406203 70406423 70406450 70406575 70406601 70406851 70406908 70406918 70406939 70406957 70406964 70406966 70407039 70407054 70407123 70407232 70407314 70407359 70407643 70407762 70407772 70407784 70407823 70407993 70408190 70408244 70408309 70408436 70408894 70408922 70408931 70409574 70409688 70409752 70409818 70409826 70410184 70410273 70411070 70411081 70411132 70411395 70412096 70412356 70412437 70412606 70412912 70413031 70413032 70413192 70413508 70413777 70413804 70413905 70414029 70414599 70414640 70414689 70414796 70414930 70414931 70415364 70415522 70415549 70415655 70415744 70415787 70415812 70415877 70415904 70415954 70416013 70416121 70416155 70416321 70416464 70416480 70416486 70416526 70416542 70416600 70416785 70417188 70417215 70417286 70417394 70417495 70417646 70417733 70418085 70418179 70418277 70418279 70418312 70418313 70418395 70418471 70418493 70418650 70418723 70418849 70419361 70419420 70419458 70419473 70419567 70419588 70419884 70419913 70420001 70420426 70420487 70420547 70420639 70420812 70420888 70420900 70421022 70421080 70421227 70421307 70421369 70421486 70421528 70421601 70421618 70421675 70421689 70421771 70421938 70421986 70422100 70422141 70422199 70422296 70422344 70422704 70422727 70422745 70422851 70422890 70422923 70423008 70423014 70423051 70423236 70423325 70423580 70423633 70424215 70424226 70424811 70425058 70425151 70425165 70425203 70425220 70425296 70425337 70425430 70425434 70425826 70426071 70426147 70426383 70426388 70426795 70426866 70426973 70427362 70427554 70427571 70427607 70427623 70427653 70427888 70427947 70428039 70428058 70428064 70428558 70428599 70428840 70428874 70429211 70429409 70429421 70429565 70429748 70429812 70429896 70430041 70430155 70430536 70430879 70430909 70430979 70431063 70431070 70431130 70431261 70431410 70431476 70431505 70431589 70432006 70363728(i) 70370397(i) 70379727(i) 70389268(i) 70389833(i) 70390102(i) 70391979(i) 70397714(i) 70397823(i) 70400235(i) 70400524(i) 70401303(i) 70401741(i) 70402217(i) 70403954(i) 70403982(i) 70403989(i) 70404913(i) 70406212(i) 70406234(i) 70406255(i) 70407786(i) 70411912(i) 70413952(i) 70413961(i) 70414468(i) 70417253(i) 70417543(i) 70418956(i) 70419034(i) 70419720(i) 70421898(i) 70425358(i) 70427192(i) 70427272(i) 70428224(i) 70428533(i) 70430184(i) 70430233(i) 70430545(i) 70430557(i) 70431614(i) |
| ITU (Indian Telugu from the UK) | 28% | 456 | 70361741 70362123 70367399 70372905 70372955 70374584 70374588 70375691 70376112 70376963 70377360 70377361 70377394 70377396 70377402 70377524 70377642 70378396 70378440 70378555 70379374 70379682 70380660 70380675 70381259 70381351 70382444 70382578 70382579 70383322 70383913 70383965 70383991 70384123 70384324 70385252 70385552 70385588 70385589 70386429 70386623 70386791 70387066 70387375 70388363 70388497 70388537 70388610 70388617 70388623 70388633 70388646 70388704 70388958 70389194 70389266 70389349 70389486 70389716 70389823 70389868 70389916 70389917 70389934 70389935 70390000 70390024 70390051 70390069 70390098 70390135 70390151 70390175 70390221 70390272 70390273 70390281 70390302 70390358 70390449 70390494 70390525 70390528 70390554 70390723 70390777 70390795 70391246 70391344 70391429 70391454 70391536 70391561 70391616 70391655 70391688 70391888 70391986 70392053 70392062 70392874 70392967 70393590 70393660 70393665 70393680 70393780 70394240 70394493 70394500 70394564 70394991 70395188 70395338 70395353 70395596 70395767 70395803 70395821 70395945 70396060 70396093 70396137 70396352 70396415 70396562 70396681 70396710 70397007 70397202 70397236 70397237 70397266 70397274 70397323 70397408 70397424 70397507 70397849 70397899 70397977 70398467 70398606 70398769 70398783 70398857 70399017 70399573 70399615 70399643 70399706 70399774 70399802 70399877 70399972 70400027 70400339 70400391 70400439 70400450 70400462 70400631 70400843 70400884 70400905 70401025 70401093 70401103 70401279 70401513 70401731 70401751 70401818 70401824 70401832 70401931 70402005 70402042 70402181 70402246 70402312 70402805 70402954 70402958 70403070 70403427 70403460 70403503 70403702 70403764 70403815 70404001 70404130 70404144 70404150 70404196 70404759 70404889 70404918 70404922 70405360 70405404 70405545 70405551 70405916 70405973 70406203 70406423 70406450 70406575 70406601 70406851 70406908 70406918 70406939 70406957 70406964 70406966 70407039 70407054 70407123 70407232 70407314 70407359 70407643 70407762 70407772 70407784 70407823 70407993 70408190 70408244 70408309 70408436 70408894 70408922 70408931 70409525 70409534 70409574 70409688 70409752 70409818 70409826 70410184 70410273 70411070 70411081 70411132 70411395 70412096 70412356 70412437 70412606 70412912 70413031 70413032 70413192 70413508 70413777 70413804 70413905 70414029 70414599 70414640 70414689 70414796 70414930 70414931 70415364 70415522 70415549 70415655 70415744 70415787 70415812 70415877 70415904 70415954 70416013 70416121 70416155 70416321 70416464 70416480 70416486 70416526 70416542 70416600 70416785 70417188 70417215 70417286 70417394 70417495 70417646 70417733 70418085 70418179 70418277 70418279 70418312 70418313 70418395 70418471 70418493 70418650 70418723 70418849 70419361 70419420 70419458 70419473 70419567 70419588 70419884 70419913 70420001 70420426 70420487 70420547 70420639 70420812 70420888 70420900 70421022 70421080 70421227 70421307 70421369 70421486 70421528 70421601 70421618 70421675 70421689 70421771 70421938 70421986 70422100 70422141 70422199 70422296 70422344 70422704 70422727 70422745 70422851 70422890 70422923 70423008 70423014 70423051 70423236 70423325 70423580 70423633 70424215 70424226 70424811 70425058 70425151 70425165 70425203 70425220 70425296 70425337 70425430 70425434 70425826 70426071 70426147 70426383 70426388 70426795 70426866 70426973 70427362 70427554 70427571 70427607 70427623 70427653 70427888 70427947 70428039 70428058 70428064 70428558 70428599 70428840 70428874 70429211 70429409 70429421 70429565 70429748 70429812 70429896 70430041 70430155 70430536 70430879 70430909 70430979 70431063 70431070 70431130 70431261 70431410 70431476 70431505 70431589 70432006 70363728(i) 70370397(i) 70379727(i) 70389268(i) 70389833(i) 70390102(i) 70391979(i) 70397714(i) 70397823(i) 70400235(i) 70400524(i) 70401303(i) 70401741(i) 70402217(i) 70403954(i) 70403982(i) 70403989(i) 70404913(i) 70406212(i) 70406234(i) 70406255(i) 70407786(i) 70411912(i) 70413952(i) 70413961(i) 70414468(i) 70417253(i) 70417543(i) 70418956(i) 70419034(i) 70419720(i) 70421898(i) 70425358(i) 70427192(i) 70427272(i) 70428224(i) 70428533(i) 70430184(i) 70430233(i) 70430545(i) 70430557(i) 70431614(i) |
| JPT (Japanese in Tokyo, Japan) | 18% | 494 | 70362123 70362561 70362834 70363362 70366492 70367102 70367399 70368202 70368400 70368565 70370643 70370804 70371555 70371656 70371657 70372046 70372641 70372905 70372955 70374584 70374588 70375035 70375691 70376112 70376691 70376963 70377360 70377361 70377394 70377396 70377402 70377524 70377642 70378396 70378440 70378555 70379374 70379682 70380660 70380675 70381259 70381351 70382077 70382444 70382578 70382579 70382957 70383322 70383625 70383913 70383965 70383991 70384123 70384324 70385252 70385551 70385552 70385588 70385589 70386429 70386623 70386791 70387066 70387375 70387402 70387713 70388194 70388363 70388497 70388537 70388610 70388617 70388623 70388633 70388646 70388704 70388958 70389194 70389266 70389349 70389486 70389716 70389823 70389868 70389916 70389917 70389934 70389935 70390000 70390024 70390051 70390069 70390098 70390135 70390151 70390175 70390221 70390272 70390273 70390281 70390302 70390358 70390449 70390494 70390525 70390528 70390554 70390723 70390777 70390795 70391246 70391344 70391429 70391454 70391536 70391561 70391616 70391655 70391688 70391888 70391986 70392053 70392062 70392874 70392967 70393390 70393590 70393660 70393665 70393680 70393780 70394123 70394240 70394493 70394500 70394564 70394991 70395188 70395338 70395353 70395596 70395767 70395803 70395821 70395945 70396032 70396060 70396093 70396137 70396352 70396415 70396562 70396681 70396710 70397007 70397202 70397236 70397237 70397266 70397274 70397323 70397408 70397424 70397507 70397849 70397899 70397977 70398467 70398606 70398649 70398769 70398783 70398857 70399017 70399573 70399615 70399643 70399706 70399774 70399802 70399877 70399972 70400027 70400339 70400391 70400439 70400450 70400462 70400608 70400631 70400843 70400884 70400905 70401025 70401093 70401103 70401279 70401513 70401731 70401751 70401818 70401824 70401832 70401931 70402005 70402042 70402181 70402246 70402312 70402805 70402881 70402954 70402958 70403070 70403427 70403460 70403503 70403702 70403764 70403815 70404001 70404130 70404144 70404150 70404196 70404759 70404889 70404918 70404922 70405360 70405404 70405453 70405545 70405551 70405837 70405916 70405973 70406203 70406423 70406450 70406575 70406601 70406851 70406908 70406918 70406939 70406957 70406964 70406966 70407039 70407054 70407122 70407123 70407232 70407314 70407359 70407569 70407643 70407762 70407772 70407784 70407823 70407993 70408190 70408244 70408309 70408436 70408894 70408922 70408931 70409574 70409688 70409752 70409818 70409826 70409924 70410184 70410273 70411070 70411081 70411132 70411395 70411537 70412096 70412356 70412437 70412606 70412912 70413031 70413032 70413192 70413508 70413777 70413804 70413905 70414029 70414239 70414599 70414640 70414689 70414796 70414930 70414931 70415364 70415522 70415549 70415655 70415744 70415787 70415812 70415877 70415904 70415954 70416013 70416121 70416155 70416321 70416464 70416480 70416486 70416526 70416542 70416600 70416785 70417188 70417215 70417286 70417394 70417495 70417646 70417733 70418085 70418179 70418277 70418279 70418312 70418313 70418395 70418471 70418493 70418650 70418723 70418849 70419361 70419420 70419458 70419473 70419567 70419588 70419884 70419913 70420001 70420008 70420426 70420487 70420547 70420639 70420812 70420888 70420900 70421022 70421080 70421227 70421307 70421369 70421486 70421528 70421601 70421618 70421675 70421689 70421771 70421938 70421986 70422100 70422141 70422199 70422296 70422344 70422704 70422727 70422745 70422800 70422851 70422890 70422923 70423008 70423014 70423051 70423236 70423325 70423580 70423633 70424215 70424226 70424811 70425058 70425151 70425165 70425203 70425220 70425296 70425337 70425430 70425434 70425826 70426071 70426147 70426383 70426388 70426795 70426866 70426973 70427362 70427554 70427571 70427607 70427623 70427653 70427888 70427947 70428039 70428058 70428064 70428558 70428599 70428652 70428840 70428874 70429211 70429409 70429421 70429565 70429748 70429812 70429896 70430041 70430155 70430536 70430879 70430909 70430979 70431063 70431070 70431130 70431261 70431410 70431476 70431505 70431589 70431781 70432006 70363728(i) 70370397(i) 70379727(i) 70389268(i) 70389833(i) 70390102(i) 70391979(i) 70397714(i) 70397823(i) 70400235(i) 70400524(i) 70401303(i) 70401741(i) 70402217(i) 70403954(i) 70403982(i) 70403989(i) 70404913(i) 70406212(i) 70406234(i) 70406255(i) 70407786(i) 70411912(i) 70413952(i) 70413961(i) 70414468(i) 70417253(i) 70417543(i) 70418956(i) 70419034(i) 70419720(i) 70421898(i) 70425358(i) 70427192(i) 70427272(i) 70428224(i) 70428533(i) 70430184(i) 70430233(i) 70430545(i) 70430557(i) 70431614(i) |
| KHV (Kinh in Ho Chi Minh City, Vietnam) | 28% | 467 | 70361741 70362123 70366324 70366328 70366492 70367399 70372905 70372955 70374584 70374588 70375691 70376112 70376705 70376708 70376963 70377360 70377361 70377394 70377396 70377402 70377524 70377642 70378396 70378440 70378555 70379374 70379682 70380660 70380675 70381259 70381351 70382444 70382578 70382579 70383322 70383913 70383965 70383991 70384123 70384324 70385252 70385551 70385552 70385588 70385589 70386429 70386623 70386791 70387066 70387375 70388194 70388363 70388497 70388537 70388610 70388617 70388623 70388633 70388646 70388704 70388958 70389194 70389266 70389349 70389486 70389716 70389823 70389868 70389916 70389917 70389934 70389935 70390000 70390024 70390051 70390069 70390098 70390135 70390151 70390175 70390221 70390272 70390273 70390281 70390302 70390358 70390449 70390494 70390525 70390528 70390554 70390723 70390777 70390795 70391246 70391344 70391429 70391454 70391536 70391561 70391616 70391655 70391688 70391888 70391986 70392053 70392062 70392874 70392967 70393590 70393660 70393665 70393680 70393780 70394123 70394240 70394493 70394500 70394564 70394991 70395188 70395338 70395353 70395596 70395767 70395803 70395821 70395945 70396060 70396093 70396137 70396352 70396415 70396562 70396681 70396710 70397007 70397202 70397236 70397237 70397266 70397274 70397323 70397408 70397424 70397507 70397849 70397899 70397977 70398467 70398606 70398769 70398783 70398857 70399017 70399573 70399615 70399643 70399706 70399774 70399802 70399877 70399972 70400027 70400339 70400391 70400439 70400450 70400462 70400631 70400843 70400884 70400905 70401025 70401093 70401103 70401279 70401513 70401731 70401751 70401818 70401824 70401832 70401931 70402005 70402042 70402181 70402246 70402312 70402805 70402954 70402958 70403070 70403427 70403460 70403503 70403702 70403764 70403815 70404001 70404130 70404144 70404150 70404196 70404759 70404889 70404918 70404922 70405360 70405404 70405545 70405551 70405916 70405973 70406203 70406423 70406450 70406575 70406601 70406851 70406908 70406918 70406939 70406957 70406964 70406966 70407039 70407054 70407123 70407232 70407314 70407359 70407569 70407643 70407762 70407772 70407784 70407823 70407993 70408190 70408244 70408309 70408436 70408894 70408922 70408931 70409525 70409534 70409574 70409688 70409752 70409818 70409826 70410184 70410273 70411070 70411081 70411132 70411395 70412096 70412356 70412437 70412606 70412912 70413031 70413032 70413192 70413508 70413777 70413804 70413905 70414029 70414239 70414599 70414640 70414689 70414796 70414930 70414931 70415364 70415522 70415549 70415655 70415744 70415787 70415812 70415877 70415904 70415954 70416013 70416121 70416155 70416321 70416464 70416480 70416486 70416526 70416542 70416600 70416785 70417188 70417215 70417286 70417394 70417495 70417646 70417733 70418085 70418179 70418277 70418279 70418312 70418313 70418395 70418471 70418493 70418650 70418723 70418849 70419361 70419420 70419458 70419473 70419567 70419588 70419884 70419913 70420001 70420426 70420487 70420547 70420639 70420812 70420888 70420900 70421022 70421080 70421227 70421307 70421369 70421486 70421528 70421601 70421618 70421675 70421689 70421771 70421938 70421986 70422100 70422141 70422199 70422296 70422344 70422704 70422727 70422745 70422800 70422851 70422890 70422923 70423008 70423014 70423051 70423236 70423325 70423580 70423633 70424215 70424226 70424811 70425058 70425151 70425165 70425203 70425220 70425296 70425337 70425430 70425434 70425826 70426071 70426147 70426383 70426388 70426795 70426866 70426973 70427362 70427554 70427571 70427607 70427623 70427653 70427888 70427947 70428039 70428058 70428064 70428558 70428599 70428840 70428874 70429211 70429409 70429421 70429565 70429748 70429812 70429896 70430041 70430155 70430536 70430879 70430909 70430979 70431063 70431070 70431130 70431261 70431410 70431476 70431505 70431589 70432006 70363728(i) 70370397(i) 70379727(i) 70389268(i) 70389833(i) 70390102(i) 70391979(i) 70397714(i) 70397823(i) 70400235(i) 70400524(i) 70401303(i) 70401741(i) 70402217(i) 70403954(i) 70403982(i) 70403989(i) 70404913(i) 70406212(i) 70406234(i) 70406255(i) 70407786(i) 70411912(i) 70413952(i) 70413961(i) 70414468(i) 70417253(i) 70417543(i) 70418956(i) 70419034(i) 70419720(i) 70421898(i) 70425358(i) 70427192(i) 70427272(i) 70428224(i) 70428533(i) 70430184(i) 70430233(i) 70430545(i) 70430557(i) 70431614(i) |
| LWK (Luhya in Webuye, Kenya) | 39% | 396 | 70376705 70376708 70376963 70377360 70377361 70377394 70377396 70377402 70377524 70377642 70378555 70379374 70381259 70382444 70382578 70382579 70383965 70383991 70385252 70385552 70385588 70385589 70386623 70386791 70387066 70387375 70388363 70388497 70388537 70388610 70388617 70388623 70388633 70388704 70388958 70389194 70389266 70389349 70389486 70389823 70389868 70389916 70389917 70389934 70389935 70390000 70390024 70390051 70390069 70390098 70390135 70390151 70390175 70390221 70390272 70390273 70390281 70390302 70390358 70390449 70390494 70390528 70390554 70390723 70390777 70390795 70391246 70391344 70391429 70391454 70391536 70391561 70391616 70391655 70391688 70391986 70392053 70392062 70392874 70392967 70393660 70393665 70393680 70393780 70394240 70394493 70394500 70394564 70394991 70395188 70395338 70395596 70395767 70395803 70395821 70396060 70396093 70396137 70396352 70396415 70396562 70396681 70396710 70397007 70397202 70397236 70397237 70397266 70397274 70397323 70397408 70397424 70397507 70397849 70397899 70398467 70398606 70398769 70398857 70399017 70399573 70399615 70399706 70399774 70399877 70399972 70400027 70400339 70400391 70400450 70400462 70400631 70400843 70400884 70400905 70401025 70401093 70401103 70401279 70401513 70401731 70401751 70401818 70401824 70401832 70401931 70402005 70402042 70402181 70402246 70402312 70402805 70402954 70402958 70403070 70403427 70403460 70403503 70403702 70403764 70403815 70404001 70404130 70404144 70404150 70404196 70404759 70404889 70404918 70404922 70405360 70405404 70405545 70405551 70405916 70405973 70406203 70406423 70406450 70406601 70406851 70406908 70406918 70406939 70406964 70406966 70407039 70407054 70407123 70407232 70407359 70407643 70407762 70407772 70407784 70407823 70407993 70408190 70408309 70408436 70408894 70408922 70408931 70409574 70409752 70409818 70409826 70410184 70410273 70411070 70411081 70411132 70411395 70412096 70412356 70412437 70412606 70412912 70413031 70413032 70413192 70413508 70413804 70413905 70414029 70414599 70414640 70414689 70414796 70414930 70414931 70415364 70415522 70415549 70415655 70415744 70415787 70415812 70415877 70415904 70415954 70416013 70416155 70416321 70416464 70416480 70416486 70416526 70416542 70416600 70416785 70417188 70417215 70417286 70417394 70417495 70417646 70417733 70418085 70418179 70418277 70418279 70418312 70418313 70418395 70418471 70418493 70418650 70418723 70418849 70419361 70419420 70419458 70419473 70419567 70419588 70419884 70419913 70420426 70420487 70420547 70420639 70420812 70420888 70421022 70421080 70421227 70421307 70421369 70421601 70421618 70421675 70421689 70421771 70421938 70421986 70422100 70422141 70422199 70422344 70422745 70422851 70422923 70423008 70423014 70423051 70423236 70423325 70423580 70423633 70424215 70424226 70424811 70425058 70425151 70425165 70425203 70425220 70425296 70425337 70425430 70425434 70425826 70426147 70426383 70426388 70426795 70426866 70427362 70427571 70427607 70427623 70427653 70427888 70427947 70428039 70428058 70428064 70428558 70428840 70428874 70429211 70429421 70429565 70429748 70429812 70429896 70430041 70430155 70430536 70430879 70430909 70430979 70431063 70431070 70431130 70431261 70431410 70431505 70431589 70379727(i) 70389268(i) 70389833(i) 70390102(i) 70397714(i) 70397823(i) 70400235(i) 70400524(i) 70401303(i) 70401741(i) 70403954(i) 70403982(i) 70403989(i) 70404913(i) 70406212(i) 70406234(i) 70406255(i) 70407786(i) 70411912(i) 70413952(i) 70413961(i) 70414468(i) 70417543(i) 70419034(i) 70419720(i) 70421898(i) 70425358(i) 70427192(i) 70427272(i) 70428224(i) 70428533(i) 70430184(i) 70430233(i) 70430545(i) 70430557(i) 70431614(i) |
| MSL (Mende in Sierra Leone) | 27% | 414 | 70361741 70362561 70362834 70363362 70367102 70368202 70368400 70368565 70370643 70371555 70371656 70371657 70372641 70375035 70376963 70377360 70377361 70377394 70377396 70377402 70377524 70377642 70378555 70379374 70381259 70382077 70382444 70382578 70382579 70383625 70383965 70383991 70385252 70385552 70385588 70385589 70386623 70386791 70387066 70387375 70387402 70388363 70388497 70388537 70388610 70388617 70388623 70388633 70388704 70388958 70389194 70389266 70389349 70389486 70389823 70389868 70389916 70389917 70389934 70389935 70390000 70390024 70390051 70390069 70390098 70390135 70390151 70390175 70390221 70390272 70390273 70390281 70390302 70390358 70390449 70390494 70390528 70390554 70390723 70390777 70390795 70391246 70391344 70391429 70391454 70391536 70391561 70391616 70391655 70391688 70391986 70392053 70392062 70392874 70392967 70393660 70393665 70393680 70393780 70394240 70394493 70394500 70394564 70394991 70395188 70395338 70395596 70395767 70395803 70395821 70396032 70396060 70396093 70396137 70396352 70396415 70396562 70396681 70396710 70397007 70397202 70397236 70397237 70397266 70397274 70397323 70397408 70397424 70397507 70397849 70397899 70398467 70398606 70398649 70398769 70398857 70399017 70399573 70399615 70399774 70399877 70399972 70400027 70400339 70400391 70400450 70400462 70400631 70400843 70400884 70400905 70401025 70401093 70401103 70401279 70401513 70401731 70401751 70401818 70401824 70401832 70401931 70402005 70402042 70402181 70402246 70402312 70402805 70402881 70402954 70402958 70403070 70403427 70403460 70403503 70403702 70403764 70403815 70404001 70404130 70404144 70404150 70404196 70404759 70404889 70404918 70404922 70405360 70405404 70405545 70405551 70405916 70405973 70406203 70406423 70406450 70406601 70406851 70406908 70406918 70406939 70406964 70406966 70407039 70407054 70407122 70407123 70407232 70407359 70407643 70407762 70407772 70407784 70407823 70407993 70408190 70408436 70408894 70409574 70409752 70409818 70409826 70410184 70410273 70411070 70411081 70411132 70411395 70412096 70412356 70412437 70412606 70412912 70413031 70413032 70413192 70413508 70413777 70413804 70413905 70414029 70414599 70414640 70414689 70414796 70414930 70414931 70415364 70415522 70415549 70415655 70415744 70415787 70415812 70415877 70415904 70415954 70416013 70416155 70416321 70416464 70416480 70416486 70416526 70416542 70416600 70416785 70417188 70417215 70417286 70417394 70417495 70417646 70417733 70418085 70418179 70418277 70418279 70418312 70418313 70418395 70418471 70418493 70418650 70418723 70418849 70419361 70419420 70419458 70419473 70419567 70419588 70419884 70419913 70420426 70420487 70420547 70420639 70420812 70420888 70421022 70421080 70421227 70421307 70421369 70421601 70421618 70421675 70421689 70421771 70421938 70421986 70422100 70422141 70422199 70422296 70422344 70422704 70422727 70422745 70422851 70422923 70423008 70423014 70423051 70423236 70423325 70423580 70423633 70424215 70424226 70424811 70425058 70425151 70425165 70425203 70425220 70425296 70425337 70425430 70425434 70425826 70426147 70426383 70426388 70426795 70426866 70427362 70427571 70427607 70427623 70427653 70427888 70427947 70428039 70428058 70428064 70428558 70428840 70428874 70429211 70429421 70429565 70429748 70429812 70429896 70430041 70430155 70430536 70430879 70430909 70430979 70431063 70431070 70431130 70431261 70431410 70431505 70431589 70389268(i) 70389833(i) 70390102(i) 70397714(i) 70397823(i) 70400235(i) 70400524(i) 70401303(i) 70401741(i) 70403954(i) 70403982(i) 70403989(i) 70404913(i) 70406212(i) 70406234(i) 70406255(i) 70407786(i) 70411912(i) 70413952(i) 70413961(i) 70414468(i) 70417543(i) 70419034(i) 70419720(i) 70421898(i) 70425358(i) 70427192(i) 70427272(i) 70428224(i) 70428533(i) 70430184(i) 70430233(i) 70430545(i) 70430557(i) 70431614(i) |
| MXL (Mexican Ancestry from Los Angeles USA) | 38% | 456 | 70361741 70362123 70367399 70372905 70372955 70374584 70374588 70375691 70376112 70376705 70376708 70376963 70377360 70377361 70377394 70377396 70377402 70377524 70377642 70378396 70378440 70378555 70379374 70379682 70380660 70380675 70381259 70381351 70382444 70382578 70382579 70383322 70383913 70383965 70383991 70384123 70384324 70385252 70385552 70385588 70385589 70386429 70386623 70386791 70387066 70387375 70388363 70388497 70388537 70388610 70388617 70388623 70388633 70388646 70388704 70388958 70389194 70389266 70389349 70389486 70389716 70389823 70389868 70389916 70389917 70389934 70389935 70390000 70390024 70390051 70390069 70390098 70390135 70390151 70390175 70390221 70390272 70390273 70390281 70390302 70390358 70390449 70390494 70390525 70390528 70390554 70390723 70390777 70390795 70391246 70391344 70391429 70391454 70391536 70391561 70391616 70391655 70391688 70391888 70391986 70392053 70392062 70392874 70392967 70393590 70393660 70393665 70393680 70393780 70394240 70394493 70394500 70394564 70394991 70395188 70395338 70395353 70395596 70395767 70395803 70395821 70395945 70396060 70396093 70396137 70396352 70396415 70396562 70396681 70396710 70397007 70397202 70397236 70397237 70397266 70397274 70397323 70397408 70397424 70397507 70397849 70397899 70397977 70398467 70398606 70398769 70398783 70398857 70399017 70399573 70399615 70399643 70399706 70399774 70399802 70399877 70399972 70400027 70400339 70400391 70400439 70400450 70400462 70400631 70400843 70400884 70400905 70401025 70401093 70401103 70401279 70401513 70401731 70401751 70401818 70401824 70401832 70401931 70402005 70402042 70402181 70402246 70402312 70402805 70402954 70402958 70403070 70403427 70403460 70403503 70403702 70403764 70403815 70404001 70404130 70404144 70404150 70404196 70404759 70404889 70404918 70404922 70405360 70405404 70405545 70405551 70405916 70405973 70406203 70406423 70406450 70406575 70406601 70406851 70406908 70406918 70406939 70406957 70406964 70406966 70407039 70407054 70407123 70407232 70407314 70407359 70407643 70407762 70407772 70407784 70407823 70407993 70408190 70408244 70408309 70408436 70408894 70408922 70408931 70409574 70409688 70409752 70409818 70409826 70410184 70410273 70411070 70411081 70411132 70411395 70412096 70412356 70412437 70412606 70412912 70413031 70413032 70413192 70413508 70413777 70413804 70413905 70414029 70414599 70414640 70414689 70414796 70414930 70414931 70415364 70415522 70415549 70415655 70415744 70415787 70415812 70415877 70415904 70415954 70416013 70416121 70416155 70416321 70416464 70416480 70416486 70416526 70416542 70416600 70416785 70417188 70417215 70417286 70417394 70417495 70417646 70417733 70418085 70418179 70418277 70418279 70418312 70418313 70418395 70418471 70418493 70418650 70418723 70418849 70419361 70419420 70419458 70419473 70419567 70419588 70419884 70419913 70420001 70420426 70420487 70420547 70420639 70420812 70420888 70420900 70421022 70421080 70421227 70421307 70421369 70421486 70421528 70421601 70421618 70421675 70421689 70421771 70421938 70421986 70422100 70422141 70422199 70422296 70422344 70422704 70422727 70422745 70422851 70422890 70422923 70423008 70423014 70423051 70423236 70423325 70423580 70423633 70424215 70424226 70424811 70425058 70425151 70425165 70425203 70425220 70425296 70425337 70425430 70425434 70425826 70426071 70426147 70426383 70426388 70426795 70426866 70426973 70427362 70427554 70427571 70427607 70427623 70427653 70427888 70427947 70428039 70428058 70428064 70428558 70428599 70428840 70428874 70429211 70429409 70429421 70429565 70429748 70429812 70429896 70430041 70430155 70430536 70430879 70430909 70430979 70431063 70431070 70431130 70431261 70431410 70431476 70431505 70431589 70432006 70363728(i) 70370397(i) 70379727(i) 70389268(i) 70389833(i) 70390102(i) 70391979(i) 70397714(i) 70397823(i) 70400235(i) 70400524(i) 70401303(i) 70401741(i) 70402217(i) 70403954(i) 70403982(i) 70403989(i) 70404913(i) 70406212(i) 70406234(i) 70406255(i) 70407786(i) 70411912(i) 70413952(i) 70413961(i) 70414468(i) 70417253(i) 70417543(i) 70418956(i) 70419034(i) 70419720(i) 70421898(i) 70425358(i) 70427192(i) 70427272(i) 70428224(i) 70428533(i) 70430184(i) 70430233(i) 70430545(i) 70430557(i) 70431614(i) |
| PEL (Peruvians from Lima, Peru) | 32% | 457 | 70361741 70362123 70367399 70372905 70372955 70374584 70374588 70375691 70376112 70376705 70376708 70376963 70377360 70377361 70377394 70377396 70377402 70377524 70377642 70378396 70378440 70378555 70379374 70379682 70380660 70380675 70381259 70381351 70382444 70382578 70382579 70383322 70383913 70383965 70383991 70384123 70384324 70385252 70385552 70385588 70385589 70386429 70386623 70386791 70387066 70387375 70388363 70388497 70388537 70388610 70388617 70388623 70388633 70388646 70388704 70388958 70389194 70389266 70389349 70389486 70389716 70389823 70389868 70389916 70389917 70389934 70389935 70390000 70390024 70390051 70390069 70390098 70390135 70390151 70390175 70390221 70390272 70390273 70390281 70390302 70390358 70390449 70390494 70390525 70390528 70390554 70390723 70390777 70390795 70391246 70391344 70391429 70391454 70391536 70391561 70391616 70391655 70391688 70391888 70391986 70392053 70392062 70392874 70392967 70393590 70393660 70393665 70393680 70393780 70394240 70394493 70394500 70394564 70394991 70395188 70395338 70395353 70395596 70395767 70395803 70395821 70395945 70396060 70396093 70396137 70396352 70396415 70396562 70396681 70396710 70397007 70397202 70397236 70397237 70397266 70397274 70397323 70397408 70397424 70397507 70397849 70397899 70397977 70398467 70398606 70398769 70398783 70398857 70399017 70399573 70399615 70399643 70399706 70399774 70399802 70399877 70399972 70400027 70400339 70400391 70400439 70400450 70400462 70400631 70400843 70400884 70400905 70401025 70401093 70401103 70401279 70401513 70401731 70401751 70401818 70401824 70401832 70401931 70402005 70402042 70402181 70402246 70402312 70402805 70402954 70402958 70403070 70403427 70403460 70403503 70403702 70403764 70403815 70404001 70404130 70404144 70404150 70404196 70404759 70404889 70404918 70404922 70405360 70405404 70405545 70405551 70405916 70405973 70406203 70406423 70406450 70406575 70406601 70406851 70406908 70406918 70406939 70406957 70406964 70406966 70407039 70407054 70407123 70407232 70407314 70407359 70407643 70407762 70407772 70407784 70407823 70407993 70408190 70408244 70408309 70408436 70408894 70408922 70408931 70409574 70409688 70409752 70409818 70409826 70410184 70410273 70411070 70411081 70411132 70411395 70412096 70412356 70412437 70412606 70412912 70413031 70413032 70413192 70413508 70413777 70413804 70413905 70414029 70414599 70414640 70414689 70414796 70414930 70414931 70415364 70415522 70415549 70415655 70415744 70415787 70415812 70415877 70415904 70415954 70416013 70416121 70416155 70416321 70416464 70416480 70416486 70416526 70416542 70416600 70416785 70417188 70417215 70417286 70417394 70417495 70417646 70417733 70418085 70418179 70418277 70418279 70418312 70418313 70418395 70418471 70418493 70418650 70418723 70418849 70419361 70419420 70419458 70419473 70419567 70419588 70419884 70419913 70420001 70420426 70420487 70420547 70420639 70420812 70420888 70420900 70421022 70421080 70421227 70421307 70421369 70421486 70421528 70421601 70421618 70421675 70421689 70421771 70421938 70421986 70422100 70422141 70422199 70422296 70422344 70422704 70422727 70422745 70422851 70422890 70422923 70423008 70423014 70423051 70423236 70423325 70423580 70423633 70424215 70424226 70424811 70425058 70425151 70425165 70425203 70425220 70425296 70425337 70425430 70425434 70425826 70426071 70426147 70426383 70426388 70426795 70426866 70426973 70427362 70427554 70427571 70427607 70427623 70427653 70427888 70427947 70428039 70428058 70428064 70428558 70428599 70428840 70428874 70429211 70429409 70429421 70429565 70429748 70429812 70429896 70430041 70430155 70430536 70430879 70430909 70430979 70431063 70431070 70431130 70431261 70431410 70431476 70431505 70431589 70432006 70363728(i) 70364222(i) 70370397(i) 70379727(i) 70389268(i) 70389833(i) 70390102(i) 70391979(i) 70397714(i) 70397823(i) 70400235(i) 70400524(i) 70401303(i) 70401741(i) 70402217(i) 70403954(i) 70403982(i) 70403989(i) 70404913(i) 70406212(i) 70406234(i) 70406255(i) 70407786(i) 70411912(i) 70413952(i) 70413961(i) 70414468(i) 70417253(i) 70417543(i) 70418956(i) 70419034(i) 70419720(i) 70421898(i) 70425358(i) 70427192(i) 70427272(i) 70428224(i) 70428533(i) 70430184(i) 70430233(i) 70430545(i) 70430557(i) 70431614(i) |
| PJL (Punjabi from Lahore, Pakistan) | 36% | 455 | 70361741 70362123 70367399 70372905 70372955 70374584 70374588 70375691 70376112 70376963 70377360 70377361 70377394 70377396 70377402 70377524 70377642 70378396 70378440 70378555 70379374 70379682 70380660 70380675 70381259 70381351 70382444 70382578 70382579 70383322 70383913 70383965 70383991 70384123 70384324 70385252 70385552 70385588 70385589 70386429 70386623 70386791 70387066 70387375 70388363 70388497 70388537 70388610 70388617 70388623 70388633 70388646 70388704 70388958 70389194 70389266 70389349 70389486 70389716 70389823 70389868 70389916 70389917 70389934 70389935 70390000 70390024 70390051 70390069 70390098 70390135 70390151 70390175 70390221 70390272 70390273 70390281 70390302 70390358 70390449 70390494 70390525 70390528 70390554 70390723 70390777 70390795 70391246 70391344 70391429 70391454 70391536 70391561 70391616 70391655 70391688 70391888 70391986 70392053 70392062 70392967 70393590 70393660 70393665 70393680 70393780 70394240 70394493 70394500 70394564 70394991 70395188 70395338 70395353 70395596 70395767 70395803 70395821 70395945 70396060 70396093 70396137 70396352 70396415 70396562 70396681 70396710 70397007 70397202 70397236 70397237 70397266 70397274 70397323 70397408 70397424 70397507 70397849 70397899 70397977 70398467 70398606 70398769 70398783 70398857 70399017 70399573 70399615 70399643 70399706 70399774 70399802 70399877 70399972 70400027 70400339 70400391 70400439 70400450 70400462 70400631 70400843 70400884 70400905 70401025 70401093 70401103 70401279 70401513 70401731 70401751 70401818 70401824 70401832 70401931 70402005 70402042 70402181 70402246 70402312 70402805 70402954 70402958 70403070 70403427 70403460 70403503 70403702 70403764 70403815 70404001 70404130 70404144 70404150 70404196 70404759 70404889 70404918 70404922 70405360 70405404 70405545 70405551 70405916 70405973 70406203 70406423 70406450 70406575 70406601 70406851 70406908 70406918 70406939 70406957 70406964 70406966 70407039 70407054 70407123 70407232 70407314 70407359 70407643 70407762 70407772 70407784 70407823 70407993 70408190 70408244 70408309 70408436 70408894 70408922 70408931 70409525 70409534 70409574 70409688 70409752 70409818 70409826 70410184 70410273 70411070 70411081 70411132 70411395 70412096 70412356 70412437 70412606 70412912 70413031 70413032 70413192 70413508 70413777 70413804 70413905 70414029 70414599 70414640 70414689 70414796 70414930 70414931 70415364 70415522 70415549 70415655 70415744 70415787 70415812 70415877 70415904 70415954 70416013 70416121 70416155 70416321 70416464 70416480 70416486 70416526 70416542 70416600 70416785 70417188 70417215 70417286 70417394 70417495 70417646 70417733 70418085 70418179 70418277 70418279 70418312 70418313 70418395 70418471 70418493 70418650 70418723 70418849 70419361 70419420 70419458 70419473 70419567 70419588 70419884 70419913 70420001 70420426 70420487 70420547 70420639 70420812 70420888 70420900 70421022 70421080 70421227 70421307 70421369 70421486 70421528 70421601 70421618 70421675 70421689 70421771 70421938 70421986 70422100 70422141 70422199 70422296 70422344 70422704 70422727 70422745 70422851 70422890 70422923 70423008 70423014 70423051 70423236 70423325 70423580 70423633 70424215 70424226 70424811 70425058 70425151 70425165 70425203 70425220 70425296 70425337 70425430 70425434 70425826 70426071 70426147 70426383 70426388 70426795 70426866 70426973 70427362 70427554 70427571 70427607 70427623 70427653 70427888 70427947 70428039 70428058 70428064 70428558 70428599 70428840 70428874 70429211 70429409 70429421 70429565 70429748 70429812 70429896 70430041 70430155 70430536 70430879 70430909 70430979 70431063 70431070 70431130 70431261 70431410 70431476 70431505 70431589 70432006 70363728(i) 70370397(i) 70379727(i) 70389268(i) 70389833(i) 70390102(i) 70391979(i) 70397714(i) 70397823(i) 70400235(i) 70400524(i) 70401303(i) 70401741(i) 70402217(i) 70403954(i) 70403982(i) 70403989(i) 70404913(i) 70406212(i) 70406234(i) 70406255(i) 70407786(i) 70411912(i) 70413952(i) 70413961(i) 70414468(i) 70417253(i) 70417543(i) 70418956(i) 70419034(i) 70419720(i) 70421898(i) 70425358(i) 70427192(i) 70427272(i) 70428224(i) 70428533(i) 70430184(i) 70430233(i) 70430545(i) 70430557(i) 70431614(i) |
| PUR (Puerto Ricans from Puerto Rico) | 31% | 456 | 70361741 70362123 70367399 70372905 70372955 70374584 70374588 70375691 70376112 70376963 70377360 70377361 70377394 70377396 70377402 70377524 70377642 70378396 70378440 70378555 70379374 70379682 70380660 70380675 70381259 70381351 70382444 70382578 70382579 70383322 70383913 70383965 70383991 70384123 70384324 70385252 70385552 70385588 70385589 70386429 70386623 70386791 70387066 70387375 70388363 70388497 70388537 70388610 70388617 70388623 70388633 70388646 70388704 70388958 70389194 70389266 70389349 70389486 70389716 70389823 70389868 70389916 70389917 70389934 70389935 70390000 70390024 70390051 70390069 70390098 70390135 70390151 70390175 70390221 70390272 70390273 70390281 70390302 70390358 70390449 70390494 70390525 70390528 70390554 70390723 70390777 70390795 70391246 70391344 70391429 70391454 70391536 70391561 70391616 70391655 70391688 70391888 70391986 70392053 70392062 70392874 70392967 70393590 70393660 70393665 70393680 70393780 70394240 70394493 70394500 70394564 70394991 70395188 70395338 70395353 70395596 70395767 70395803 70395821 70395945 70396060 70396093 70396137 70396352 70396415 70396562 70396681 70396710 70397007 70397202 70397236 70397237 70397266 70397274 70397323 70397408 70397424 70397507 70397849 70397899 70397977 70398467 70398606 70398769 70398783 70398857 70399017 70399573 70399615 70399643 70399706 70399774 70399802 70399877 70399972 70400027 70400339 70400391 70400439 70400450 70400462 70400631 70400843 70400884 70400905 70401025 70401093 70401103 70401279 70401513 70401731 70401751 70401818 70401824 70401832 70401931 70402005 70402042 70402181 70402246 70402312 70402805 70402954 70402958 70403070 70403427 70403460 70403503 70403702 70403764 70403815 70404001 70404130 70404144 70404150 70404196 70404759 70404889 70404918 70404922 70405360 70405404 70405545 70405551 70405916 70405973 70406203 70406423 70406450 70406575 70406601 70406851 70406908 70406918 70406939 70406957 70406964 70406966 70407039 70407054 70407123 70407232 70407314 70407359 70407643 70407762 70407772 70407784 70407823 70407993 70408190 70408244 70408309 70408436 70408894 70408922 70408931 70409525 70409534 70409574 70409688 70409752 70409818 70409826 70410184 70410273 70411070 70411081 70411132 70411395 70412096 70412356 70412437 70412606 70412912 70413031 70413032 70413192 70413508 70413777 70413804 70413905 70414029 70414599 70414640 70414689 70414796 70414930 70414931 70415364 70415522 70415549 70415655 70415744 70415787 70415812 70415877 70415904 70415954 70416013 70416121 70416155 70416321 70416464 70416480 70416486 70416526 70416542 70416600 70416785 70417188 70417215 70417286 70417394 70417495 70417646 70417733 70418085 70418179 70418277 70418279 70418312 70418313 70418395 70418471 70418493 70418650 70418723 70418849 70419361 70419420 70419458 70419473 70419567 70419588 70419884 70419913 70420001 70420426 70420487 70420547 70420639 70420812 70420888 70420900 70421022 70421080 70421227 70421307 70421369 70421486 70421528 70421601 70421618 70421675 70421689 70421771 70421938 70421986 70422100 70422141 70422199 70422296 70422344 70422704 70422727 70422745 70422851 70422890 70422923 70423008 70423014 70423051 70423236 70423325 70423580 70423633 70424215 70424226 70424811 70425058 70425151 70425165 70425203 70425220 70425296 70425337 70425430 70425434 70425826 70426071 70426147 70426383 70426388 70426795 70426866 70426973 70427362 70427554 70427571 70427607 70427623 70427653 70427888 70427947 70428039 70428058 70428064 70428558 70428599 70428840 70428874 70429211 70429409 70429421 70429565 70429748 70429812 70429896 70430041 70430155 70430536 70430879 70430909 70430979 70431063 70431070 70431130 70431261 70431410 70431476 70431505 70431589 70432006 70363728(i) 70370397(i) 70379727(i) 70389268(i) 70389833(i) 70390102(i) 70391979(i) 70397714(i) 70397823(i) 70400235(i) 70400524(i) 70401303(i) 70401741(i) 70402217(i) 70403954(i) 70403982(i) 70403989(i) 70404913(i) 70406212(i) 70406234(i) 70406255(i) 70407786(i) 70411912(i) 70413952(i) 70413961(i) 70414468(i) 70417253(i) 70417543(i) 70418956(i) 70419034(i) 70419720(i) 70421898(i) 70425358(i) 70427192(i) 70427272(i) 70428224(i) 70428533(i) 70430184(i) 70430233(i) 70430545(i) 70430557(i) 70431614(i) |
| STU (Sri Lankan Tamil from the UK) | 30% | 457 | 70361741 70362123 70367399 70372905 70372955 70374584 70374588 70375691 70376112 70376705 70376708 70376963 70377360 70377361 70377394 70377396 70377402 70377524 70377642 70378396 70378440 70378555 70379374 70379682 70380660 70380675 70381259 70381351 70382444 70382578 70382579 70383322 70383913 70383965 70383991 70384123 70384324 70385252 70385552 70385588 70385589 70386429 70386623 70386791 70387066 70387375 70388363 70388497 70388537 70388610 70388617 70388623 70388633 70388646 70388704 70388958 70389194 70389266 70389349 70389486 70389716 70389823 70389868 70389916 70389917 70389934 70389935 70390000 70390024 70390051 70390069 70390098 70390135 70390151 70390175 70390221 70390272 70390273 70390281 70390302 70390358 70390449 70390494 70390525 70390528 70390554 70390723 70390777 70390795 70391246 70391344 70391429 70391454 70391536 70391561 70391616 70391655 70391688 70391888 70391986 70392053 70392062 70392874 70392967 70393590 70393660 70393665 70393680 70393780 70394240 70394493 70394500 70394564 70394991 70395188 70395338 70395353 70395596 70395767 70395803 70395821 70395945 70396060 70396093 70396137 70396352 70396415 70396562 70396681 70396710 70397007 70397202 70397236 70397237 70397266 70397274 70397323 70397408 70397424 70397507 70397849 70397899 70397977 70398467 70398606 70398769 70398783 70398857 70399017 70399573 70399615 70399643 70399706 70399774 70399802 70399877 70399972 70400027 70400339 70400391 70400439 70400450 70400462 70400631 70400843 70400884 70400905 70401025 70401093 70401103 70401279 70401513 70401731 70401751 70401818 70401824 70401832 70401931 70402005 70402042 70402181 70402246 70402312 70402805 70402954 70402958 70403070 70403427 70403460 70403503 70403702 70403764 70403815 70404001 70404130 70404144 70404150 70404196 70404759 70404889 70404918 70404922 70405360 70405404 70405545 70405551 70405916 70405973 70406203 70406423 70406450 70406575 70406601 70406851 70406908 70406918 70406939 70406957 70406964 70406966 70407039 70407054 70407123 70407232 70407314 70407359 70407643 70407762 70407772 70407784 70407823 70407993 70408190 70408244 70408309 70408894 70408922 70408931 70409525 70409534 70409574 70409688 70409752 70409818 70409826 70410184 70410273 70411070 70411081 70411132 70411395 70412096 70412356 70412437 70412606 70412912 70413031 70413032 70413192 70413508 70413777 70413804 70413905 70414029 70414599 70414640 70414689 70414796 70414930 70414931 70415364 70415522 70415549 70415655 70415744 70415787 70415812 70415877 70415904 70415954 70416013 70416121 70416155 70416321 70416464 70416480 70416486 70416526 70416542 70416600 70416785 70417188 70417215 70417286 70417394 70417495 70417646 70417733 70418085 70418179 70418277 70418279 70418312 70418313 70418395 70418471 70418493 70418650 70418723 70418849 70419361 70419420 70419458 70419473 70419567 70419588 70419884 70419913 70420001 70420426 70420487 70420547 70420639 70420812 70420888 70420900 70421022 70421080 70421227 70421307 70421369 70421486 70421528 70421601 70421618 70421675 70421689 70421771 70421938 70421986 70422100 70422141 70422199 70422296 70422344 70422704 70422727 70422745 70422851 70422890 70422923 70423008 70423014 70423051 70423236 70423325 70423580 70423633 70424215 70424226 70424811 70425058 70425151 70425165 70425203 70425220 70425296 70425337 70425430 70425434 70425826 70426071 70426147 70426383 70426388 70426795 70426866 70426973 70427362 70427554 70427571 70427607 70427623 70427653 70427888 70427947 70428039 70428058 70428064 70428558 70428599 70428840 70428874 70429211 70429409 70429421 70429565 70429748 70429812 70429896 70430041 70430155 70430536 70430879 70430909 70430979 70431063 70431070 70431130 70431261 70431410 70431476 70431505 70431589 70432006 70363728(i) 70370397(i) 70379727(i) 70389268(i) 70389833(i) 70390102(i) 70391979(i) 70397714(i) 70397823(i) 70400235(i) 70400524(i) 70401303(i) 70401741(i) 70402217(i) 70403954(i) 70403982(i) 70403989(i) 70404913(i) 70406212(i) 70406234(i) 70406255(i) 70407786(i) 70411912(i) 70413952(i) 70413961(i) 70414468(i) 70417253(i) 70417543(i) 70418956(i) 70419034(i) 70419720(i) 70421898(i) 70425358(i) 70427192(i) 70427272(i) 70428224(i) 70428533(i) 70430184(i) 70430233(i) 70430545(i) 70430557(i) 70431614(i) |
| TSI (Toscani in Italia) | 32% | 454 | 70361741 70362123 70367399 70372905 70372955 70374584 70374588 70375691 70376112 70376963 70377360 70377361 70377394 70377396 70377402 70377524 70377642 70378396 70378440 70378555 70379374 70379682 70380660 70380675 70381259 70381351 70382444 70382578 70382579 70383322 70383913 70383965 70383991 70384123 70384324 70385252 70385552 70385588 70385589 70386429 70386623 70386791 70387066 70387375 70388363 70388497 70388537 70388610 70388617 70388623 70388633 70388646 70388704 70388958 70389194 70389266 70389349 70389486 70389716 70389823 70389868 70389916 70389917 70389934 70389935 70390000 70390024 70390051 70390069 70390098 70390135 70390151 70390175 70390221 70390272 70390273 70390281 70390302 70390358 70390449 70390494 70390525 70390528 70390554 70390723 70390777 70390795 70391246 70391344 70391429 70391454 70391536 70391561 70391616 70391655 70391688 70391888 70391986 70392053 70392062 70392874 70392967 70393590 70393660 70393665 70393680 70393780 70394240 70394493 70394500 70394564 70394991 70395188 70395338 70395353 70395596 70395767 70395803 70395821 70395945 70396060 70396093 70396137 70396352 70396415 70396562 70396681 70396710 70397007 70397202 70397236 70397237 70397266 70397274 70397323 70397408 70397424 70397507 70397849 70397899 70397977 70398467 70398606 70398769 70398783 70398857 70399017 70399573 70399615 70399643 70399706 70399774 70399802 70399877 70399972 70400027 70400339 70400391 70400439 70400450 70400462 70400631 70400843 70400884 70400905 70401025 70401093 70401103 70401279 70401513 70401731 70401751 70401818 70401824 70401832 70401931 70402005 70402042 70402181 70402246 70402312 70402805 70402954 70402958 70403070 70403427 70403460 70403503 70403702 70403764 70403815 70404001 70404130 70404144 70404150 70404196 70404759 70404889 70404918 70404922 70405360 70405404 70405545 70405551 70405916 70405973 70406203 70406423 70406450 70406575 70406601 70406851 70406908 70406918 70406939 70406957 70406964 70406966 70407039 70407054 70407123 70407232 70407314 70407359 70407643 70407762 70407772 70407784 70407823 70407993 70408190 70408244 70408309 70408436 70408894 70408922 70408931 70409574 70409688 70409752 70409818 70409826 70410184 70410273 70411070 70411081 70411132 70411395 70412096 70412356 70412437 70412606 70412912 70413031 70413032 70413192 70413508 70413777 70413804 70413905 70414029 70414599 70414640 70414689 70414796 70414930 70414931 70415364 70415522 70415549 70415655 70415744 70415787 70415812 70415877 70415904 70415954 70416013 70416121 70416155 70416321 70416464 70416480 70416486 70416526 70416542 70416600 70416785 70417188 70417215 70417286 70417394 70417495 70417646 70417733 70418085 70418179 70418277 70418279 70418312 70418313 70418395 70418471 70418493 70418650 70418723 70418849 70419361 70419420 70419458 70419473 70419567 70419588 70419884 70419913 70420001 70420426 70420487 70420547 70420639 70420812 70420888 70420900 70421022 70421080 70421227 70421307 70421369 70421486 70421528 70421601 70421618 70421675 70421689 70421771 70421938 70421986 70422100 70422141 70422199 70422296 70422344 70422704 70422727 70422745 70422851 70422890 70422923 70423008 70423014 70423051 70423236 70423325 70423580 70423633 70424215 70424226 70424811 70425058 70425151 70425165 70425203 70425220 70425296 70425337 70425430 70425434 70425826 70426071 70426147 70426383 70426388 70426795 70426866 70426973 70427362 70427554 70427571 70427607 70427623 70427653 70427888 70427947 70428039 70428058 70428064 70428558 70428599 70428840 70428874 70429211 70429409 70429421 70429565 70429748 70429812 70429896 70430041 70430155 70430536 70430879 70430909 70430979 70431063 70431070 70431130 70431261 70431410 70431476 70431505 70431589 70432006 70363728(i) 70370397(i) 70379727(i) 70389268(i) 70389833(i) 70390102(i) 70391979(i) 70397714(i) 70397823(i) 70400235(i) 70400524(i) 70401303(i) 70401741(i) 70402217(i) 70403954(i) 70403982(i) 70403989(i) 70404913(i) 70406212(i) 70406234(i) 70406255(i) 70407786(i) 70411912(i) 70413952(i) 70413961(i) 70414468(i) 70417253(i) 70417543(i) 70418956(i) 70419034(i) 70419720(i) 70421898(i) 70425358(i) 70427192(i) 70427272(i) 70428224(i) 70428533(i) 70430184(i) 70430233(i) 70430545(i) 70430557(i) 70431614(i) |
| YRI (Yoruba in Ibadan, Nigeria) | 32% | 399 | 70376705 70376708 70376963 70377360 70377361 70377394 70377396 70377402 70377524 70377642 70378555 70379374 70381259 70382444 70382578 70382579 70383965 70383991 70385252 70385552 70385588 70385589 70386623 70386791 70387066 70387375 70388363 70388497 70388537 70388610 70388617 70388623 70388633 70388704 70388958 70389194 70389266 70389349 70389486 70389823 70389868 70389916 70389917 70389934 70389935 70390000 70390024 70390051 70390069 70390098 70390135 70390151 70390175 70390221 70390272 70390273 70390281 70390302 70390358 70390449 70390494 70390528 70390554 70390723 70390777 70390795 70391246 70391344 70391429 70391454 70391536 70391561 70391616 70391655 70391688 70391986 70392053 70392062 70392874 70392967 70393660 70393665 70393680 70393780 70394240 70394493 70394500 70394564 70394991 70395188 70395338 70395596 70395767 70395803 70395821 70396060 70396093 70396137 70396352 70396415 70396562 70396681 70396710 70397007 70397202 70397236 70397237 70397266 70397274 70397323 70397408 70397424 70397507 70397849 70397899 70398467 70398606 70398769 70398857 70399017 70399573 70399615 70399774 70399877 70399972 70400027 70400339 70400391 70400450 70400462 70400631 70400843 70400884 70400905 70401025 70401093 70401103 70401279 70401513 70401731 70401751 70401818 70401824 70401832 70401931 70402005 70402042 70402181 70402246 70402312 70402805 70402954 70402958 70403070 70403427 70403460 70403503 70403702 70403764 70403815 70404001 70404130 70404144 70404150 70404196 70404759 70404889 70404918 70404922 70405360 70405404 70405545 70405551 70405916 70405973 70406203 70406423 70406450 70406601 70406851 70406908 70406918 70406939 70406964 70406966 70407039 70407054 70407123 70407232 70407359 70407643 70407762 70407772 70407784 70407823 70407993 70408190 70408309 70408436 70408894 70408922 70408931 70409574 70409752 70409818 70409826 70410184 70410273 70411070 70411081 70411132 70411395 70412096 70412356 70412437 70412606 70412912 70413031 70413032 70413192 70413508 70413777 70413804 70413905 70414029 70414599 70414640 70414689 70414796 70414930 70414931 70415364 70415522 70415549 70415655 70415744 70415787 70415812 70415877 70415904 70415954 70416013 70416155 70416321 70416464 70416480 70416486 70416526 70416542 70416600 70416785 70417188 70417215 70417286 70417394 70417495 70417646 70417733 70418085 70418179 70418277 70418279 70418312 70418313 70418395 70418471 70418493 70418650 70418723 70418849 70419361 70419420 70419458 70419473 70419567 70419588 70419884 70419913 70420426 70420487 70420547 70420639 70420812 70420888 70421022 70421080 70421227 70421307 70421369 70421601 70421618 70421675 70421689 70421771 70421938 70421986 70422100 70422141 70422199 70422296 70422344 70422704 70422727 70422745 70422851 70422923 70423008 70423014 70423051 70423236 70423325 70423580 70423633 70424215 70424226 70424811 70425058 70425151 70425165 70425203 70425220 70425296 70425337 70425430 70425434 70425826 70426147 70426383 70426388 70426795 70426866 70427362 70427571 70427607 70427623 70427653 70427888 70427947 70428039 70428058 70428064 70428558 70428840 70428874 70429211 70429421 70429565 70429748 70429812 70429896 70430041 70430155 70430536 70430879 70430909 70430979 70431063 70431070 70431130 70431261 70431410 70431505 70431589 70379727(i) 70389268(i) 70389833(i) 70390102(i) 70397714(i) 70397823(i) 70400235(i) 70400524(i) 70401303(i) 70401741(i) 70403954(i) 70403982(i) 70403989(i) 70404913(i) 70406212(i) 70406234(i) 70406255(i) 70407786(i) 70411912(i) 70413952(i) 70413961(i) 70414468(i) 70417543(i) 70419034(i) 70419720(i) 70421898(i) 70425358(i) 70427192(i) 70427272(i) 70428224(i) 70428533(i) 70430184(i) 70430233(i) 70430545(i) 70430557(i) 70431614(i) |

^a^ i denotes indel.

Supplementary Table 2. Allele composition for 454 genetic variations in CEU core haplotypes and genotype for corresponding sites in three Neanderthals and Denisovan.

| rsID | position in chr4 | Haplotype 1 | Haplotype 2 | Altai | Vindija | Chagyrskaya | Denisovan |
| --- | --- | --- | --- | --- | --- | --- | --- |
| rs941389 | 70361741 | A | C | C | - | - | A |
| rs13129471 | 70362123 | A | G | G | G | G | G |
| rs5859185 | 70363728 | G | GA | - | - | - | G |
| rs4694749 | 70367399 | C | T | T | T | T | T |
| rs35266867 | 70370397 | C | CA | - | - | - | C |
| rs4626103 | 70372905 | T | A | A | - | - | A |
| rs4339112 | 70372955 | T | C | C | - | - | C |
| rs9994075 | 70374584 | A | T | T | T | T | T |
| rs9991662 | 70374588 | C | A | A | A | A | A |
| rs2331750 | 70375691 | A | G | G | G | G | G |
| rs2082336 | 70376112 | C | T | T | - | - | T |
| rs56075349 | 70376963 | T | C | C | - | - | C |
| rs12651434 | 70377360 | C | T | T | T | T | T |
| rs12645210 | 70377361 | G | A | A | A | A | A |
| rs12645214 | 70377394 | G | A | A | A | A | A |
| rs12643133 | 70377396 | T | C | C | C | C | C |
| rs12642506 | 70377402 | A | G | G | G | G | G |
| rs12645242 | 70377524 | G | A | A | A | A | A |
| rs62306991 | 70377642 | C | T | T | T | T | T |
| rs1119991 | 70378396 | C | T | T | - | - | T |
| rs1988114 | 70378440 | G | T | T | - | - | T |
| rs28885673 | 70378555 | A | C | C | - | - | C |
| rs6852142 | 70379374 | T | C | C | - | - | C |
| rs3866920 | 70379682 | T | A | A | - | - | A |
| rs77344785 | 70379727 | AT | A | A | - | - | A |
| rs2010298 | 70380660 | A | G | G | G | G | G |
| rs2010295 | 70380675 | C | T | T | T | T | T |
| rs2116560 | 70381259 | C | T | T | - | - | T |
| rs2099286 | 70381351 | A | G | G | - | - | G |
| rs6836321 | 70382444 | T | C | C | C | C | C |
| rs6831509 | 70382578 | A | G | G | - | - | G |
| rs6855029 | 70382579 | C | G | G | - | - | G |
| rs2029781 | 70383322 | C | A | A | A | A | A |
| rs2018160 | 70383913 | T | G | G | G | G | G |
| rs6817627 | 70383965 | G | T | T | T | T | T |
| rs6844026 | 70383991 | A | G | G | G | G | G |
| rs1987482 | 70384123 | T | G | G | G | G | G |
| rs1432326 | 70384324 | C | T | T | T | T | T |
| rs9997934 | 70385252 | C | A | A | A | A | A |
| rs10020023 | 70385552 | G | T | T | T | T | T |
| rs1864835 | 70385588 | T | G | G | G | G | G |
| rs1864834 | 70385589 | G | A | A | A | A | A |
| rs2082334 | 70386429 | T | C | C | C | C | C |
| rs11725398 | 70386623 | C | A | A | A | A | A |
| rs11729679 | 70386791 | A | G | G | - | - | G |
| rs12643318 | 70387066 | C | G | G | G | G | G |
| rs6600779 | 70387375 | G | A | A | - | - | A |
| rs28712359 | 70388363 | A | G | G | G | G | G |
| rs28507292 | 70388497 | A | G | G | G | G | G |
| rs28633225 | 70388537 | G | A | A | - | - | A |
| rs28770265 | 70388610 | T | A | A | - | - | A |
| rs28771795 | 70388617 | C | T | T | - | - | T |
| rs12647404 | 70388623 | A | G | G | - | - | G |
| rs28799896 | 70388633 | C | G | G | G | G | G |
| rs1821188 | 70388646 | C | G | G | G | G | G |
| rs28776017 | 70388704 | A | G | G | - | - | G |
| rs11249444 | 70388958 | C | G | G | G | G | G |
| rs62307002 | 70389194 | A | G | G | G | G | G |
| rs62307003 | 70389266 | G | A | A | - | - | A |
| rs150970444 | 70389268 | A | AT | AT | - | - | AT |
| rs7678186 | 70389349 | T | C | C | C | C | C |
| rs7673670 | 70389486 | A | G | G | - | - | G |
| rs6836432 | 70389716 | G | T | T | T | T | T |
| rs7695287 | 70389823 | G | A | A | A | A | A |
| rs373962964 | 70389833 | A | ATTCT | - | - | - | A |
| rs7699910 | 70389868 | G | A | A | A | A | A |
| rs7654942 | 70389916 | C | T | T | T | T | T |
| rs7678971 | 70389917 | A | G | G | G | G | G |
| rs78076396 | 70389934 | C | T | T | - | - | T |
| rs72646768 | 70389935 | A | G | G | - | - | G |
| rs35368658 | 70390000 | C | T | T | T | T | T |
| rs36043662 | 70390024 | C | T | T | T | T | T |
| rs72646771 | 70390051 | C | T | T | T | T | T |
| rs35548726 | 70390069 | A | G | G | G | G | G |
| rs34681342 | 70390098 | C | T | T | T | T | T |
| rs145607456 | 70390102 | T | TAC | TAC | - | - | TAC |
| rs36086046 | 70390135 | T | C | C | C | C | C |
| rs72646774 | 70390151 | A | G | G | G | G | G |
| rs35311506 | 70390175 | T | C | C | C | C | C |
| rs1370884 | 70390221 | A | C | C | C | C | C |
| rs76722375 | 70390272 | A | G | G | G | G | G |
| rs76829484 | 70390273 | G | T | T | T | T | T |
| rs34461063 | 70390281 | G | A | A | A | A | A |
| rs35783289 | 70390302 | G | A | A | A | A | A |
| rs34545840 | 70390358 | G | T | G | G | G | T |
| rs35449147 | 70390449 | G | A | A | - | - | A |
| rs34291474 | 70390494 | C | T | T | T | T | T |
| rs1368115 | 70390525 | A | G | G | G | G | G |
| rs34643431 | 70390528 | C | G | G | G | G | G |
| rs17671506 | 70390554 | T | C | C | - | - | C |
| rs17615130 | 70390723 | A | G | G | G | G | G |
| rs66862535 | 70390777 | A | G | G | G | G | G |
| rs72646780 | 70390795 | C | T | T | T | T | T |
| rs10011713 | 70391246 | A | C | C | C | C | C |
| rs10021990 | 70391344 | G | T | T | - | - | T |
| rs11732968 | 70391429 | G | A | A | - | - | A |
| rs11732996 | 70391454 | G | A | A | A | A | A |
| rs66653481 | 70391536 | C | T | T | - | - | T |
| rs68096061 | 70391561 | G | A | A | - | - | A |
| rs60794737 | 70391616 | C | T | T | - | - | T |
| rs57568132 | 70391655 | T | C | C | - | - | C |
| rs58866998 | 70391688 | C | G | G | - | - | G |
| rs1864833 | 70391888 | G | A | A | A | A | A |
| rs71205990 | 70391979 | CT | C | - | - | - | C |
| rs1432323 | 70391986 | C | T | T | T | T | T |
| rs2331751 | 70392053 | A | G | G | G | G | G |
| rs2331752 | 70392062 | A | T | T | T | T | T |
| rs17671721 | 70392874 | C | A | A | A | A | A |
| rs17671739 | 70392967 | C | T | T | T | T | T |
| rs1494801 | 70393590 | A | G | G | - | - | G |
| rs12647143 | 70393660 | A | G | G | - | - | G |
| rs12644061 | 70393665 | C | T | T | - | - | T |
| rs12649893 | 70393680 | G | A | A | - | - | A |
| rs13139521 | 70393780 | C | T | T | T | T | T |
| rs7671101 | 70394240 | A | G | G | G | G | G |
| rs7692759 | 70394493 | G | C | C | C | C | C |
| rs7671691 | 70394500 | A | G | G | G | G | G |
| rs28861752 | 70394564 | G | C | C | C | C | C |
| rs62307047 | 70394991 | A | G | G | - | - | G |
| rs4694753 | 70395188 | T | C | C | - | - | C |
| rs4694754 | 70395338 | G | C | C | C | C | C |
| rs13101383 | 70395353 | G | C | C | C | C | C |
| rs2195847 | 70395596 | A | G | G | G | G | A/G |
| rs2195846 | 70395767 | T | G | T | - | - | G |
| rs2195845 | 70395803 | G | A | A | A | A | A |
| rs4164 | 70395821 | G | A | A | A | A | A |
| rs1603117 | 70395945 | A | G | G | - | - | G |
| rs9790349 | 70396060 | G | T | T | T | T | T |
| rs1579539 | 70396093 | C | A | A | - | - | A |
| rs1579538 | 70396137 | C | T | T | T | T | T |
| rs1816388 | 70396352 | T | C | C | - | - | C |
| rs1816387 | 70396415 | C | T | T | T | T | T |
| rs35501741 | 70396562 | T | C | C | - | - | C |
| rs62307049 | 70396681 | A | T | T | - | - | T |
| rs62307050 | 70396710 | A | G | G | - | - | G |
| rs62307051 | 70397007 | A | G | G | G | G | G |
| rs62307052 | 70397202 | G | C | C | - | - | C |
| rs72646793 | 70397236 | T | C | C | C | C | C |
| rs79863138 | 70397237 | G | A | A | A | A | A |
| rs28894679 | 70397266 | A | G | G | G | G | G |
| rs78532327 | 70397274 | C | T | T | T | T | T |
| rs28788541 | 70397323 | G | A | A | - | - | A |
| rs11723463 | 70397408 | G | A | A | A | A | A |
| rs28830586 | 70397424 | T | C | C | C | C | C |
| rs11736268 | 70397507 | A | G | G | - | - | G |
| rs145365794 | 70397714 | TTC | T | T | - | - | T |
| rs143944084 | 70397823 | CA | C | C | - | - | C |
| rs11737256 | 70397849 | T | C | C | - | - | C |
| rs11737211 | 70397899 | A | G | G | - | - | G |
| rs4560481 | 70397977 | T | C | C | - | - | C |
| rs10022597 | 70398467 | C | T | T | T | T | T |
| rs9999595 | 70398606 | G | T | T | T | T | T |
| rs1835836 | 70398769 | C | T | T | T | T | T |
| rs4296741 | 70398783 | G | A | A | A | A | A |
| rs1835835 | 70398857 | C | A | A | - | - | A |
| rs1835834 | 70399017 | C | A | A | - | - | A |
| rs7680733 | 70399573 | C | T | T | - | - | T |
| rs7679374 | 70399615 | G | A | A | - | - | A |
| rs12642964 | 70399643 | A | G | G | G | G | G |
| rs4527542 | 70399706 | G | A | A | A | A | A |
| rs4631113 | 70399774 | A | G | G | - | - | G |
| rs12643009 | 70399802 | A | G | G | - | - | G |
| rs4463133 | 70399877 | C | T | T | - | - | T |
| rs4434327 | 70399972 | C | T | T | T | T | T |
| rs4618378 | 70400027 | A | C | C | - | - | C |
| rs147775781 | 70400235 | A | AT | AT | - | - | AT |
| rs10028632 | 70400339 | C | A | A | A | A | A |
| rs9995529 | 70400391 | A | T | T | - | - | T |
| rs10028725 | 70400439 | T | C | C | - | - | C |
| rs10028726 | 70400450 | C | T | T | C/T | T | T |
| rs10028792 | 70400462 | C | A | A | A | A | A |
| rs376686064 | 70400524 | G | GT | GT | - | - | GT |
| rs9995731 | 70400631 | A | T | T | T | T | T |
| rs28769710 | 70400843 | A | G | G | - | - | G |
| rs62309089 | 70400884 | C | A | A | - | - | C |
| rs28831183 | 70400905 | G | A | A | - | - | A |
| rs111693490 | 70401025 | C | G | G | - | - | G |
| rs10026507 | 70401093 | A | G | G | - | - | G |
| rs10028760 | 70401103 | T | A | A | - | - | A |
| rs6840022 | 70401279 | A | T | T | - | - | T |
| rs143331726 | 70401303 | CT | C | C | - | - | C |
| rs6815630 | 70401513 | C | T | T | - | - | C |
| rs11730549 | 70401731 | C | T | T | - | - | T |
| rs35922051 | 70401741 | T | TA | - | - | - | - |
| rs28520630 | 70401751 | T | C | T | - | - | C |
| rs11722117 | 70401818 | G | A | A | - | - | A |
| rs11734865 | 70401824 | A | T | T | - | - | T |
| rs11722121 | 70401832 | G | A | A | - | - | A |
| rs11736579 | 70401931 | T | A | A | A | A | A |
| rs10049570 | 70402005 | G | A | A | A | A | A |
| rs10050282 | 70402042 | T | G | G | G | G | G |
| rs10029601 | 70402181 | A | T | T | T | T | T |
| rs5859188 | 70402217 | A | ATAAAT | - | - | - | - |
| rs10032036 | 70402246 | T | C | C | C | C | C |
| rs10029719 | 70402312 | A | C | C | C | C | C |
| rs2116563 | 70402805 | G | A | A | A | A | A |
| rs4694756 | 70402954 | T | C | T | - | - | C |
| rs4694757 | 70402958 | G | T | T | - | - | T |
| rs4694758 | 70403070 | T | C | C | C | C | C |
| rs4694759 | 70403427 | G | A | A | A | A | A |
| rs4694760 | 70403460 | C | T | T | T | T | T |
| rs4694761 | 70403503 | G | A | A | - | - | A |
| rs9990897 | 70403702 | T | G | G | G | G | G |
| rs9990996 | 70403764 | T | A | A | A | A | A |
| rs9991010 | 70403815 | T | A | A | - | - | A |
| rs138496545 | 70403954 | ACTCACCATAGT | A | - | - | - | - |
| rs373705597 | 70403982 | A | AT | - | - | - | - |
| rs139498379 | 70403989 | A | ACTT | - | - | - | - |
| rs10518062 | 70404001 | T | C | T/C | C | C | C |
| rs1368120 | 70404130 | C | T | T | T | T | T |
| rs10024407 | 70404144 | C | T | T | T | T | T |
| rs9993514 | 70404150 | T | C | C | C | C | C |
| rs1368119 | 70404196 | A | G | G | - | - | G |
| rs11725622 | 70404759 | G | A | A | A | A | A |
| rs11734977 | 70404889 | C | A | A | - | - | A |
| rs34104499 | 70404913 | AGT | A | A | - | - | A |
| rs34826606 | 70404918 | T | C | C | - | - | T/C |
| rs75969105 | 70404922 | T | C | C | - | - | C |
| rs17672445 | 70405360 | G | A | A | A | A | A |
| rs17616065 | 70405404 | C | T | T | T | T | T |
| rs4428351 | 70405545 | A | T | T | T | T | T |
| rs4619961 | 70405551 | A | C | C | C | C | C |
| rs28637000 | 70405916 | C | T | T | T | T | T |
| rs17616101 | 70405973 | G | A | A | A | A | A |
| rs17616137 | 70406203 | G | A | A | A | A | A |
| rs35795369 | 70406212 | CGT | C | - | - | - | C |
| rs199678039 | 70406234 | T | TGGAC | - | - | - | - |
| rs35421383 | 70406255 | GATAGATAA | G | - | - | - | - |
| rs66712465 | 70406423 | T | C | C | C | C | C |
| rs62309091 | 70406450 | C | T | T | T | T | T |
| rs7674468 | 70406575 | C | T | T | T | T | T |
| rs62309092 | 70406601 | C | T | T | - | - | T |
| rs17672577 | 70406851 | T | C | C | - | - | C |
| rs113592989 | 70406908 | T | C | C | - | - | C |
| rs112867065 | 70406918 | T | G | T/G | - | - | G |
| rs113170251 | 70406939 | G | T | G/T | - | - | T |
| rs1821191 | 70406957 | A | G | G | - | - | G |
| rs147595945 | 70406964 | A | G | A/G | - | - | G |
| rs185587531 | 70406966 | G | A | G/A | - | - | A |
| rs111510244 | 70407039 | A | G | G | - | - | G |
| rs111579901 | 70407054 | C | T | T | - | - | T |
| rs113556287 | 70407123 | C | G | G | - | - | G |
| rs66806835 | 70407232 | A | T | T | T | T | T |
| rs7680379 | 70407314 | A | T | T | T | T | T |
| rs62309093 | 70407359 | A | G | G | G | G | G |
| rs72849398 | 70407643 | T | A | A | - | - | A |
| rs111500630 | 70407762 | T | G | G | - | - | G |
| rs78891576 | 70407772 | T | C | C | - | - | C |
| rs28489327 | 70407784 | A | T | T | - | - | T |
| rs370591208 | 70407786 | AT | A | A | - | - | A |
| rs28676396 | 70407823 | A | C | C | - | - | C |
| rs28397036 | 70407993 | G | A | A | - | - | A |
| rs28808585 | 70408190 | C | T | T | - | - | T |
| rs146134372 | 70408244 | C | T | T | - | - | T |
| rs28806224 | 70408309 | A | G | A | - | - | - |
| rs59975217 | 70408436 | C | A | C/A | - | - | A |
| rs188519652 | 70408894 | A | G | - | - | - | - |
| rs36166047 | 70408922 | T | C | - | - | - | - |
| rs374400416 | 70408931 | G | A | G | - | - | - |
| rs58511569 | 70409574 | G | A | - | - | - | A |
| rs12331082 | 70409688 | T | C | C | - | - | C |
| rs59330455 | 70409752 | G | T | G | - | - | - |
| rs56703119 | 70409818 | G | A | A | - | - | - |
| rs59754150 | 70409826 | A | C | C | - | - | - |
| rs28708476 | 70410184 | T | C | C | C | C | C |
| rs28701673 | 70410273 | C | T | C | C | C | T |
| rs11249446 | 70411070 | T | C | C | C | C | C |
| rs11249447 | 70411081 | T | A | A | A | A | A |
| rs11249448 | 70411132 | A | C | C | C | C | C |
| rs6823947 | 70411395 | A | T | T | T | T | T |
| rs11421209 | 70411912 | G | GA | GA | - | - | G |
| rs6853919 | 70412096 | C | T | T | T | T | T |
| rs6853054 | 70412356 | G | A | A | A | A | A |
| rs6830906 | 70412437 | A | G | G | G | G | G |
| rs6836539 | 70412606 | T | G | G | G | G | G |
| rs11934774 | 70412912 | A | G | G | - | - | G |
| rs10005217 | 70413031 | T | C | C | C | C | C |
| rs10005218 | 70413032 | T | C | C | C | C | C |
| rs10013262 | 70413192 | G | C | C | C | C | C |
| rs10013567 | 70413508 | G | T | T | T | T | T |
| rs10005983 | 70413777 | T | C | T | T | T | C |
| rs10013867 | 70413804 | G | A | A | - | - | A |
| rs11940069 | 70413905 | G | T | T | - | - | T |
| rs376236663 | 70413952 | C | CT | CT | - | - | - |
| rs35871220 | 70413961 | A | AAG | AAG | - | - | - |
| rs11940110 | 70414029 | G | A | A | A | A | A |
| rs5859190 | 70414468 | GA | G | G | - | - | G |
| rs6850258 | 70414599 | T | C | C | - | - | C |
| rs2217584 | 70414640 | G | C | C | C | C | C |
| rs6818840 | 70414689 | G | A | A | - | - | A |
| rs6850688 | 70414796 | T | G | G | G | G | G |
| rs6825354 | 70414930 | C | A | A | A | A | A |
| rs6850489 | 70414931 | A | G | G | G | G | G |
| rs6824895 | 70415364 | G | A | A | A | A | A |
| rs6851807 | 70415522 | A | G | G | G | G | G |
| rs11941587 | 70415549 | G | T | G | - | - | T |
| rs6825504 | 70415655 | G | C | C | C | C | C |
| rs6857371 | 70415744 | T | G | G | G | G | G |
| rs6857548 | 70415787 | T | C | C | C | C | C |
| rs6825730 | 70415812 | G | A | A | A | A | A |
| rs6814084 | 70415877 | T | C | C | - | - | C |
| rs6832277 | 70415904 | C | A | A | A | A | A |
| rs6814289 | 70415954 | T | C | C | C | C | C |
| rs6857574 | 70416013 | A | G | G | G | G | G |
| rs2018730 | 70416121 | A | G | G | G | G | G |
| rs6832854 | 70416155 | C | T | T | T | T | T |
| rs6858206 | 70416321 | A | G | G | G | G | G |
| rs6832041 | 70416464 | G | T | T | T | T | T |
| rs6833517 | 70416480 | C | T | T | T | T | T |
| rs6815322 | 70416486 | T | G | G | G | G | G |
| rs6832081 | 70416526 | G | C | C | C | C | C |
| rs6832093 | 70416542 | G | A | A | A | A | A |
| rs6858812 | 70416600 | A | G | G | G | G | G |
| rs34970019 | 70416785 | C | T | T | T | T | T |
| rs10018101 | 70417188 | T | C | C | C | C | C |
| rs4694762 | 70417215 | C | T | T | - | - | T |
| rs34745402 | 70417253 | A | AG | - | - | - | A |
| rs4694763 | 70417286 | T | C | C | C | C | C |
| rs4694764 | 70417394 | A | C | C | - | - | C |
| rs4694765 | 70417495 | A | G | G | - | - | G |
| rs139587967 | 70417543 | AC | A | - | - | - | A |
| rs4694766 | 70417646 | G | A | A | A | A | A |
| rs7681809 | 70417733 | G | A | A | A | A | A |
| rs6846500 | 70418085 | C | A | A | - | - | A |
| rs6823097 | 70418179 | A | G | G | - | - | G |
| rs6846875 | 70418277 | C | T | T | T | T | T |
| rs6846876 | 70418279 | C | A | A | A | A | A |
| rs6823301 | 70418312 | A | G | G | - | - | G |
| rs6846898 | 70418313 | C | T | T | - | - | T |
| rs6845691 | 70418395 | G | A | A | A | A | A |
| rs6823668 | 70418471 | A | C | C | - | - | C |
| rs6845875 | 70418493 | G | A | A | - | - | A |
| rs6829148 | 70418650 | T | C | C | C | C | C |
| rs6824069 | 70418723 | A | T | T | - | - | T |
| rs6846528 | 70418849 | G | A | A | A | A | A |
| rs34891192 | 70418956 | G | GA | - | - | - | G |
| rs140623123 | 70419034 | A | AT | AT | - | - | A |
| rs4279106 | 70419361 | T | G | G | G | G | G |
| rs4402942 | 70419420 | T | C | C | C | C | C |
| rs7438680 | 70419458 | T | C | C | C | C | C |
| rs6600790 | 70419473 | C | T | T | T | T | T |
| rs7437806 | 70419567 | A | C | C | - | - | C |
| rs7441892 | 70419588 | C | A | A | A | A | A |
| rs79428624 | 70419720 | CA | C | C | - | - | C |
| rs2217585 | 70419884 | G | C | C | - | - | C |
| rs28831829 | 70419913 | C | T | T | - | - | T |
| rs1594592 | 70420001 | A | G | G | G | G | G |
| rs11721442 | 70420426 | G | A | A | - | - | A |
| rs11734234 | 70420487 | A | G | G | - | - | G |
| rs28896307 | 70420547 | G | A | A | - | - | A |
| rs68006105 | 70420639 | G | A | A | A | A | A |
| rs28786481 | 70420812 | A | G | G | - | - | G |
| rs28863264 | 70420888 | T | C | C | - | - | C |
| rs13103022 | 70420900 | C | T | T | - | - | T |
| rs56008539 | 70421022 | C | A | A | - | - | A |
| rs10518064 | 70421080 | G | T | T | - | - | T |
| rs11724830 | 70421227 | C | T | T | - | - | T |
| rs11732875 | 70421307 | G | T | T | - | - | T |
| rs11729137 | 70421369 | A | C | C | C | C | C |
| rs10011915 | 70421486 | G | A | A | - | - | A |
| rs10022094 | 70421528 | A | G | G | - | - | G |
| rs10000421 | 70421601 | C | A | A | A | A | A |
| rs10022191 | 70421618 | G | A | A | A | A | A |
| rs10000520 | 70421675 | C | T | T | - | - | T |
| rs10014530 | 70421689 | T | G | G | G | G | G |
| rs10022378 | 70421771 | G | A | A | - | - | A |
| rs139266337 | 70421898 | T | TTTTG | TTTTG | - | - | TTTTG |
| rs4694212 | 70421938 | T | C | T | - | - | C |
| rs4694767 | 70421986 | C | G | C/G | - | - | C/G |
| rs4694768 | 70422100 | C | G | G | - | - | G |
| rs4694213 | 70422141 | T | C | T | - | - | C |
| rs4694214 | 70422199 | A | T | T | - | - | T |
| rs4694769 | 70422296 | T | C | T | - | - | C |
| rs77885856 | 70422344 | C | T | T | - | - | T |
| rs10025460 | 70422704 | G | A | A | - | - | A |
| rs4425449 | 70422727 | A | G | G | - | - | A/G |
| rs34317830 | 70422745 | T | G | G | - | - | T/G |
| rs36118992 | 70422851 | T | C | C | - | - | C |
| rs6844058 | 70422890 | T | C | C | - | - | C |
| rs28718816 | 70422923 | C | A | A | - | - | A |
| rs28449651 | 70423008 | T | A | A | - | - | A |
| rs28570297 | 70423014 | T | C | C | - | - | C |
| rs28438681 | 70423051 | A | C | C | - | - | C |
| rs28445797 | 70423236 | T | C | C | C | C | C |
| rs10028160 | 70423325 | G | T | T | T | T | T |
| rs10028404 | 70423580 | G | A | A | - | - | A |
| rs10028422 | 70423633 | G | A | A | A | A | A |
| rs72633851 | 70424215 | A | C | A | - | - | C |
| rs62310371 | 70424226 | G | A | A | A | A | A |
| rs2116562 | 70424811 | G | A | A | A | A | A |
| rs28625519 | 70425058 | A | G | G | G | G | G |
| rs4974384 | 70425151 | G | C | C | C | C | C |
| rs7698435 | 70425165 | G | T | T | T | T | T |
| rs4974383 | 70425203 | A | C | C | C | C | C |
| rs4974382 | 70425220 | C | A | A | - | - | A |
| rs4974381 | 70425296 | T | C | C | C | C | C |
| rs4974380 | 70425337 | A | G | G | G | G | G |
| rs141654589 | 70425358 | CAAG | C | C | - | - | CAAG |
| rs4974379 | 70425430 | A | G | G | - | - | G |
| rs4974378 | 70425434 | A | G | G | G | G | G |
| rs6836099 | 70425826 | A | G | G | G | G | G |
| rs1989071 | 70426071 | A | T | T | - | - | T |
| rs4547863 | 70426147 | T | G | G | - | - | G |
| rs62307760 | 70426383 | T | A | A | - | - | A |
| rs62307761 | 70426388 | G | A | A | - | - | A |
| rs10212667 | 70426795 | G | T | T | T | T | T |
| rs10213258 | 70426866 | C | T | T | T | T | T |
| rs7695080 | 70426973 | C | T | T | - | - | T |
| rs144082687 | 70427192 | G | GAGTA | GAGTA | - | - | GAGTA |
| [rs144298528](https://www.ncbi.nlm.nih.gov/snp/rs144298528) | 70427272 | GA | G | G | - | - | G |
| rs4383681 | 70427362 | G | A | A | - | - | A |
| rs1594584 | 70427554 | A | G | G | - | - | G |
| rs4383679 | 70427571 | G | A | A | A | A | A |
| rs4576079 | 70427607 | T | C | C | C | C | C |
| rs4383678 | 70427623 | G | C | C | - | - | C |
| rs4464621 | 70427653 | C | T | T | T | T | T |
| rs78770629 | 70427888 | C | T | T | - | - | T |
| rs62307762 | 70427947 | C | A | A | - | - | A |
| rs66931301 | 70428039 | G | A | A | - | - | A |
| rs78854549 | 70428058 | A | G | G | G | G | G |
| rs77170327 | 70428064 | C | T | T | T | T | T |
| rs142287957 | 70428224 | GT | G | G | - | - | G |
| rs35966989 | 70428533 | T | TC | TC | - | - | T |
| rs2331681 | 70428558 | C | A | A | - | - | A |
| rs9999524 | 70428599 | A | G | G | - | - | G |
| rs2116559 | 70428840 | G | T | T | - | - | T |
| rs2116558 | 70428874 | C | T | T | - | - | T |
| rs2163657 | 70429211 | T | G | G | - | - | G |
| rs6836247 | 70429409 | A | G | G | G | G | G |
| rs2331682 | 70429421 | C | T | T | T | T | T |
| rs2331683 | 70429565 | C | G | G | G | G | G |
| rs11722143 | 70429748 | A | G | G | G | G | G |
| rs11722178 | 70429812 | A | G | G | - | - | G |
| rs11723881 | 70429896 | T | C | C | C | C | C |
| rs28819968 | 70430041 | G | A | A | A | A | A |
| rs28834733 | 70430155 | T | G | G | - | - | G |
| rs78267097 | 70430184 | CA | C | C | - | - | C |
| rs150112419 | 70430233 | A | AAAATGGACACATGT | - | - | - | - |
| rs1583241 | 70430536 | A | G | A | - | - | G |
| rs56405913 | 70430545 | GA | G | - | - | - | G |
| rs34955665 | 70430557 | TA | T | T | - | - | T |
| rs1821186 | 70430879 | C | T | T | T | T | T |
| rs1583240 | 70430909 | G | A | A | - | - | A |
| rs7439341 | 70430979 | A | G | G | - | - | G |
| rs7656491 | 70431063 | G | T | T | T | T | T |
| rs7681857 | 70431070 | A | T | T | T | T | T |
| rs7681889 | 70431130 | A | T | T | - | - | T |
| rs7658295 | 70431261 | C | T | T | T | T | T |
| rs7687501 | 70431410 | T | A | A | A | A | A |
| rs1432322 | 70431476 | A | G | G | G | G | G |
| rs7657318 | 70431505 | G | A | A | A | A | A |
| rs7657504 | 70431589 | G | A | A | A | A | A |
| rs55735079 | 70431614 | C | CTGT | CTGT | - | - | CTGT |
| rs12511454 | 70432006 | G | A | A | A | A | A |
|  |  |  |  |  |  |  |  |

^a^ - denotes genotype not available.

Supplementary Table 3. Haplotype 2 frequency in HGDP populations.

| Country | Population | Sample size | Frequency |
| --- | --- | --- | --- |
| Algeria | Mozabite | 30 | 18.3% |
| Bougainville | NAN Melanesian | 19 | 39.5% |
| Brazil | Surui | 21 | 9.5% |
| Brazil | Karitiana | 24 | 27.1% |
| Cambodia | Cambodian | 11 | 22.7% |
| Central African Republic | Biaka Pygmy | 32 | 50.0% |
| China | Oroqen | 10 | 10.0% |
| China | Hezhen | 9 | 16.7% |
| China | Lahu | 10 | 20.0% |
| China | Tujia | 10 | 20.0% |
| China | Han | 44 | 22.7% |
| China | Dai | 10 | 25.0% |
| China | Miaozu | 10 | 25.0% |
| China | Tu | 10 | 25.0% |
| China | Uygur | 10 | 25.0% |
| China | Yizu | 10 | 25.0% |
| China | Xibo | 9 | 27.8% |
| China | She | 10 | 30.0% |
| China | Naxi | 9 | 33.3% |
| China | Mongola | 10 | 35.0% |
| China | Daur | 9 | 44.4% |
| Colombia | Piapoco/Curripaco | 13 | 30.8% |
| Democratic Republic of Congo | Mbuti Pygmy | 15 | 30.0% |
| France | Basque | 24 | 31.3% |
| France | French | 29 | 37.9% |
| Israel | Bedouin | 48 | 29.2% |
| Israel | Druze | 47 | 35.1% |
| Israel | Palestinian | 51 | 36.3% |
| Italy | Bergamo | 13 | 30.8% |
| Italy | Tuscan | 8 | 43.8% |
| Italy | Sardinian | 28 | 48.2% |
| Japan | Japanese | 29 | 24.1% |
| Kenya | Bantu NE | 12 | 37.5% |
| Mexico | Pima | 25 | 10.0% |
| Mexico | Maya | 25 | 48.0% |
| Namibia | San | 6 | 25.0% |
| New Guinea | Papuan | 17 | 20.6% |
| Nigeria | Yoruba | 24 | 29.2% |
| Orkney Islands | Orcadian | 16 | 34.4% |
| Pakistan | Kalash | 25 | 24.0% |
| Pakistan | Brahui | 25 | 26.0% |
| Pakistan | Burusho | 25 | 26.0% |
| Pakistan | Makrani | 25 | 34.0% |
| Pakistan | Hazara | 24 | 37.5% |
| Pakistan | Balochi | 25 | 42.0% |
| Pakistan | Pathan | 23 | 43.5% |
| Pakistan | Sindhi | 25 | 44.0% |
| Russia | Russian | 25 | 42.0% |
| Russia Caucasus | Adygei | 17 | 38.2% |
| S. Africa Bantu | Bantu S. | 8 | 25.0% |
| Senegal | Mandenka | 24 | 29.2% |
| Siberia | Yakut | 25 | 38.0% |

Supplementary Table 4. Summary statistics in human *UGT2B4* upstream region for 1000G populations.

| Population | *n*^a^ | *S*^b^ | *θ_w_* | *π* | Tajima’s *D* | *P* |
| --- | --- | --- | --- | --- | --- | --- |
|  |  |  |  |  |  |  |
| ACB | 192 | 868 | 0.00272 | 0.00439 | 1.94534 | 0.0234 |
| ASW | 122 | 807 | 0.00275 | 0.00408 | 1.60093 | 0.0418 |
| BEB | 172 | 636 | 0.00203 | 0.00357 | 2.43542 | 0.0066 |
| CDX | 186 | 570 | 0.0018 | 0.00325 | 2.59327 | 0.0052 |
| CEU | 198 | 589 | 0.00184 | 0.00407 | 3.89787 | 0.0001 |
| CHB | 206 | 589 | 0.00183 | 0.00292 | 1.90906 | 0.0245 |
| CHS | 210 | 575 | 0.00178 | 0.00343 | 2.96664 | 0.0017 |
| CLM | 188 | 704 | 0.00222 | 0.00388 | 2.41044 | 0.0077 |
| ESN | 198 | 833 | 0.0026 | 0.00407 | 1.80303 | 0.0287 |
| FIN | 198 | 560 | 0.00175 | 0.00405 | 4.23145 | <0.0001 |
| GBR | 182 | 598 | 0.00189 | 0.00403 | 3.64083 | 0.0001 |
| GIH | 206 | 635 | 0.00197 | 0.00359 | 2.61885 | 0.0036 |
| GWD | 226 | 804 | 0.00245 | 0.00422 | 2.28367 | 0.0115 |
| IBS | 214 | 636 | 0.00196 | 0.00394 | 3.22073 | 0.0012 |
| ITU | 204 | 600 | 0.00186 | 0.00343 | 2.70036 | 0.0046 |
| JPT | 208 | 553 | 0.00171 | 0.00245 | 1.36298 | 0.0682 |
| KHV | 198 | 567 | 0.00177 | 0.00334 | 2.84523 | 0.002 |
| LWK | 198 | 816 | 0.00255 | 0.00434 | 2.25128 | 0.0119 |
| MSL | 170 | 816 | 0.00262 | 0.00381 | 1.47215 | 0.0527 |
| MXL | 128 | 625 | 0.00211 | 0.00408 | 3.10144 | 0.0006 |
| PEL | 170 | 688 | 0.00221 | 0.00374 | 2.25344 | 0.0095 |
| PJL | 192 | 601 | 0.00189 | 0.00391 | 3.43997 | 0.0004 |
| PUR | 208 | 754 | 0.00233 | 0.00366 | 1.81185 | 0.0305 |
| STU | 204 | 649 | 0.00202 | 0.00353 | 2.39543 | 0.0081 |
| TSI | 214 | 604 | 0.00186 | 0.00366 | 3.09682 | 0.0006 |
| YRI | 216 | 822 | 0.00253 | 0.00408 | 1.93684 | 0.0237 |

^a^number of chromosomes.

^b^number of segregating sites.

Supplementary Table 5. Primers for plasmid construction.

|  | Genome position | Primer | Anneal Temp (°C) |
| --- | --- | --- | --- |
| Segment 1 | chr4:70389165-70389799 | CAGTCCTCGAGCGCCTTGTCATTGAGAGGTA  CAGTCGGTACCGATCAAAGCAGCCACAAACA | 58 |
| Segment 2 | chr4:70389664-70390635 | CAGTCCTCGAGTGAGCCAATGTCTGCTAGCT  CAGTCGGTACCCTGCCTAGATCCTGCCAAAG | 59 |
| Segment 3 | chr4:70390572-70391811 | CAGTCCTCGAGAGAAAAAGCTGGTGTGACAGAT  CAGTCGGTACCTCTGTGGCTCTGTTGTCCAG | 59 |

Supplementary Table 6. rs11723463 genotype and *UGT2B4* expression in a LCL cohort.

| SRA ID | Sample ID | rs11723463 genotype | *UGT2B4* expression |
| --- | --- | --- | --- |
| SRR031811 | NA19210 | G/G | 324 |
| SRR031812 | NA19209 | A/G | 55 |
| SRR031813 | NA19098 | A/G | 15 |
| SRR031814 | NA19201 | A/G | 39 |
| SRR031815 | NA19153 | G/G | 146 |
| SRR031816 | NA19144 | A/G | 59 |
| SRR031817 | NA18909 | G/G | 29 |
| SRR031818 | NA19152 | G/G | 206 |
| SRR031819 | NA18511 | A/G | 105 |
| SRR031820 | NA19108 | G/G | 25 |
| SRR031821 | NA18498 | G/G | 498 |
| SRR031822 | NA18499 | G/G | 48 |
| SRR031823 | NA18520 | A/A | 115 |
| SRR031824 | NA19238 | A/A | 120 |
| SRR031825 | NA19239 | G/G | 68 |
| SRR031826 | NA19098 | A/G | 0 |
| SRR031827 | NA19144 | A/G | 136 |
| SRR031828 | NA19201 | A/G | 103 |
| SRR031829 | NA19210 | G/G | 237 |
| SRR031830 | NA19153 | G/G | 210 |
| SRR031831 | NA18909 | G/G | 32 |
| SRR031832 | NA19147 | G/G | 30 |
| SRR031833 | NA19152 | G/G | 352 |
| SRR031834 | NA18499 | G/G | 51 |
| SRR031835 | NA19209 | A/G | 69 |
| SRR031836 | NA19143 | A/G | 43 |
| SRR031837 | NA19257 | A/G | 32 |
| SRR031838 | NA18861 | A/G | 98 |
| SRR031839 | NA19131 | A/G | 24 |
| SRR031840 | NA19192 | A/G | 187 |
| SRR031841 | NA18916 | G/G | 23 |
| SRR031842 | NA19222 | A/G | 69 |
| SRR031843 | NA19225 | A/G | 10 |
| SRR031844 | NA18913 | A/G | 123 |
| SRR031845 | NA18853 | G/G | 0 |
| SRR031846 | NA18862 | A/A | 18 |
| SRR031847 | NA19147 | G/G | 32 |
| SRR031848 | NA19143 | A/G | 38 |
| SRR031849 | NA19190 | A/A | 12 |
| SRR031850 | NA18501 | G/G | 178 |
| SRR031851 | NA18856 | A/G | 90 |
| SRR031852 | NA18912 | G/G | 95 |
| SRR031853 | NA19102 | A/G | 0 |
| SRR031854 | NA19119 | G/G | 9 |
| SRR031855 | NA19171 | G/G | 115 |
| SRR031856 | NA19200 | G/G | 19 |
| SRR031857 | NA18517 | G/G | 156 |
| SRR031858 | NA19128 | A/A | 25 |
| SRR031859 | NA19130 | A/G | 68 |
| SRR031860 | NA19238 | A/A | 94 |
| SRR031861 | NA19239 | G/G | 41 |
| SRR031862 | NA18504 | A/G | 82 |
| SRR031863 | NA18516 | G/G | 150 |
| SRR031864 | NA18522 | A/G | 336 |
| SRR031865 | NA19093 | A/G | 86 |
| SRR031866 | NA19172 | A/G | 0 |
| SRR031867 | NA19140 | G/G | 25 |
| SRR031868 | NA18508 | G/G | 63 |
| SRR031869 | NA18519 | A/G | 48 |
| SRR031870 | NA19127 | A/G | 20 |
| SRR031871 | NA18505 | A/G | 66 |
| SRR031872 | NA19138 | G/G | 200 |
| SRR031873 | NA18502 | A/A | 40 |
| SRR031874 | NA19114 | A/G | 14 |
| SRR031875 | NA18507 | G/G | 68 |
| SRR031876 | NA18504 | A/G | 261 |
| SRR031877 | NA19193 | A/G | 131 |
| SRR031878 | NA18516 | G/G | 176 |
| SRR031879 | NA18511 | A/G | 242 |
| SRR031880 | NA18520 | A/A | 135 |
| SRR031881 | NA18498 | G/G | 474 |
| SRR031882 | NA19131 | A/G | 79 |
| SRR031883 | NA19108 | G/G | 57 |
| SRR031884 | NA19190 | A/A | 68 |
| SRR031885 | NA18861 | A/G | 45 |
| SRR031886 | NA19257 | A/G | 19 |
| SRR031887 | NA19192 | A/G | 244 |
| SRR031888 | NA19222 | A/G | 98 |
| SRR031889 | NA18916 | G/G | 34 |
| SRR031890 | NA18862 | A/A | 30 |
| SRR031891 | NA18853 | G/G | 168 |
| SRR031892 | NA18913 | A/G | 75 |
| SRR031893 | NA19203 | G/G | 55 |
| SRR031894 | NA19101 | A/G | 56 |
| SRR031895 | NA19116 | A/G | 111 |
| SRR031896 | NA19099 | A/G | 65 |
| SRR031897 | NA18517 | G/G | 87 |
| SRR031898 | NA18498 | G/G | 137 |
| SRR031899 | NA19160 | G/G | 0 |
| SRR031900 | NA19140 | G/G | 43 |
| SRR031901 | NA19127 | A/G | 39 |
| SRR031902 | NA18505 | A/G | 112 |
| SRR031903 | NA18502 | A/A | 68 |
| SRR031904 | NA18508 | G/G | 112 |
| SRR031905 | NA19138 | G/G | 214 |
| SRR031906 | NA18519 | A/G | 93 |
| SRR031907 | NA18858 | G/G | 114 |
| SRR031908 | NA19225 | A/G | 14 |
| SRR031909 | NA18504 | A/G | 79 |
| SRR031910 | NA19160 | G/G | 0 |
| SRR031911 | NA18507 | G/G | 71 |
| SRR031912 | NA19114 | A/G | 6 |
| SRR031913 | NA18516 | G/G | 206 |
| SRR031914 | NA19204 | G/G | 101 |
| SRR031915 | NA18510 | G/G | 105 |
| SRR031916 | NA18871 | A/G | 10 |
| SRR031917 | NA18486 | G/G | 79 |
| SRR031918 | NA18870 | A/G | 25 |
| SRR031919 | NA19159 | G/G | 57 |
| SRR031920 | NA19160 | G/G | 0 |
| SRR031921 | NA19101 | A/G | 63 |
| SRR031922 | NA19128 | A/A | 64 |
| SRR031923 | NA19130 | A/G | 47 |
| SRR031924 | NA19203 | G/G | 74 |
| SRR031925 | NA18517 | G/G | 315 |
| SRR031926 | NA18517 | G/G | 68 |
| SRR031927 | NA19099 | A/G | 116 |
| SRR031928 | NA18504 | A/G | 54 |
| SRR031929 | NA18516 | G/G | 108 |
| SRR031930 | NA18522 | A/G | 211 |
| SRR031931 | NA19093 | A/G | 126 |
| SRR031932 | NA19172 | A/G | 0 |
| SRR031933 | NA18498 | G/G | 139 |
| SRR031934 | NA19116 | A/G | 156 |
| SRR031935 | NA18871 | A/G | 21 |
| SRR031936 | NA19204 | G/G | 147 |
| SRR031937 | NA19159 | G/G | 122 |
| SRR031938 | NA18486 | G/G | 108 |
| SRR031939 | NA19171 | G/G | 109 |
| SRR031940 | NA18912 | G/G | 82 |
| SRR031941 | NA18523 | G/G | 168 |
| SRR031942 | NA19193 | A/G | 88 |
| SRR031943 | NA19160 | G/G | 0 |
| SRR031944 | NA18852 | G/G | 157 |
| SRR031945 | NA18855 | A/A | 68 |
| SRR031946 | NA18870 | A/G | 32 |
| SRR031947 | NA18510 | G/G | 259 |
| SRR031948 | NA19200 | G/G | 28 |
| SRR031949 | NA18912 | G/G | 153 |
| SRR031950 | NA18501 | G/G | 247 |
| SRR031951 | NA18856 | A/G | 62 |
| SRR031952 | NA18505 | A/G | 120 |
| SRR031953 | NA18502 | A/A | 34 |
| SRR031954 | NA18855 | A/A | 40 |
| SRR031955 | NA18852 | G/G | 73 |
| SRR031956 | NA18858 | G/G | 63 |
| SRR031957 | NA19171 | G/G | 127 |
| SRR031958 | NA19137 | A/G | 82 |
| SRR031959 | NA18523 | G/G | 127 |
| SRR031960 | NA19137 | A/G | 101 |
| SRR031961 | NA19200 | G/G | 47 |
| SRR031962 | NA18502 | A/A | 49 |
| SRR031963 | NA18856 | A/G | 88 |
| SRR031964 | NA18505 | A/G | 400 |
| SRR031965 | NA18501 | G/G | 75 |
| SRR031966 | NA18856 | A/G | 27 |
| SRR031967 | NA18912 | G/G | 78 |
| SRR031968 | NA19102 | A/G | 0 |
| SRR031969 | NA19119 | G/G | 42 |
| SRR031970 | NA19171 | G/G | 52 |
| SRR031971 | NA19200 | G/G | 14 |

Supplementary Table 7. Primers for PCR and resequencing.

|  | Genome position | PCR Primer | Anneal Temp (°C) | Resequencing Primer |
| --- | --- | --- | --- | --- |
| Amplicon 1 | chr4:70389165-70390412 | cgccttgtcattgagaggta  GTTGAGGGTTTGTGGCAATC | 58 | GTGAATACAGAAACAAAAAG  CACCTGCTGCTTTACTTTG |
| Amplicon 2 | chr4:70390154-70391177 | GGGCACTCAGTAGACAAAT  CCATTCATGTTTGGTATCAT | 59 | AACACTTGACATTTTGACTG  AACAAAGTGCAATAAAATAAGGTA |
| Amplicon 3 | chr4:70390909-70391811 | TGCCAAACACAGATTGACAAG  TCTGTGGCTCTGTTGTCCAG | 59 | TCTGGCTCTGTGGCTCTGTTGTCC  GCACAAAAGCAAAGGCACTGATG |

Supplementary Table 8. Primers for mutagenesis.

| SNP position in chr4 | dbSNP ID | Primers^a^ | Anneal Temp (°C) |
| --- | --- | --- | --- |
| 70390723 | rs17615130 | GGCACACAATTTAACCTAAAATTCATACTT  AGAGATTATTTTTAAAAATTGGTTGAAATTGG | 65 |
| 70390777 | rs66862535 | TACAAAGCTATGCAAGGTCTCCAAT  CAGTACATATGGGGTTTTATTTTAATTTACAG | 65 |
| 70390795 | rs72646780 | TAAAATAAAACCCCATATATACTGTACAAAGCTATG  ATTTACAGGAAAAATAACCAAGTATTTAAGAAAGC | 66 |
| 70391246 | rs10011713 | TTataattatttatatggaaaaGacttattaaaaaattaaGCTC  atttttGGAGACTCATTTTTTGGG | 65 |
| 70391344 | rs10021990 | GGCAGGTGCATAGATAACTGAGTAGATAATC  ATTTTTTCACATTACACATTCAATGCACA | 67 |
| 70391429 | rs11732968 | gttttttgCTCTCTTATATGTCTCATCAACGA  attatgatgtagtccctgtgctcaagc | 68 |
| 70391454 | rs11732996 | tgagcacagggactaTatcataatgttTT  agcagctcacatgtttaTATTTAAACAAGTTG | 66 |
| 70391536 | rs66653481 | cgcccagccTAGAGCCCTTT  aggtggctcacaccgttaatccc | 70 |
| 70391561 | rs68096061 | gctgggattaaTggtgtgagcc  actttgggaggccaaggc | 67 |
| 70391616 | rs60794737 | ccaggatggtctAgatctcctgac  ctaacacggtgaaaccccatctc | 66 |
| 70391655 | rs57568132 | ttttgtatttttGgtagagatggggtttca  Aattagccaggcttggtggc | 68 |
| 70391688 | rs58866998 | TACAGGCGCCcGCCACCAAGC  GTCCCAGCTACTCCGGAG | 67 |

^a^The target site underlined.

Supplementary Table 9. Primers for 3C-qPCR.

| Primer name | Primer sequence | Primer location | Restriction fragment location | Target element |
| --- | --- | --- | --- | --- |
| Target primer 1 | TCTCAGGCACTTATGATGTTGAGAC | chr4:70357902-70357926 | chr4:70357345-70357942 |  |
| Target primer 2 | TGAATGAATGGCCAGTACCTTC | chr4:70359135-70359156 | chr4:70357942-70359175 |  |
| Target primer 3 | AAGAGTCAATTTTAGAGAACTCTTATCTCC | chr4:70362323-70362352 | chr4:70359175-70362369 | *UGT2B4* promoter |
| Target primer 4 | GTTCTTTTAGTTGCGATATATGTGTCAA | chr4:70364655-70364682 | chr4:70362369-70364696 |  |
| Target primer 5 | GGCCATTATTCAATATCTGGCC | chr4:70370658-70370679 | chr4:70364696-70370685 |  |
| Target primer 6 | CAAATATCCTCAACAACACACTGG | chr4:70372530-70372553 | chr4:70370685-70372580 |  |
| Target primer 7 | GAAAACTCCATCGTCTCGGG | chr4:70373035-70373054 | chr4:70372580-70373059 |  |
| Target primer 8 | TCTGTGCTTTTCAGATCATATTCAAA | chr4:70375611-70375636 | chr4:70373059-70375661 |  |
| Target primer 9 | GCATTTGCTGAATTTACTTATCACCT | chr4:70379652-70379677 | chr4:70375661-70379682 |  |
| Target primer 10 | GTTCTTTTGCTGACTGCCAGG | chr4:70383781-70383801 | chr4:70379682-70383807 |  |
| Target primer 11 | ATTTACTCAGATTTTAAAGGACGGTC | chr4:70384898-70384923 | chr4:70383807-70384932 |  |
| Constant primer | GCAGATGGTGATGATGTTTGG | chr4:70397238-70397258 | chr4:70390613-70397294 | enhancer |

Supplementary Table 10. Primers for ChIP-qPCR.

| SNP ID | Primer |
| --- | --- |
| rs68096061 | GATGTAGTCCCTGTGCTCAAGCA  GTTTCACCGTGTTAGCCAGGAT |
| rs66862535 | TTAAATTGTGTGCCAGAGATTATTTTTA  ACAAACACTTGACATTTTGACTGGC |

Supplementary Table 11. Probes for EMSA.

| SNP ID | Allele | Probe^a^ |
| --- | --- | --- |
| rs68096061 | G allele | GGTGGCTCACACCGTTAATCCCAGCAC  GTGCTGGGATTAACGGTGTGAGCCACC |
|  | A allele | GGTGGCTCACACCATTAATCCCAGCAC  GTGCTGGGATTAATGGTGTGAGCCACC |
| rs66862535 | A allele | AATTGGAGACCTTACATAGCTTTGTAC  GTACAAAGCTATGTAAGGTCTCCAATT |
|  | G allele | AATTGGAGACCTTGCATAGCTTTGTAC  GTACAAAGCTATGCAAGGTCTCCAATT |

^a^The target site underlined.

Supplementary Figure 1. Distribution of estimated age for 412 SNPs in CEU core haplotype.


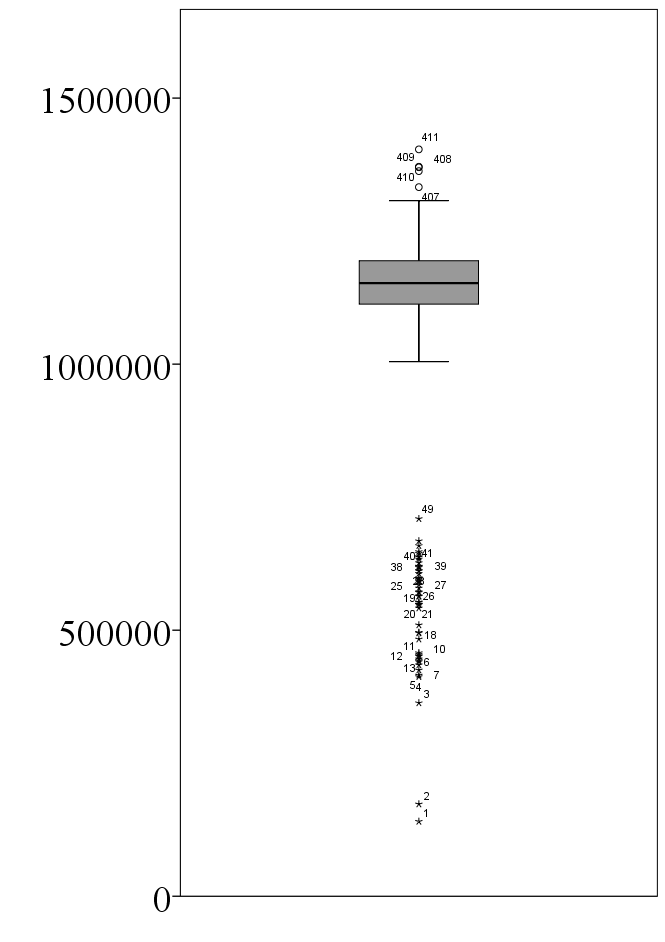


Supplementary Figure 2. Frequency of core haplotype 2 in history. The *x* axis indicates different time period in history while the *y* axis represents the haplotype 2 frequency. BP denotes before present. All data is expressed as mean ± standard deviation (SD).


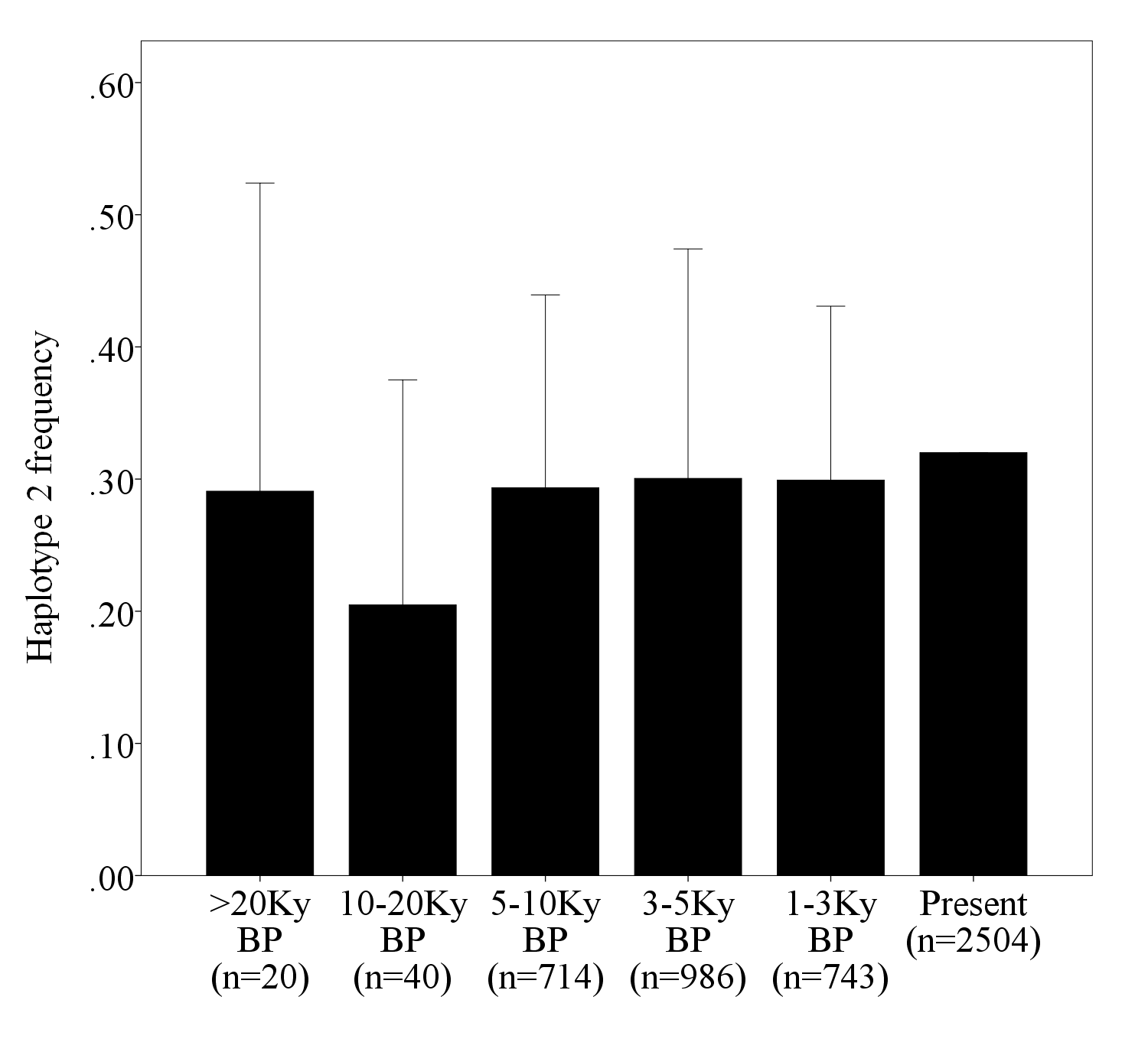


Supplementary Figure 3. Relative promoter activity of the two haplotypes. Each bar represents one plasmid. All data is displayed as mean ± SD.


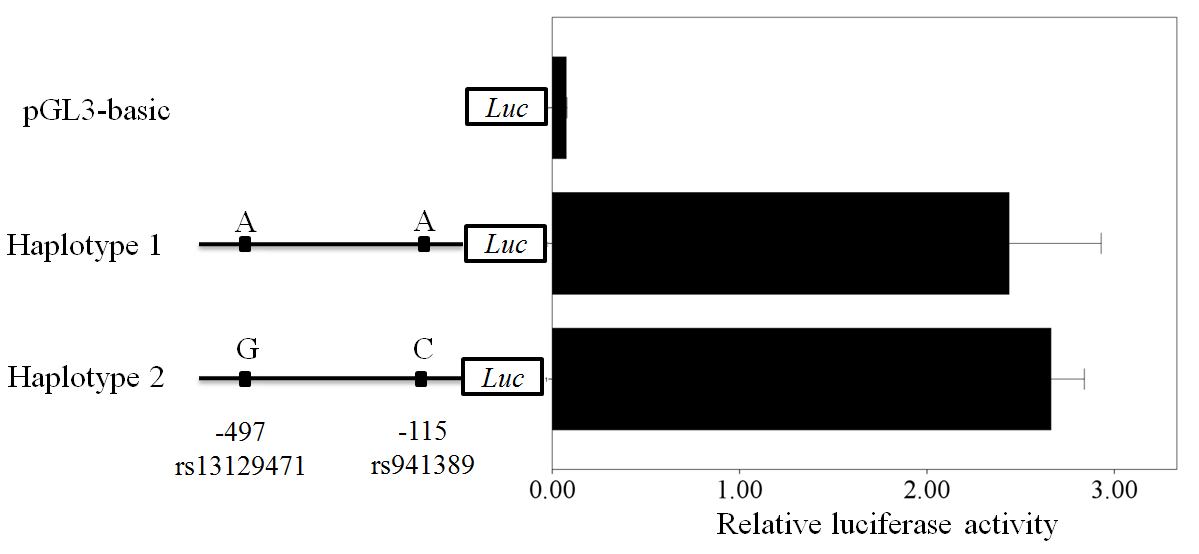


Supplementary Figure 4. Genome annotation for *UGT2B4* upstream region. The *x* axis denotes chr4 coordinate while *y* axis indicates *π* (a)*,* Tajima’s *D* (b) and GWAVA score (c), respectively. The arrow indicates the segment chr4:70389000-70392000.


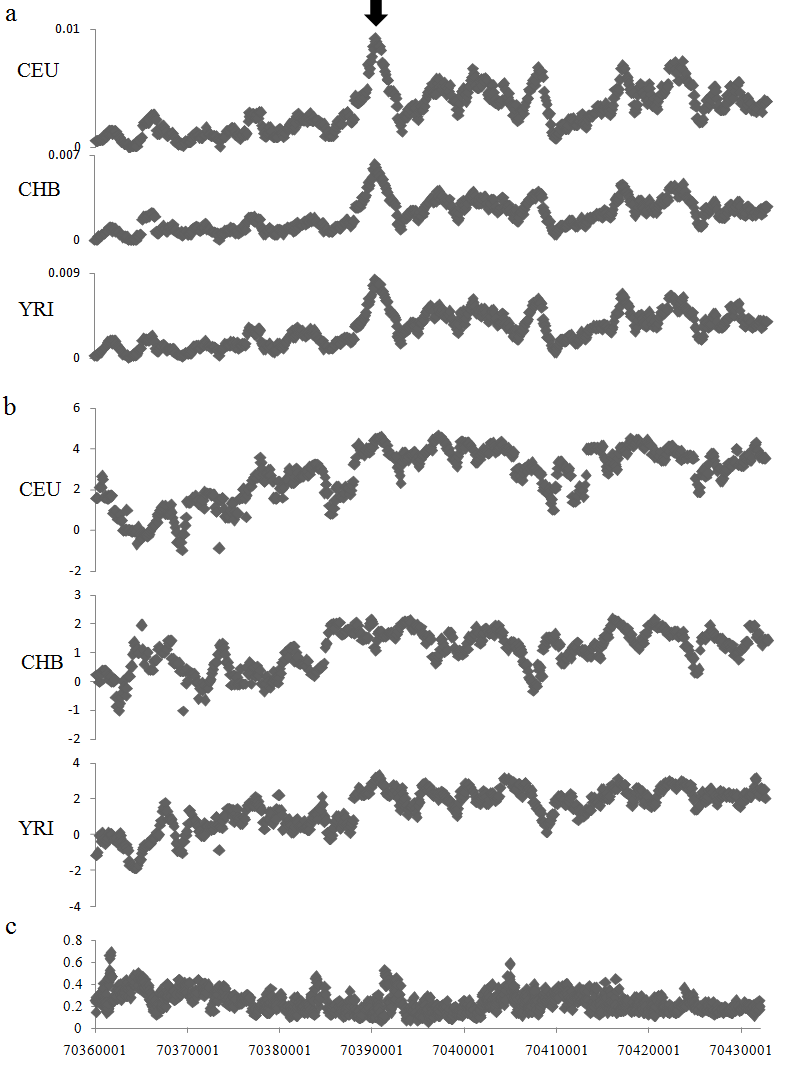


Supplementary Figure 5. Visual genotype for chr4:70389000-70392000 region in 25 East Asian. Each column indicates one SNP while each row denotes one individual. Blue, red, yellow, and grey represent homozygous of common allele, heterozygous, homozygous of rare allele, and missing data, respectively.


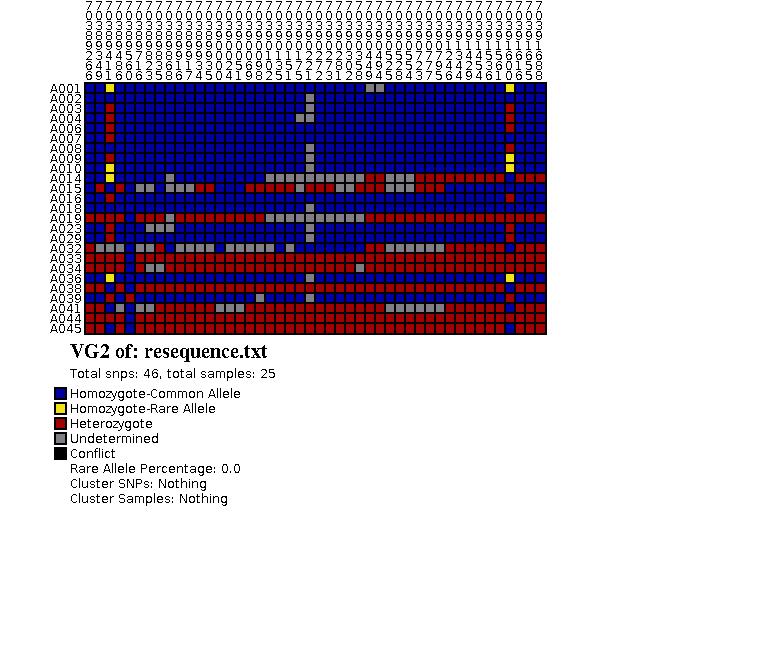


Supplementary Figure 6. Histone modification in liver cell line HepG2 for the segment surrouding rs66862535 and rs68096061. The left and right yellow lines indicate the location of rs66862535 and rs68096061, respectively.


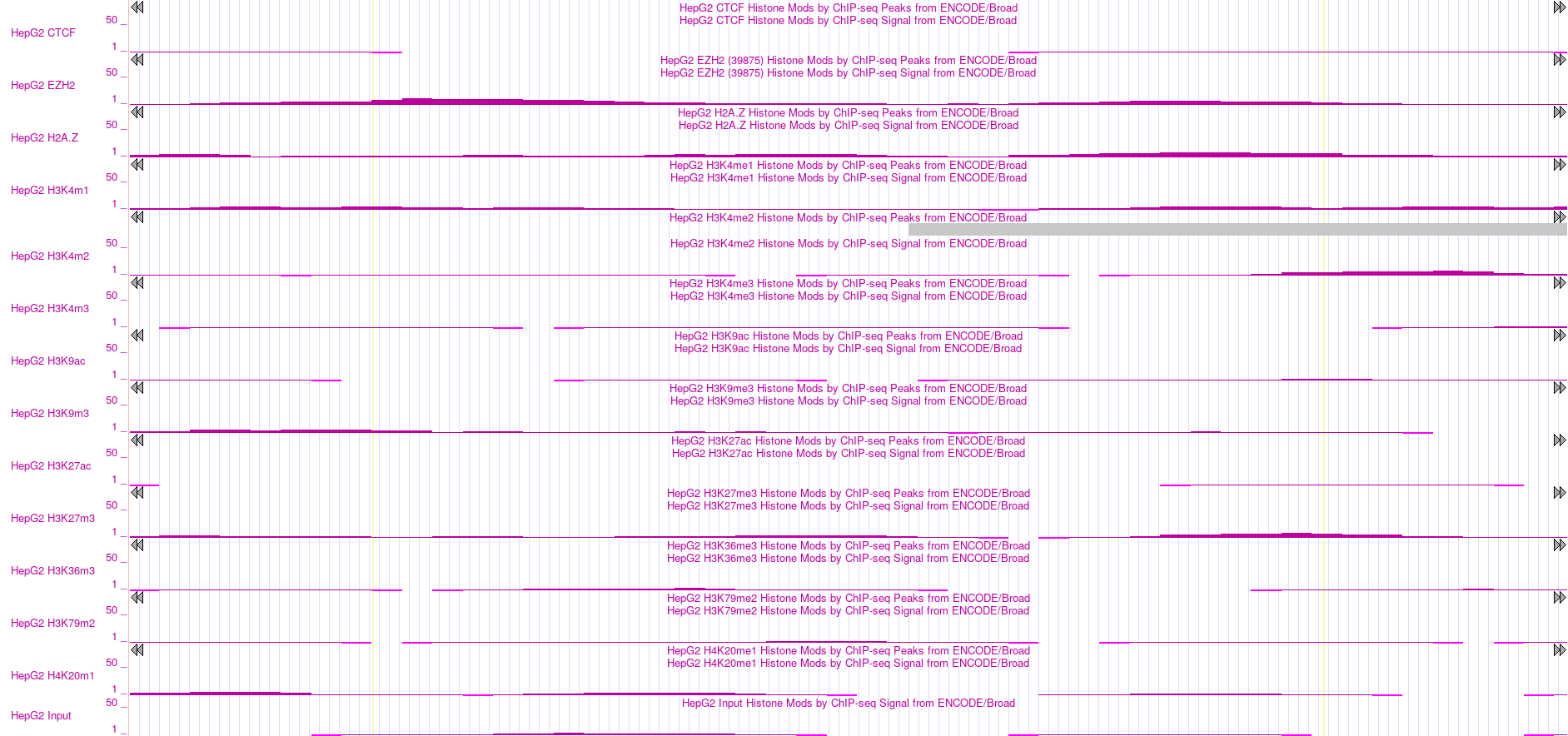


Supplementary Figure 7. Association between rs11723463 genotype and *UGT2B4* expression in YRI population. The *x* axis denotes rs11723463 genotype while *y* axis indicates the *UGT2B4* expression (log transformed with base 2). The sample size for each group is shown in bracket.


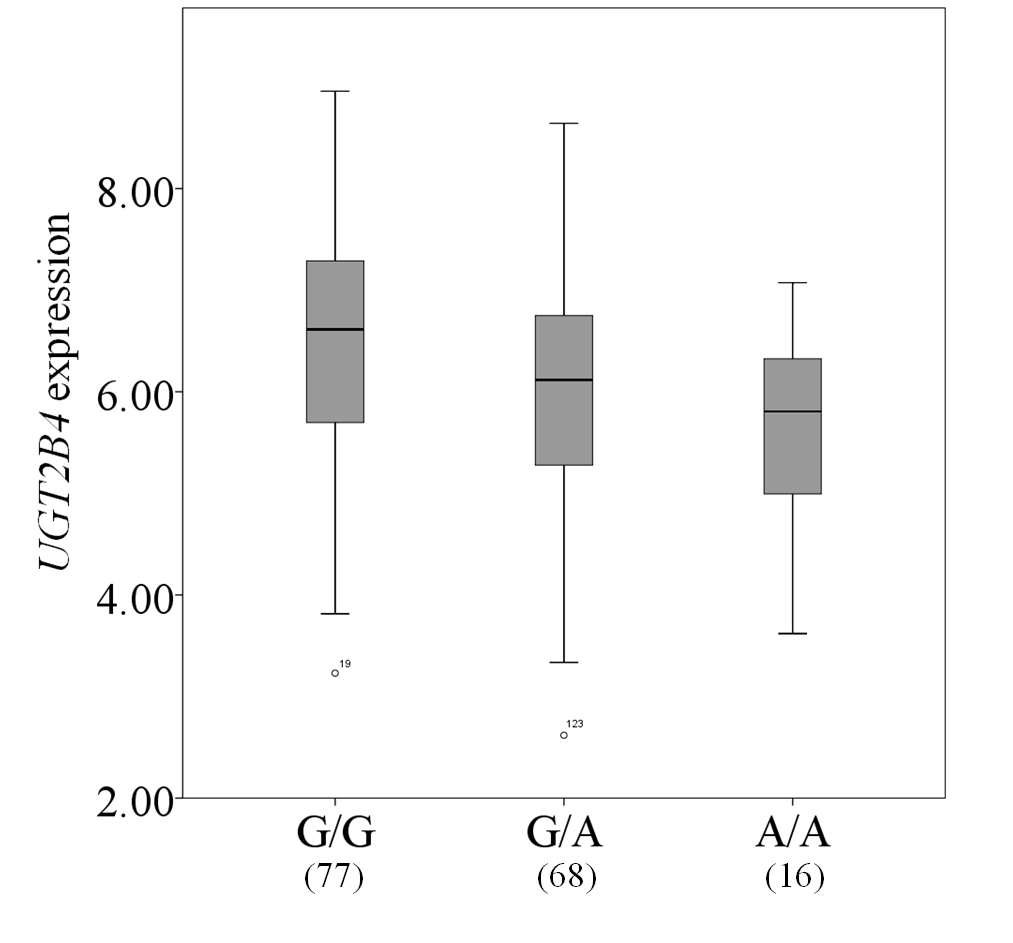


Supplementary Figure 8. Association between rs11723463 genotype and *UGT2B4* expression in lung (a), heart-atrial appendage (b), heart-left ventricle (c), adipose-visceral (d), adipose-subcutaneous (e), breast mammary (f) and artery coronary (g) from GTEx project. The *x* axis indicates genotype, while *y* axis shows gene expression. The number 69531690 indicates the position of rs11723463 in chr4 (relative to human genome build 38).The sample size for each group is shown in bracket.


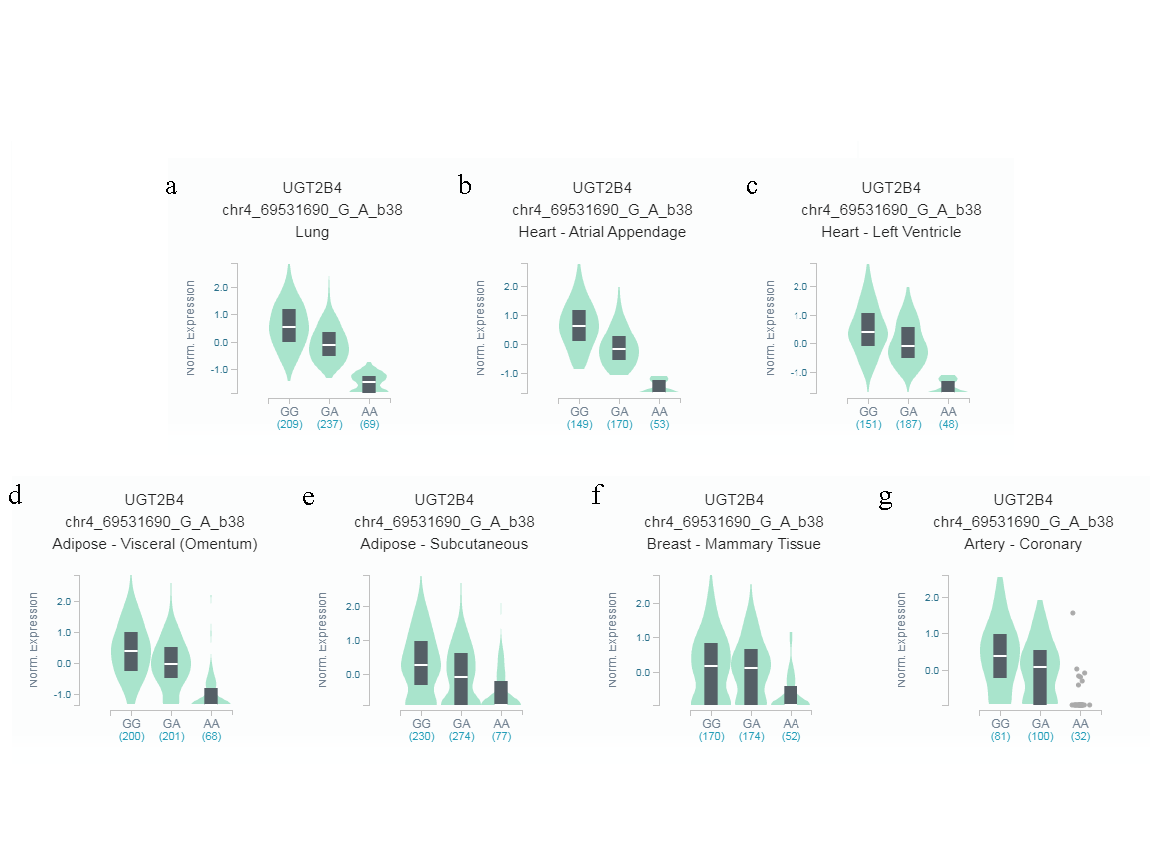


Supplementary Figure 9. Association between rs11723463 genotype and *UGT2B4* expression in lung squamous cell carcinoma (LUSC, a), lung adenocarcinoma (LUAD, b) and breast invasive carcinoma (BRCA, c). The *x* axis indicates genotype, while *y* axis shows *UGT2B4* expression. In *x* axis, A and a denote alleles G and A, respectively.


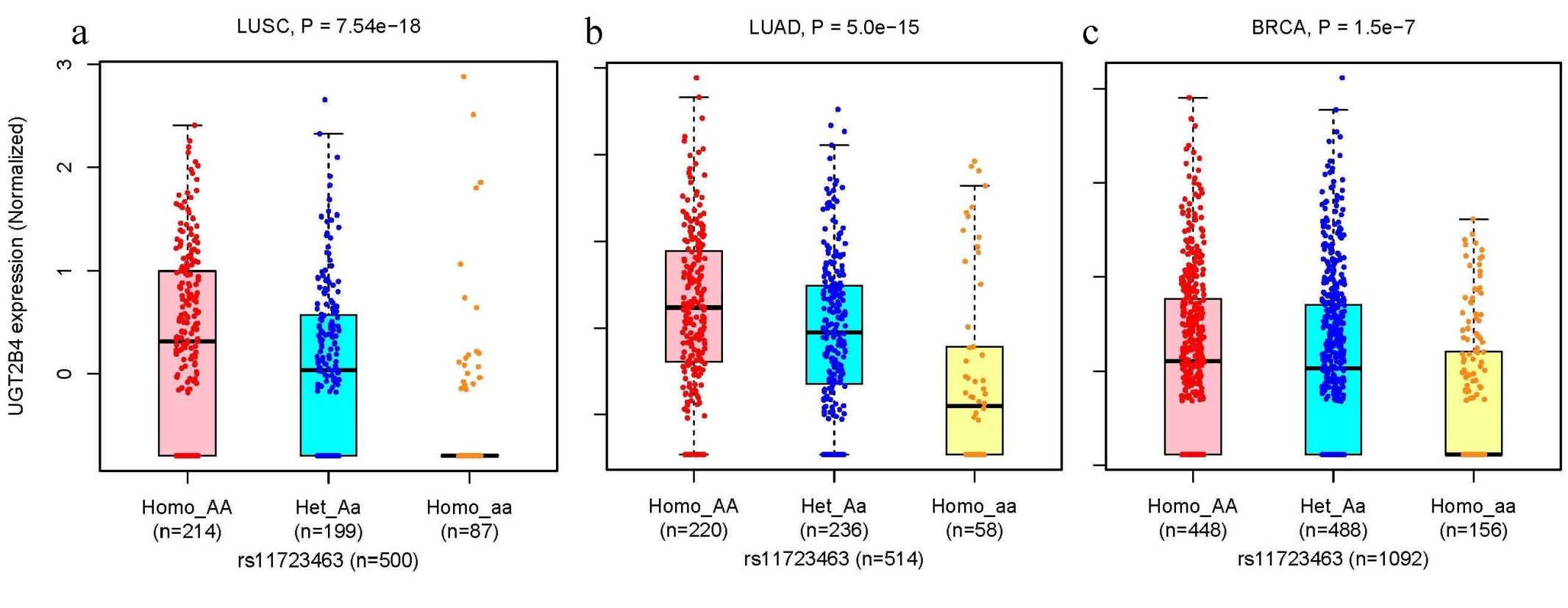


Supplementary Figure 10. Enrichment of the chromatin region surrounding rs66862535 (a) and rs68096061 (b) in HepG2 cell line. The *y* axis represents relative enrichment. The result is normalized by input and the data is expressed as mean ± SD.


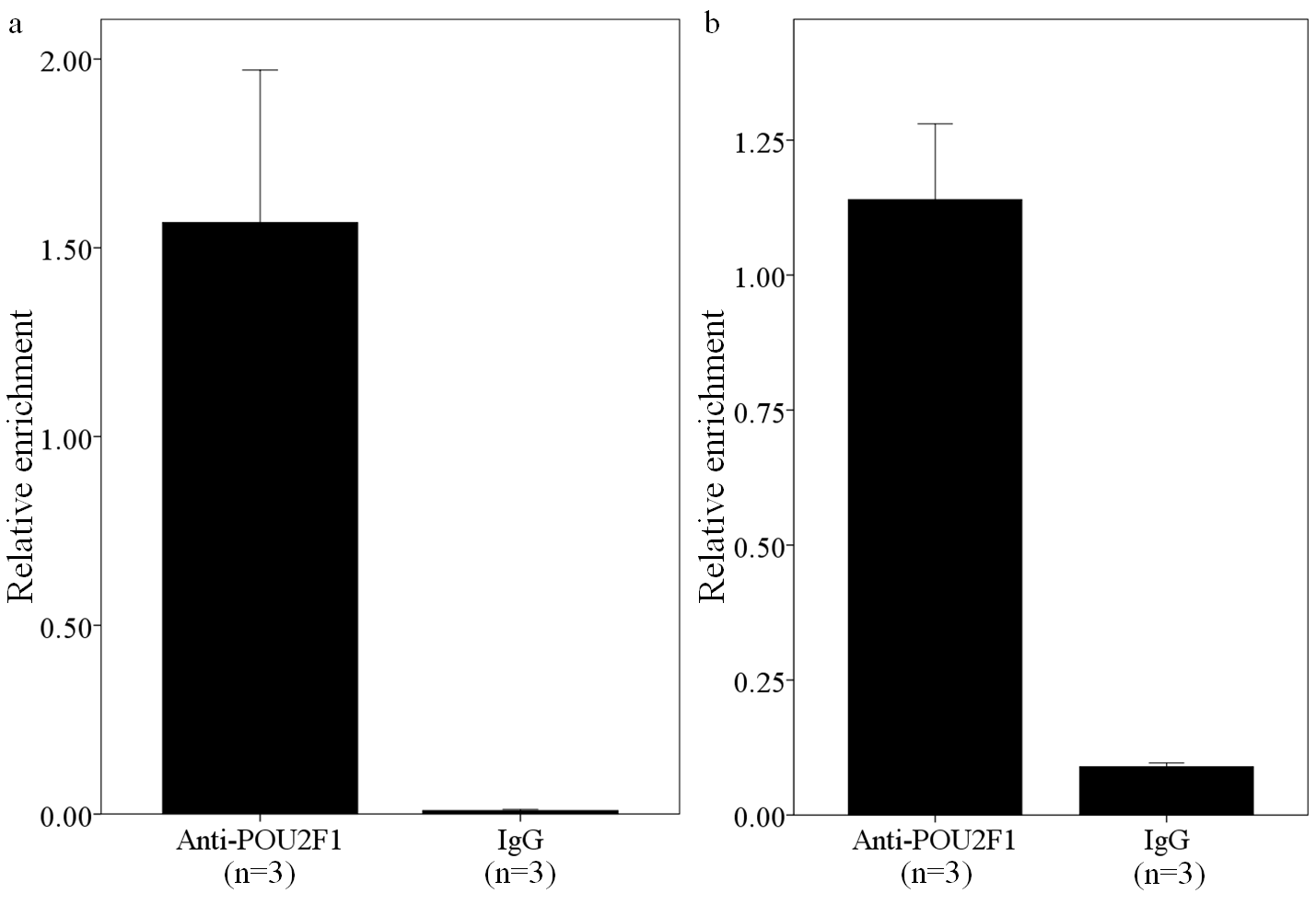


Supplementary Figure 11. Binding affinity difference between rs66862535 (a) and rs68096061 (b) alleles. The top line indicates different alleles for each SNP. NE denotes nuclear extracts and the arrow points out the position of protein-probe complex.

**
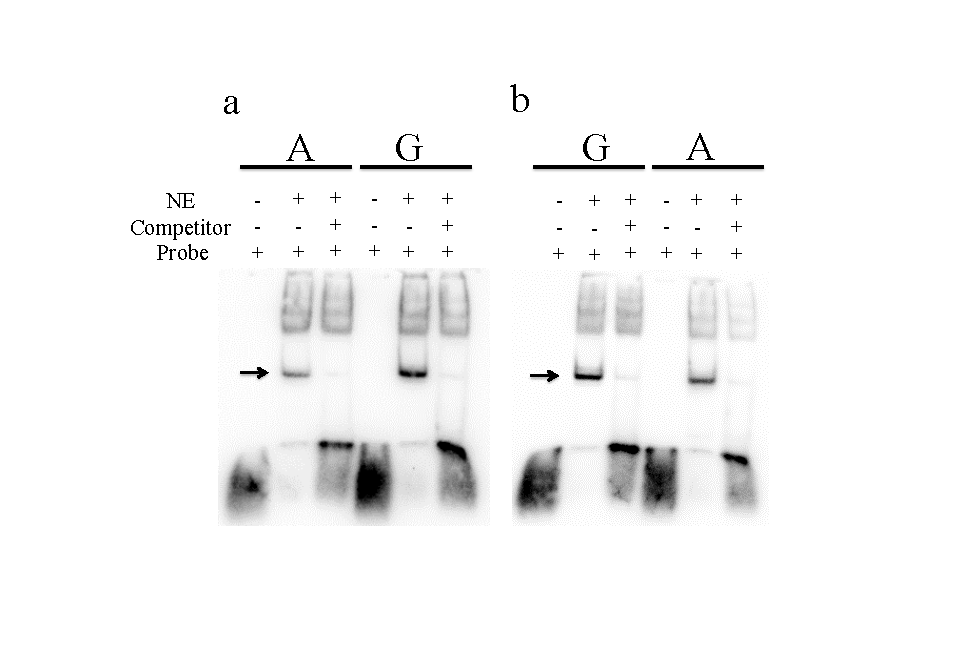
**

Supplementary Figure 12. Boxplot of *UGT2B4* expression between normal and BRCA tissues from TCGA project. The blue and red colors indicate normal and tumor tissues, respectively.


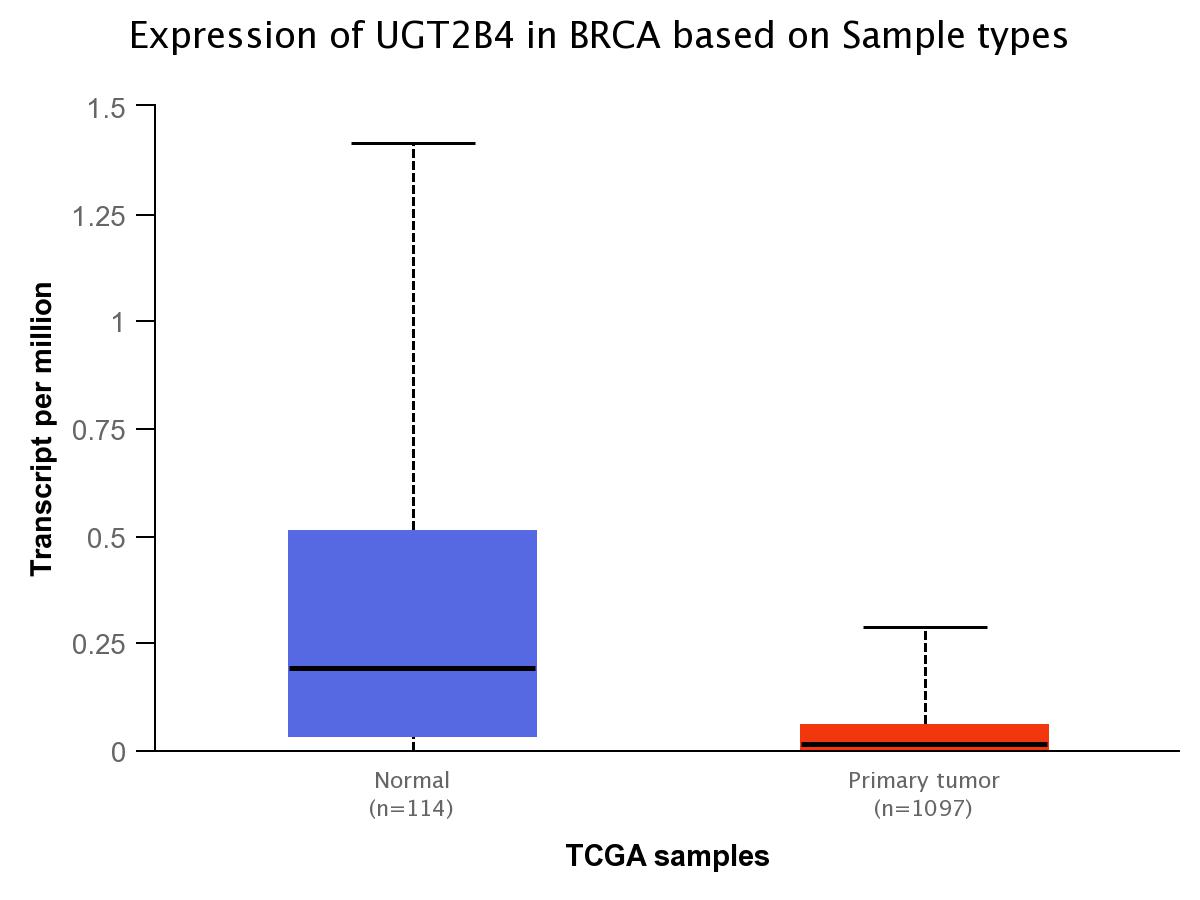


Supplementary Figure 13. Association between esv3600896 genotype and *UGT2B28* (a) or *UGT2B11* (b) expression in YRI. The *x* axis denotes esv3600896 genotype while *y* axis indicates the gene expression (log transformed with base 2). Ins and Del indicate insertion and deletion, respectively. For *UGT2B28* expression, 1 is added to the raw value before log transformation. The sample size for each group is shown in bracket.


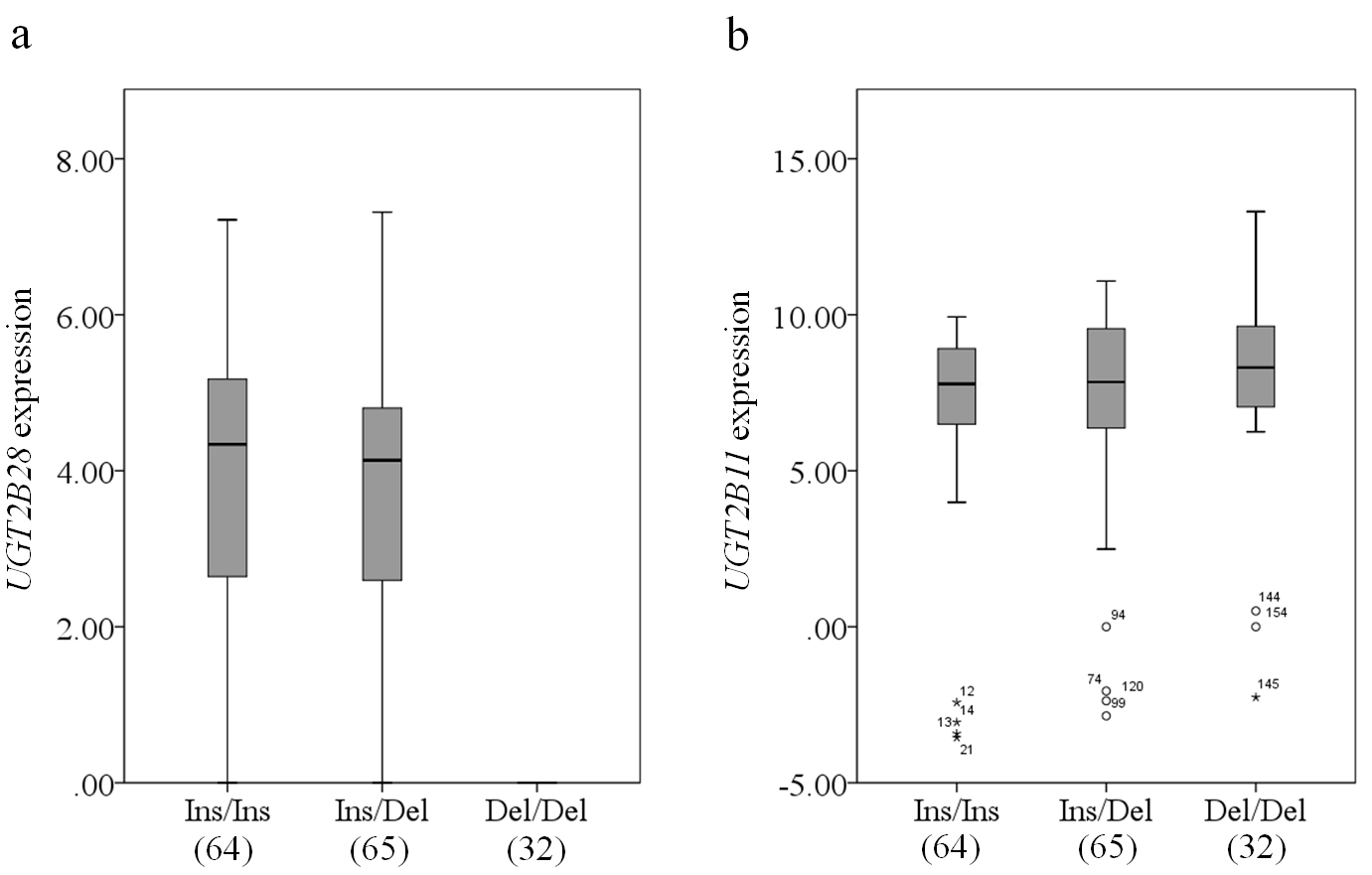

Supplement: Supplementary file 3 — Supplementary Information 3. [file 41598_2023_29682_MOESM3_ESM.docx]
